# Colquhounia Root Tablet Promotes Autophagy and Inhibits Apoptosis in Diabetic Nephropathy by Suppressing CD36 Expression *In Vivo* and *In Vitro*

**DOI:** 10.1155/2023/4617653

**Published:** 2023-08-16

**Authors:** Han Li, Baiju Wang, Changbao Wu, Dandan Xie, Jizhen Li, Na Wang, Hanwen Chen, Lei Liu

**Affiliations:** ^1^Department of Clinical Medicine, Jining Medical University, Jining, Shandong 272013, China; ^2^Department of General Medicine, Affiliated Hospital of Jining Medical University, Jining, Shandong 272029, China; ^3^Department of Cardiology, Wenshang County People's Hospital, Jining, Shandong 272501, China

## Abstract

**Methods:**

Rat models of DN were established using streptozotocin (STZ). The primary metabolic parameters were assessed. The pathological changes of the rat kidney were investigated, and RNA sequencing was performed for each group. Renal tissue apoptosis was detected using the TUNEL assay. In rats and high glucose- (Hg-) induced HK-2 cells, RT-qPCR and western blot were used to analyze the expression of related genes and proteins. Hg medium was used to establish the diabetic kidney environment. The CCK-8 assay and flow cytometry were used to assess cell viability and apoptosis, respectively. Transmission electron microscopy was used to evaluate autophagy in vitro.

**Results:**

CRT treatment significantly reduced albuminuria and renal tissue damage in DN rats. Furthermore, CRT administration inhibited apoptosis and promoted autophagy in DN rat kidney tissues. CRT downregulated CD36 expression and activated the adenosine monophosphate-activated protein kinase (AMPK) signaling pathway in DN rat kidney tissues. CRT intervention inhibited Hg-induced apoptosis and reversed autophagy in HK-2 cells. Moreover, overexpression of CD36 suppressed the beneficial effects of CRT.

**Conclusions:**

Our study is the first to report that CRT inhibited apoptosis and promoted autophagy *in vivo* and *in vitro*, which was achieved by reducing CD36 expression and activating the AMPK pathway. Therefore, CRT may be an effective drug to treat DN.

## 1. Introduction

Diabetic nephropathy (DN) is a serious microvascular complication caused by diabetes mellitus (DM) [1]. Its clinical manifestations are a persistent increase in proteinuria excretion and/or a progressive decrease in the glomerular filtration rate, leading eventually to end-stage renal disease [2–5]. The treatment strategy for DN is based on a combination of therapeutic options such as controlling proteinuria and regulating blood glucose (BG) and blood pressure [6]. Unfortunately, the current treatment strategy is limited and unsatisfactory [7]. Therefore, some DN patients have started to turn to traditional Chinese medicine (TCM) for treatment [8].

Colquhounia root tablet (CRT) is a Chinese patent medicine made from the peeled root of *Tripterygium hypoglaucum* (Lévl.) Hutch [9]. It contains several terpenoids, alkaloids, phenolic acids, and lactones, such as triptolide and epicatechin [9]. According to clinical studies, CRT is an efficient and low-toxicity drug with anti-inflammatory, analgesic, immunosuppressive, and hormone regulatory effects [10]. CRT has shown good results in the treatment of rheumatic system diseases such as rheumatoid arthritis and systemic lupus erythematosus [11]. Growing evidence has shown the potential of CRT for alleviating DN [12]. In particular, Ma et al. [13] reported that CRT can reverse the immune-inflammatory system imbalance to treat DN. However, the mechanism underlying DN treatment requires further evaluation.

Increased apoptosis and loss of autophagy due to insulin resistance are important pathogenic mechanisms of DN [14]. The degree of apoptosis is closely related to the degree of renal function deterioration. Bax is a proapoptotic gene, while Bcl-2 inhibits apoptosis [15]. Autophagy plays a defensive role in DN development. Beclin-1 is one of the key regulatory fractions that initiate autophagy, and LC3 is an autophagosome marker protein [16]. p62 is a substrate protein for selective autophagy that accumulates when autophagy is blocked [17]. AMPK is an important kinase involved in cellular energy sensing and cell signaling regulation during autophagy. In a high glucose (Hg) environment such as in diabetes, AMPK activity is inhibited, resulting in impaired autophagy [18]. The system of autophagy/apoptosis control may be induced through common upstream signals, leading to the merging or mutual rejection of autophagy and apoptosis [19].

Our study investigated the mechanisms and examined the effects of CRT on DN *in vitro* and *in vivo* using the DN rat and HK-2 cell models. CRT suppressed albuminuria and reduced damage to HK-2 cells. The mechanism of action was through a reduction in CD36 expression and activation of the AMPK pathway. CD36 is a glycoprotein on the cell surface of the family of B scavenger receptors that play a role in inflammation, apoptosis, lipid accumulation, and renal fibrosis [20]. Our results showed that CRT inhibits apoptosis and promotes autophagy by suppressing the expression of CD36 and activating the AMPK pathway. Therefore, CRT is a promising drug for treating DN.

## 2. Materials and Methods

### 2.1. Animals and Treatment

Male Sprague–Dawley (SD) rats (6–8 weeks old, *n* = 30, weight 180–220 g) were purchased from Jinan Pengyue Experimental Animal Breeding Co. (Jinan, China). The study protocol was approved by the Ethics Committee of Medical Science Research of the Affiliated Hospital of Jining Medical University (approval number: 2022B053) and complied with the Guide for the Care and Use of Laboratory Animals proposed by the Chinese National Institutes of Health. Rats were placed in a 12 h light/dark cycle and had free access to water and food. Following acclimatization for a week, 30 SD rats were randomly divided into three groups (*n* = 10 for every group): the Con group—the normal control rats; the DN group—rats with diabetic nephropathy; the CRT group—DN rats treated with 600 mg/kg CRT. Streptozotocin (60 mg/kg, Solarbio, Beijing, China) was used to establish the DN model, with sodium citrate as a control. After 72 hours, the BG levels were measured, and rats with BG levels ≥ 16.65 mmol/L were included in the observation subjects. Two weeks later, rats with urinary microalbumin (mALB) ≥ 30 mg/L showed that the DN model was set up successfully. In the CRT group, rats were given 600 mg/kg body weight of CRT (0.18 g/tablet, Pharmaceutical Factory of the Chongqing Academy of Chinese Materia Medica, Chongqing, China) by gavage per day for 8 weeks after model establishment (refer to literature [21] for dose information). The Con and DN groups were intragastrically administered equal amounts of saline. In the end, all rats were dissected and sampled.

### 2.2. Detection of Biochemical Indicators

BG levels were measured with an Accu-Chek Active blood glucose meters (Roche Diagnostic, Basel, Switzerland). Rat urine was collected using metabolic cages, and mALB was determined using an immunoturbidimetric assay. The kidney index (KI) and body weight of each experimental animal were measured. Blood was collected by cardiac puncture to determine serum creatinine (Scr) levels.

### 2.3. Histopathological Evaluation

The renal tissues were fixed with 4% paraformaldehyde, dehydrated with ethanol, embedded in paraffin and sectioned, and next stained with hematoxylin and eosin (HE), periodic acid-Schiff (PAS), and MASSON to observe histopathological changes.

### 2.4. TUNEL Assay

According to the manufacturer's instructions, apoptosis was assessed in kidney tissues using the TUNEL Apoptosis Detection Kit (Roche Diagnostic). The images were taken under a fluorescent microscope (Nikon, Tokyo, Japan) with blue nuclei of normal cells and green nuclei of apoptosis-positive cells.

### 2.5. RNA-Sequencing

All RNA-sequencing (RNA-seq) experiments were conducted at Berry Genomics Co., Ltd. (Beijing, China). The details can be found in the Supplementary Materials (available here).

### 2.6. Cell Culture and Treatment

We used the human proximal renal tubular epithelial (HK-2) cell line (CL-0109, Wuhan Plantronics Life Sciences Co., Ltd., Wuhan, China) for *in vitro* experiment validation. The cells were incubated in Dulbecco's modified Eagle medium with low glucose (5.6 mmol/L), 10% fetal bovine serum, and 1% penicillin/streptomycin. The Hg environment was established with 30 mmol/L of glucose.

### 2.7. Cell Transfection

The CD36 plasmid (Wuhan GeneCreate Biological Engineering Co., Ltd., Wuhan, China) was transfected using Lipo6000™ Transfection Reagent (Beyotime, Shanghai, China) as directed by the manufacturer. In six-well plates, HK-2 cells were transfected using the Lipo6000™ transfection reagent with the CD36 plasmid or a negative control with pcDNA3.1 when the cell density reached approximately 80%.

### 2.8. CCK-8 Assay

HK-2 cells were seeded in 96-well plates and incubated with different concentrations of CRT, and then the cells were incubated with Hg for 24 h. Then CCK-8 reagent (Beyotime) was added and incubated in the incubator for 2 h. Finally, we measured the optical density values of each hole at 450 nm.

### 2.9. Flow Cytometric Analysis

HK-2 cells in each group were collected and mixed with Annexin V-FITC and propidium iodide without light for 15 min. Apoptotic cells were distinguished by flow cytometry, and the apoptosis rate was calculated.

### 2.10. Transmission Electron Microscopy

HK-2 cells were fixed with a 2.5% glutaraldehyde solution at 4°C for 3 days and then placed in 1% osmium tetroxide for 30 minutes. Uranyl acetate and lead citrate were used to stain the cells. Finally, we observed autophagy under transmission electron microscopy (FEI, Hillsboro, OR, USA).

### 2.11. Quantitative Real-Time PCR

Total RNA was obtained by the TRIzol (Solarbio) method. mRNA was reverse-transcribed to cDNA, and CD36 expression was detected by real-time fluorescence quantitative PCR. *β*-Actin was used as the internal reference, and the 2^−△△Ct^ method was used for calculations. The sequences of each primer are shown in the Supplementary Materials.

### 2.12. Western Blot Analysis

Total proteins were extracted with the protein lysis buffer (Beyotime) and quantified using a BCA protein quantification kit (Beyotime). The protein samples were isolated by polyacrylamide gel electrophoresis, and the proteins on the gel were transferred to polyvinylidene fluoride membranes. The membranes were closed with 5% skim milk for 1 h and treated with the corresponding specific primary antibody and horseradish peroxidase-labeled secondary antibody in sequence. Finally, images were obtained using the Ultrasensitive ECL Chemiluminescence Kit (Beyotime), and bands were analyzed using ImageJ software (National Institutes of Health, Bethesda, MD, USA).

### 2.13. Statistical Analysis

Statistical analysis was conducted with GraphPad Prism version 5.0.0 (GraphPad Software, San Diego, CA, USA). Data were shown as mean ± SD. Data between two groups were compared using *t*-test, and data from multiple groups were compared using a one-way ANOVA. *P* < 0.05 was regarded as statistically significant.

## 3. Results

### 3.1. Effects of CRT on Primary Metabolic Parameters and Renal Histopathological Changes in DN Rats

**Figure 1:**
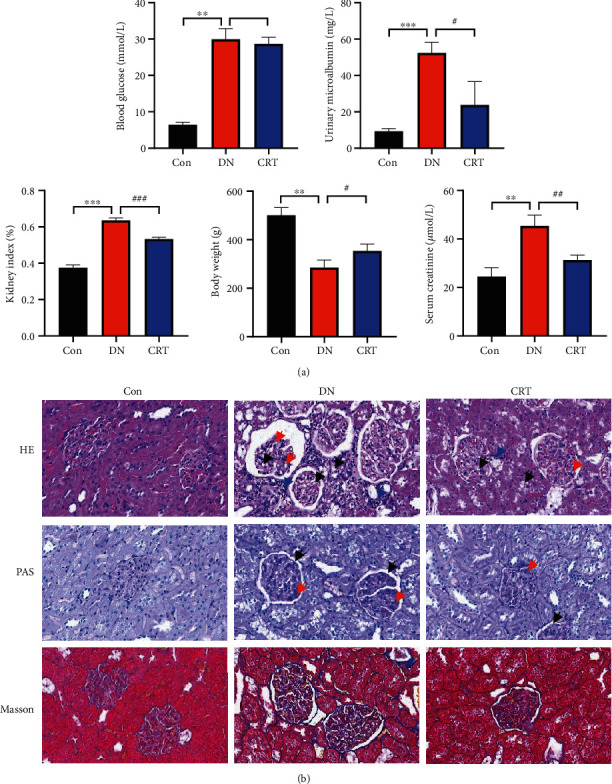
Effects of CRT on the levels of primary metabolic parameters and renal histopathological changes in DN rats. (a) Effects of CRT on blood glucose, urinary microalbumin, serum creatinine, body weight, and kidney index of DKD rats. (b) Pathology changes of the kidney tissues in different groups were revealed by HE, PAS, and MASSON staining. Scale bar = 50 *μ*m.  ^∗^*P* < 0.05,  ^∗∗^*P* < 0.01,  ^∗∗∗^*P* < 0.001, compared with the Con group; ^#^*P* < 0.05, ^##^*P* < 0.01, ^###^*P* < 0.001, compared with the DN group.

The DN group had higher BG, mALB, Scr, and KI levels and significantly lower body weight compared to the Con group (Figure 1(a)). Compared with the rats in the DN group, those in the CRT-treated groups had significantly reduced mALB, Scr, and KI levels and increased body weight. However, CRT administration did not alleviate BG levels. HE staining revealed glomerular vascular atrophy (red arrow), cytoplasmic vacuolar degeneration (black arrow), and inflammatory infiltration (blue arrow) in the DN rats. PAS staining revealed a thickened glomerular basement membrane (black arrow) and increased mesangial matrix (red arrow) in DN rats. Furthermore, the slightly increased interstitial fibrosis in the DN group was observed by MASSON staining. Administration of CRT was effective in reducing histopathological changes in diabetic kidneys.

### 3.2. CRT Inhibited Apoptosis and Promoted Autophagy in DN Rats

**Figure 2:**
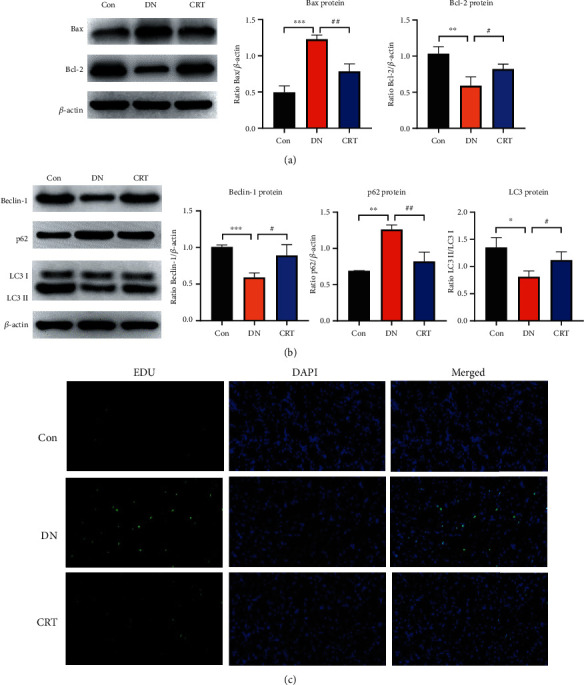
CRT inhibited renal apoptosis and promoted autophagy in DN rats. (a) Western blot analysis of Bax and Bcl-2 in kidney tissues of rats. (b) Western blot analysis of Beclin-1, p62, and LC3 in kidney tissues of rats. (c) Apoptosis in different rat experimental groups was revealed using the TUNEL assay. Scale bar = 20 *μ*m. Data represent the mean ± SEM for 3–5 rats.  ^∗^*P* < 0.05,  ^∗∗^*P* < 0.01,  ^∗∗∗^*P* < 0.001, compared with the Con group; ^#^*P* < 0.05, ^##^*P* < 0.01, ^###^*P* < 0.001, compared with the DN group.

Western blot analysis revealed that compared with the Con group, the DN group exhibited a decrease in the expression of autophagy-related proteins LC3II and Beclin-1 and an increase in the expression of p62. In contrast, CRT intervention significantly reversed the changes in these proteins (Figure 2(a)). Apoptosis was increased in the DN group, which was improved after CRT treatment (Figure 2(b)). Meanwhile, TUNEL staining showed that CRT inhibited apoptosis (Figure 2(c)).

### 3.3. CRT Regulates the Expression of CD36 and AMPK Signaling Pathways in DN Rats

**Figure 3:**
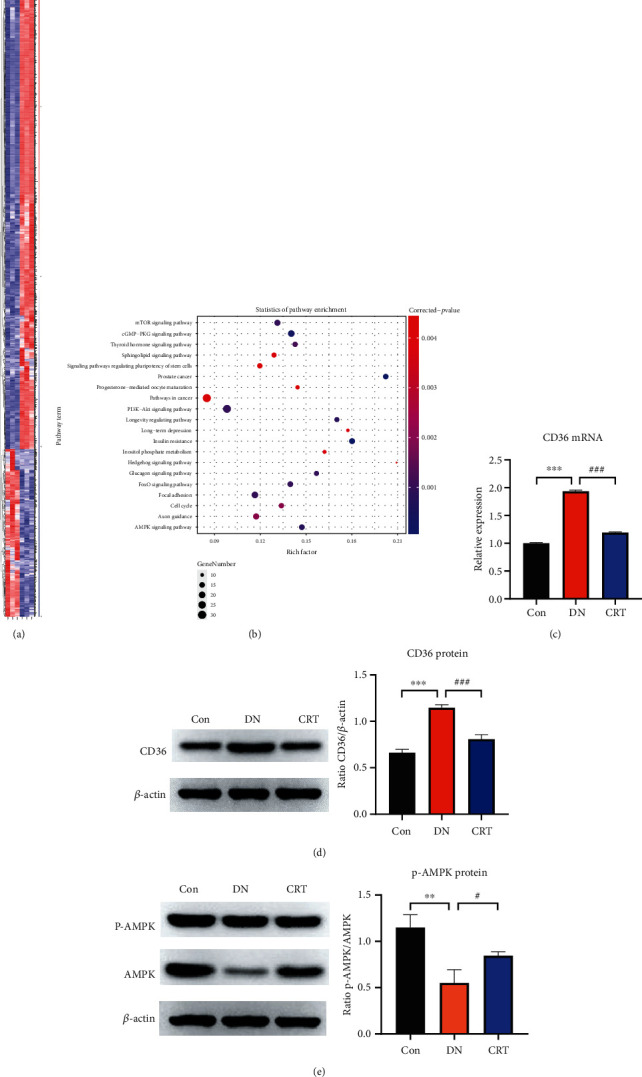
CRT regulates CD36 expression and AMPK signaling pathways. (a) Heatmap analysis of differential genes between the DN and CRT groups. (b) KEGG enrichment analysis of differentially expressed genes. (c) Real-time PCR analysis of CD36 in kidney samples of rats. (d) Western blot analysis of CD36 in kidney tissues of rats. (e) Western blot analysis of AMPK and p-AMPK in kidney tissues of rats. Data represent the mean ± SEM for 3–5 rats.  ^∗^*P* < 0.05,  ^∗∗^*P* < 0.01,  ^∗∗∗^*P* < 0.001, compared with the Con group; ^#^*P* < 0.05, ^##^*P* < 0.01, ^###^*P* < 0.001, compared with the DN group.

We analyzed possible downstream targets after CRT treatment in DN rats using transcriptome sequencing. The results affirmed that CRT effectively downregulated the expression of 1367 mRNAs and upregulated 447 mRNAs in the kidneys of DN rats. Among these, CD36 was one of the most significantly downregulated mRNAs (Figure 3(a)). Related studies have reported that CD36 is highly expressed in DN models and plays a critical role in DN progression. Furthermore, KEGG enrichment analysis of RNA-seq identified the PI3K/AKT, AMPK, and mTOR signaling pathways (Figure 3(b)). Therefore, we selected the CD36/AMPK pathway for the follow-up studies. We next verified the expression of CD36 in rat kidney tissue. qRT-PCR and western blotting showed that CD36 expression was upregulated in the DN group and significantly downregulated in the CRT group, which was consistent with the transcriptome sequencing results (Figures 3(c) and 3(d)). Western blot analysis revealed that the phosphorylation of AMPK was increased after CRT administration compared to DN rats, suggesting that CRT administration activated the AMPK pathway (Figure 3(e)).

### 3.4. CRT Had the Protective Effects on Hg-Induced HK-2 Cells

**Figure 4:**
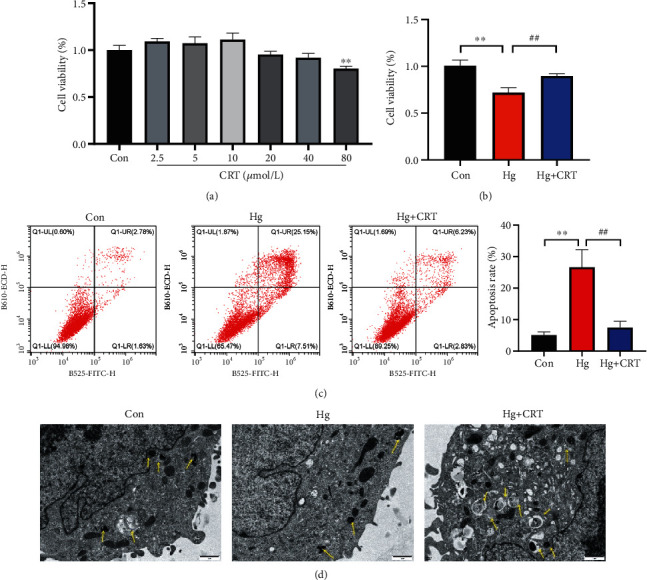
CRT promotes the proliferation and inhibits the apoptosis of Hg-induced HK-2 cells. (a) Cell viability of different drug concentrations using CCK-8 assay. (b) Cell viability of different experimental groups using CCK-8 assay. (c) Apoptotic HK-2 cells were determined using flow cytometry under conditional treatment. (d) Representative transmission electron microscopy images of autophagosomes in HK-2 cells, with yellow arrows indicate autophagosomes. Scale bar = 1 *μ*m. Results represent means ± SEM for three independent experiments.  ^∗^*P* < 0.05,  ^∗∗^*P* < 0.01,  ^∗∗∗^*P* < 0.001, compared with the Con group; ^#^*P* < 0.05, ^##^*P* < 0.01, ^###^*P* < 0.001, compared with the Hg group.

HK-2 cells were cultured with 2.5, 5, 10, 20, 40, or 80 *μ*mol/L CRT for 24 h. Then, cell viability was assessed using the CCK-8 assay. As shown in Figure 4(a), cell viability was not significantly altered after CRT (0–40 *μ*mol/L) treatment, and 10 *μ*mol/L CRT was selected for administration under Hg conditions. We then evaluated the protective effect of CRT *in vitro*. Hg markedly reduced the cell viability, which was attenuated by CRT (Figure 4(b)). Meanwhile, flow cytometry data indicated that CRT significantly reduced Hg-induced cell apoptosis (Figure 4(c)). Transmission electron microscopy revealed that CRT promoted autophagy *in vitro*, as shown by a marked increase in the number of autophagosomes after CRT administration (Figure 4(d)).

### 3.5. CRT Regulates the Expression of CD36 and AMPK Signaling Pathways in HK-2 Cells

**Figure 5:**
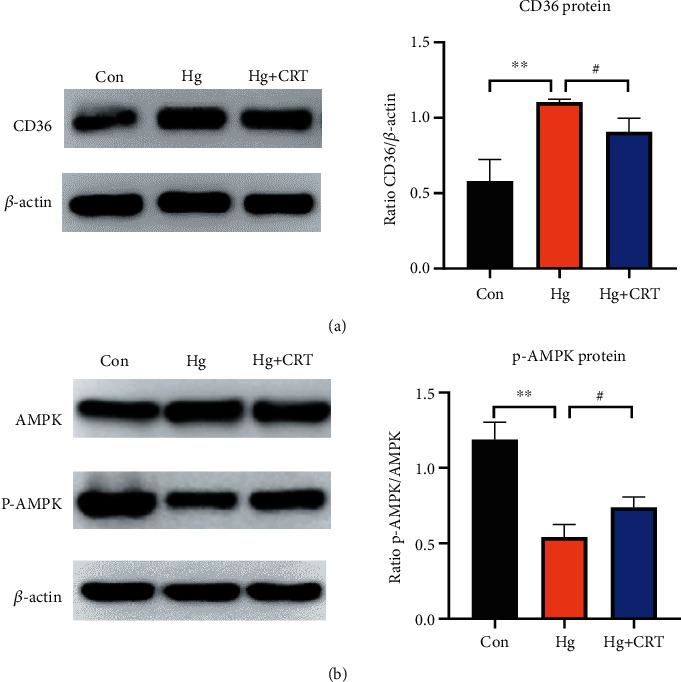
CRT regulates CD36 expression and AMPK signaling pathways in HK-2 cells. (a) Western blot analysis of CD36 in HK-2 cells. (b) Western blot analysis of AMPK and p-AMPK in HK-2 cells. Results represent means ± SEM for three independent experiments.  ^∗^*P* < 0.05,  ^∗∗^*P* < 0.01,  ^∗∗∗^*P* < 0.001, compared with the Con group; ^#^*P* < 0.05, ^##^*P* < 0.01, ^###^*P* < 0.001, compared with the Hg group.

To further verify the protective mechanism of CRT on HK-2 cells, we examined the changes in CD36 and AMPK protein expression. Western blot results indicated that CRT administration decreased CD36 protein levels, which were upregulated by Hg stimulation. Meanwhile, the protein level of p-AMPK increased after CRT treatment, indicating that CRT activated the AMPK pathway (Figure 5(b)).

### 3.6. CD36 Overexpression Reversed the Renal-Protective Effects of CRT on HK-2 Cells

**Figure 6:**
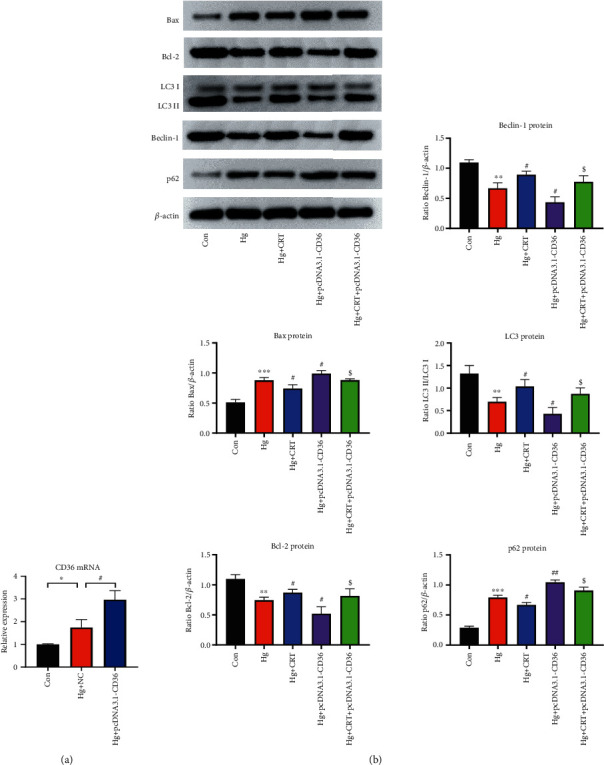
CD36 overexpression reversed the renal-protective effects of CRT on HK-2 cells. (a) Real-time PCR analysis of CD36 in HK-2 cells transfected with CD36 overexpression plasmids. (b) Western blot analysis of Bax, Bcl-2, Beclin-1, p62, and LC3 in HK-2 cells. Results represent means ± SEM for three independent experiments.  ^∗^*P* < 0.05,  ^∗∗^*P* < 0.01,  ^∗∗∗^*P* < 0.001, compared with the Con group; ^#^*P* < 0.05, ^##^*P* < 0.01, ^###^*P* < 0.001, compared with the Hg group; ^$^*P* < 0.05, ^$$^*P* < 0.01, ^$$$^*P* < 0.001, compared with the Hg+pcDNA3.1-CD36 group.

HK-2 cells were transfected with plasmids carrying CD36, and HK-2 cells consistently expressing CD36 were established (Figure 6(a)). Western blotting results confirmed that CD36 overexpression significantly increased cell apoptosis, inhibited autophagy, and reversed the renal protective effects of CRT on HK-2 cells (Figure 6(b)). In conclusion, CRT was unable to exert its cytoprotective effects under conditions of CD36 overexpression, suggesting that its function was realized through CD36.

## 4. Discussion

Diabetic nephropathy has emerged as a major microvascular complication affecting the quality of life and life expectancy of patients with DM [22]. Thus, safe and effective therapies for DN are urgently needed. Compared with western medicines targeting a single molecular target, TCM containing different components has obvious advantages and synergistic effects in the treatment of DN [22]. The CRT can considerably reduce proteinuria levels and delay renal function decline in patients with DN; therefore, it is of clinical value [11]. Li [23] showed that the clinical efficiency of CRT used to treat 70 patients with DN with massive proteinuria was 94.3%. Zhou et al. [24] showed that CRT is more effective at reducing urinary proteins and delaying DN progression than irbesartan. Through network pharmacological analysis, An et al. [12] found that the main component of CRT, regalcitonin, could improve DN through antinephritis, antirenal fibrosis, antioxidant, and podocyte-protective effects, which may elucidate the mechanism by which CRT improves DN renal function and reduces proteinuria.

Zhou et al. [9] demonstrated by High-Performance Liquid Chromatography that each CRT (0.18 g) contains 3.04 *μ*g of triptolide (C20H24O6) and 0.13 mg of epicatechin (C35H14O6). Rather than paying attention to the pharmacological actions of individual components, our study examined the renoprotective effects of CRT *in vivo* and *in vitro* and its mechanisms in providing a basis for its clinical application in the treatment of DN. Our results demonstrated that CRT administration attenuated albuminuria and renal histopathological damage in DN rats. However, CRT had no hypoglycemic effect on DN rats in our study, suggesting that CRT can alleviate DN without affecting BG levels. We revealed that CRT plays a critical role in inhibiting apoptosis and promoting autophagy in DN by reducing CD36 expression and activating the AMPK pathway. The pharmacological effects of CRT on fibrosis and inflammation have been demonstrated in related studies [13], but no studies on its autophagic properties have been found. As far as we know, this is the first study to prove that CRT promotes autophagy in DN.

Autophagy is a highly conserved process in the eukaryotic cell cycle that maintains homeostasis by degrading senescent proteins and organelles, recycling nutrients, and generating energy [25]. Basic levels of autophagy in kidney cells are essential to maintaining the homeostasis of the renal internal environment [26]. Under external stress, autophagy will be altered to adapt to the external environment, and once autophagy is dysregulated, acute and chronic nephropathy will be induced [27]. Currently, endogenous imbalances mediated by autophagic dysfunction are integral to the pathogenesis of DN, involving many signaling pathways [28]. Our study showed that CRT administration attenuated autophagy dysfunction *in vivo* and *in vitro*. Furthermore, we found that CRT inhibited apoptosis *in vivo* and *in vitro*. Apoptosis is a tightly controlled process that is critical for cell growth and development [29, 30]. Growing evidence supports the idea that tubular epithelial cell apoptosis plays a crucial role in DN pathogenesis [31, 32]. Under normal conditions, an equilibrium between autophagy and apoptosis is maintained in the body [29]. Unfortunately, DN kidney injury may be associated with decreased protective autophagy and increased apoptosis [29, 33].

To further investigate the mechanism of action of CRT in DN, we performed RNA-seq of renal tissues in different groups of rats. Transcriptome sequencing showed that among the three groups, CD36 expression was substantially altered. An increased expression of CD36 was detected in the DN group relative to the Con group. After CRT administration, CD36 expression levels decreased sharply. CD36, one of the B scavenger receptors, is involved in a wide range of pathophysiological processes and can be expressed on various cells, including adipocytes, macrophages, microvascular endothelial cells, epithelial cells (e.g., renal tubular epithelial cells), and platelets [34]. CD36 expression has been found to be upregulated in patients with chronic kidney disease, particularly diabetic nephropathy [20, 35]. Furthermore, KEGG enrichment analysis identified signaling pathways such as AMPK. The AMPK signaling pathway positively regulates autophagy-related pathways [17]. Li et al. [36] reported that CD36 inhibits the autophagic degradation of lipid droplets in hepatocytes via an AMPK-dependent pathway in nonalcoholic fatty liver disease. In our study, CRT suppressed CD36 expression and activated the AMPK pathway. Furthermore, CD36 overexpression significantly promoted autophagy and inhibited apoptosis.

In summary, we showed that the protective action of CRT *in vivo* and *in vitro* was achieved by suppressing the expression of CD36 and activating the AMPK pathway, thereby promoting autophagy and inhibiting apoptosis. CRT is an excellent drug candidate for DN treatment.

## Data Availability

The data supporting the results of this study are included in this article and its supplementary materials.

## Supplementary Materials

Supplementary Materials Supplementary materials include the primer sequences for PCR (Table 1) and heatmap analysis of differential genes between the DN and CRT groups (Figure 1 and Table 2).
