# Supplementary material for: Colquhounia Root Tablet Promotes Autophagy and Inhibits Apoptosis in Diabetic Nephropathy by Suppressing CD36 Expression In Vivo and In Vitro
Source: J Diabetes Res. 2023 Aug 16;2023:4617653. doi: 10.1155/2023/4617653 (PMC10447140; doi:10.1155/2023/4617653)
Supplement: Supplementary Materials — include the primer sequences for PCR (Table 1) and heatmap analysis of differential genes between the DN and CRT groups (Figure 1 and Table 2). [file 4617653.f1.pdf]

Table 1 The primer sequences for PCR

Gene Name	Primers Sequence (5' to 3')
Rat Cd36 F	GCAAAACGACTGCAGGTCAA
Rat Cd36 R	CCCGGTCACTTGGTTTCTGA
Human CD36 F	CAGGTCAACCTATTGGTCAAGCC
Human CD36 R	GCCTTCTCATCACCAATGGTCC

Fig. 1 Heatmap analysis of differential genes between the DN and CRT groups

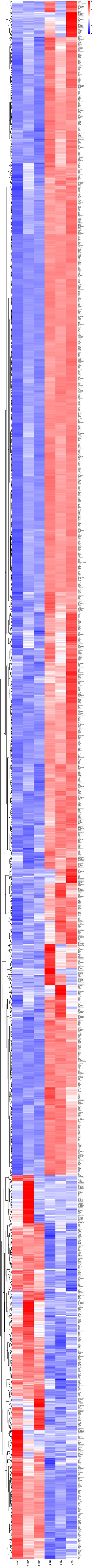
CRT−1
CRT−2
CRT−3
DN−1
DN−2
DN−3

Table 2 Heatmap analysis of differential genes between the DN and CRT groups

GeneID	CRT-1	CRT-2	CRT-3	DN-1	DN-2	DN-3
A1cf	0.7561102	0.7799323	0.7579239	1.510054	1.5281989	1.5083485
Pkhd1	0.3093695	0.3493171	0.3701307	0.9519353	1.0127604	1.0429634
Itga1	0.6271543	0.6331084	0.5349356	1.3772974	1.3507974	1.4028619
Osbpl8	0.7688011	0.8585477	0.7791445	1.5439159	1.5220364	1.5515818
Lrrc58	0.7702499	0.7007294	0.7659296	1.6095856	1.6823704	1.6166649
Mib1	0.5093989	0.5105946	0.4860531	1.2203541	1.1806881	1.1580436
Zbed6	0.2927156	0.443318	0.3589451	1.1346889	1.0585995	1.1087747
Col4a3	0.9212613	0.8801931	0.89387	1.5360676	1.5305597	1.5456785
Dmxl1	0.5465414	0.6328796	0.6109435	1.1923468	1.1840716	1.1977022
Cep350	0.3286436	0.3474233	0.3287584	0.8417787	0.8316422	0.8628433
Hipk2	0.8496439	0.8658792	0.8497076	1.4434653	1.4525007	1.4287359
Cpd	0.8194903	0.9008458	0.8542296	1.4419827	1.4425097	1.4870131
Uhmk1	0.6270104	0.6887043	0.7150927	1.3202295	1.3096301	1.3000442
Mbnl1	0.7723243	0.8430837	0.8583634	1.4538551	1.4274577	1.4595883
Lcor	0.2781639	0.3242123	0.3022028	0.7810243	0.7488595	0.766211
Zfp704	0.3831332	0.4279692	0.4573315	0.9275515	0.8975292	0.9569822
Eea1	0.6172119	0.701961	0.6406328	1.204728	1.2075077	1.2429296
Zbtb20	0.1913549	0.1857744	0.1344689	0.803026	0.7576714	0.815544
Mob1b	0.5469738	0.6242541	0.5048868	1.263301	1.238776	1.2264569
Dennd4c	0.4911127	0.5755417	0.5182385	1.0593199	1.023943	1.0586462
Secisbp2l	0.7103569	0.7495944	0.7740021	1.297407	1.2574305	1.2908917
Med13	0.5119937	0.5945157	0.5490375	1.1214127	1.0654129	1.0819141
Nbeal1	0.196098	0.23741	0.2237624	0.6726997	0.6406797	0.7177897
Lifr	0.8563268	0.8048057	0.8890632	1.4922978	1.5406188	1.4385608
Zyg11b	0.6752454	0.7197748	0.7099902	1.2185764	1.1806849	1.2416394
Slco4c1	0.5487309	0.5445363	0.5832289	1.2137147	1.2138132	1.270591
Shroom4	0.5643034	0.5822022	0.5789751	1.104117	1.1149584	1.0545489
Cd2ap	1.043032	1.192065	1.1048816	1.7262364	1.7063672	1.6939361
Myo9a	0.4953148	0.6470362	0.5282174	1.0907778	1.1342945	1.1869611
Kmt2c	0.4775802	0.4871418	0.5135291	0.9524745	0.9692179	0.9230396
Ubr1	0.3978646	0.5094825	0.5047671	1.02018	1.0216574	1.0372097
Xiap	0.7057337	0.8037366	0.7783552	1.3239689	1.2424992	1.3323039
Birc6	0.8352467	0.898302	0.859442	1.361871	1.3418207	1.3611097
Vps13a	0.6222489	0.7055128	0.6421839	1.1602516	1.1415112	1.1493903
ND4L	3.163038	3.1646938	3.1901911	3.7660423	3.6799291	3.7429068
Tnks	0.3769844	0.4587612	0.4696317	0.9763485	0.974422	1.019411
Fcho2	0.8647989	0.9641336	0.9409973	1.4569802	1.4179006	1.4396799
Nf1	0.3482444	0.4201638	0.4020917	0.8650172	0.8281411	0.8380064
Taok1	0.6458624	0.7102997	0.6541143	1.1890557	1.1675456	1.2114222
Atl3	0.7088831	0.8178451	0.7757969	1.2919576	1.2867234	1.3029378
Slc5a3	0.4102504	0.51523	0.3780256	1.2757147	1.2382017	1.0898345
Lmbrd2	0.2750264	0.3400412	0.2886003	1.0023911	0.9263963	0.960152
Fam126b	0.6490506	0.7026722	0.6555841	1.3009055	1.1996027	1.1701626
Pappa	0.1910647	0.3425669	0.2819942	0.959497	0.9642667	0.8249807
Mga	0.3301136	0.4362737	0.4381069	0.8611378	0.8383788	0.8990996
Ralgapa2	0.5357615	0.5887942	0.5679859	0.9967525	0.991155	1.0042482
Arfgef3	0.3669516	0.2303476	0.2279533	0.9359323	0.8222121	0.9556924
Zdhhc21	0.2915468	0.3244786	0.2769506	0.9422656	0.9058869	0.9901153
Macf1	0.8139077	0.8662722	0.8331836	1.3061059	1.2565318	1.2941569
Ncoa2	0.6180293	0.6562459	0.6481519	1.0685707	1.0890728	1.0744802

Ddx3	0.8623617	0.9236886	0.9458554	1.4169474	1.3464788	1.4129741
Lrp6	0.7053395	0.7756228	0.7625439	1.1971351	1.196274	1.1846934
Cpeb2	0.4201555	0.4632594	0.425148	0.8771543	0.8833263	0.8341631
Atrx	0.5549001	0.6004748	0.5939493	0.9926466	0.9639962	1.0052729
Nfib	0.4426713	0.5778881	0.4995895	1.0283057	1.0156167	1.0335192
Smg1	0.6682038	0.7606074	0.7141466	1.1566343	1.1324803	1.1598724
Ash1l	0.684733	0.7521805	0.7239806	1.1733758	1.1379934	1.1495118
Zkscan1	0.711677	0.8325905	0.7886171	1.2914424	1.2644016	1.2962374
Vcpip1	0.5266061	0.5229955	0.539903	1.0467533	0.9850848	1.0823486
Usp34	0.6089015	0.6740985	0.673269	1.1187948	1.0607957	1.086042
Mtr	0.6489659	0.5560138	0.5686339	1.1054136	1.0696232	1.1415285
Vps13c	0.5181428	0.6714849	0.5618279	1.0668868	1.0855322	1.063421
Sim1	0.548587	0.5683034	0.5386812	0.9995132	1.0078504	0.9440919
Dmxl2	0.7594219	0.838216	0.8083977	1.2378243	1.2269251	1.2224788
Pnisr	1.3253103	1.4206171	1.3875184	1.837865	1.8244784	1.8729609
Phip	0.3765615	0.5093281	0.4184383	0.9080376	0.8399243	0.903155
Itpr1	1.0883776	1.0839483	1.0580286	1.51232	1.4891151	1.5168325
Ptprd	0.8639969	0.950058	0.8910609	1.4038612	1.3782689	1.316118
Alg10	0.3803059	0.4145616	0.4369002	1.0746679	0.9825075	1.0835393
Arhgap5	0.7905112	0.9073225	0.8524001	1.288135	1.2717779	1.3581845
Rictor	0.3967854	0.5127898	0.4949558	0.881847	0.8818555	0.903938
Tmf1	0.8312061	0.9443048	0.9053476	1.3525943	1.3206134	1.3430235
Trove2	0.1974719	0.2425756	0.2242602	0.7887564	0.7590984	0.8249092
Plcb1	0.6033367	0.5933337	0.5616928	1.0399509	0.9907944	1.0216865
Rif1	0.2247587	0.3479068	0.3180244	0.7330754	0.7402943	0.8070974
Kmt2a	0.4211256	0.4967841	0.4656896	0.8245032	0.7809419	0.8367541
Lyst	0.2122928	0.2817861	0.2718684	0.6446314	0.5969405	0.6530111
Son	1.2113442	1.315391	1.3121391	1.7166608	1.7040258	1.7525445
Ar	0.157182	0.1860783	0.2273008	0.7249485	0.7519723	0.7616357
Larp4	0.5925335	0.6302164	0.6395802	1.0909738	1.1135182	1.1507126
Fnbp1l	0.923038	1.0472697	1.0297274	1.4648623	1.5018492	1.4807302
Esrrg	0.7752654	0.816948	0.8404133	1.1745578	1.2239547	1.2616927
Chm	0.4656299	0.5516509	0.5308553	1.0467252	0.9699086	1.0519559
Pik3c2a	0.5421827	0.6505605	0.6439331	1.1383435	1.0065339	1.0547012
Kat6a	0.5745912	0.6575762	0.6019098	0.9936642	0.9992961	0.986181
Clcn5	0.6364282	0.5975538	0.5702068	1.1557016	1.1559181	1.1142109
Cpeb4	1.1912763	1.2425364	1.1669938	1.7183107	1.7272165	1.6078778
Slc16a7	1.2193508	1.1378003	1.2373883	1.6182014	1.664506	1.694977
Cmtm4	0.9016302	0.976516	1.0050155	1.4399339	1.4641151	1.4863618
Pdpr	1.0561035	1.0568229	1.0957924	1.4841091	1.4953311	1.4887812
Ino80d	0.3351385	0.4250115	0.3527578	0.7390626	0.7475903	0.733183
Ttc14	0.6723926	0.7404166	0.739473	1.225326	1.141793	1.2735968
Ube3a	0.7098758	0.8201073	0.7429069	1.2142191	1.1570085	1.2082616
Hnf4g	0.6379461	0.7616008	0.6255032	1.1085149	1.2059767	1.213271
Ranbp2	1.3262286	1.4213532	1.4005092	1.8098108	1.7733671	1.8706278
Zbtb41	0.5043226	0.5487021	0.5161928	0.9465228	0.8836495	0.9014308
Thoc2	0.6580794	0.7934868	0.7162097	1.1497482	1.1130966	1.1779493
Usp9x	1.0066049	1.0555585	1.0087429	1.4477352	1.3936648	1.4334477
Pikfyve	0.7488561	0.7807201	0.7672181	1.199758	1.1418786	1.1465207
Kmt2d	0.5759349	0.6013845	0.615125	0.9415552	0.9451664	0.9451474
Mdn1	0.2525402	0.2513964	0.2528644	0.5424512	0.5143354	0.5537905

Fnip1	0.5856749	0.6546064	0.6030799	1.0505494	0.9791457	1.0055075
Acadsb	1.0714763	1.0503621	1.0770224	1.4333076	1.5320742	1.5596004
RGD130710(	0.6538013	0.7296359	0.7821103	1.1681361	1.1132438	1.1377733
Apc	0.6374143	0.6854343	0.6580046	1.0400219	1.0286287	1.0041128
Ptpn4	0.3945808	0.4957605	0.4835222	0.859689	0.8099597	0.8398185
Atp11c	0.4073114	0.4660182	0.4261	0.8348635	0.7925545	0.8450025
Klhl24	1.1178626	1.2435921	1.1489127	1.6303098	1.6152712	1.5844744
Lig4	0.6219424	0.7727472	0.720993	1.1375337	1.1217254	1.1545776
Kif1b	0.7479041	0.8274469	0.8505314	1.1822312	1.2233641	1.2149441
Lats1	0.704941	0.793251	0.7638559	1.1330755	1.1262918	1.1731085
Hook1	0.960035	1.032171	0.9556601	1.4202738	1.3745423	1.3882044
Pank3	0.8439727	0.9752214	0.9091186	1.3351469	1.3745135	1.3130355
Zfp192	0.167355	0.1996583	0.2043025	0.5412045	0.5196577	0.5467242
Fgd4	0.2789341	0.3269621	0.3255749	0.8034001	0.7970071	0.7620074
Aff4	0.7643899	0.8592952	0.854417	1.2545558	1.189948	1.2308327
Kdm7a	0.3804741	0.4506176	0.3792978	0.7948492	0.7553899	0.7605415
Bmpr2	0.8133587	0.9182061	0.881295	1.3262522	1.2499025	1.2756573
Klhl11	0.3514774	0.4088582	0.2895592	0.9629012	0.9846372	0.9802066
Tmem245	0.7148066	0.7890784	0.7480983	1.1259192	1.1245422	1.1066537
Megf9	0.6402633	0.6133116	0.5837201	1.1231263	1.0705043	1.0610967
LOC1025515	0.2613871	0.2855672	0.3045956	0.8268114	0.7739767	0.8248104
Fam135a	0.5613071	0.6215223	0.6082508	0.9859662	0.9363971	1.0208868
Zfc3h1	0.6627831	0.7548351	0.6992621	1.0997788	1.0603854	1.082822
Lpp	0.7360984	0.8468877	0.8161835	1.1889273	1.2044757	1.1763576
Scai	0.1145228	0.179866	0.1563262	0.5750023	0.5462085	0.620146
Insr	0.6976437	0.7490388	0.7512291	1.0986234	1.1587524	1.1047986
Fras1	0.3771672	0.4733392	0.4431826	0.7848438	0.8387983	0.7588324
Clock	0.7172009	0.7993159	0.740947	1.1819333	1.1132272	1.1309222
LOC1025465	0.8458157	0.9892086	0.939395	1.3872473	1.3595392	1.3193532
Xpr1	0.8869787	0.8563497	0.8657951	1.3165584	1.2666273	1.305777
Zfp292	0.3469918	0.4903826	0.4588611	0.8338364	0.8145668	0.8573905
Pcm1	0.8222499	0.8796772	0.8667975	1.2253099	1.2335333	1.2238474
Ankrd10	0.9491519	0.9479066	0.929593	1.306766	1.304877	1.3812059
Epm2aip1	0.8242252	0.8538891	0.8844319	1.2308231	1.1965663	1.3041695
Bdp1	0.371889	0.4747761	0.4279778	0.7916699	0.7859345	0.8182042
Nufip2	0.6277111	0.7815132	0.6905098	1.1583688	1.0832504	1.1303633
Tulp4	0.4251697	0.4254893	0.4345495	0.808407	0.7481696	0.825051
Clmn	1.0515559	1.0949144	1.0946011	1.4521008	1.4630231	1.4691151
Fndc3b	0.8415857	0.901748	0.8497757	1.2533708	1.2201272	1.234002
Tead1	0.8814997	0.9463633	0.9367361	1.2796414	1.2684626	1.306817
Tnpo1	0.869364	0.9528735	0.9351526	1.3185805	1.2810136	1.2901299
Nfat5	0.9032958	1.0540116	1.0251597	1.4229341	1.4018715	1.4226423
Jmjd1c	0.7721993	0.8379963	0.8196447	1.1752326	1.1985868	1.1656718
Braf	0.3982961	0.4634259	0.4397816	0.7726941	0.7679306	0.7777817
Slc8a1	0.7712574	0.8036295	0.7421558	1.1545249	1.1881356	1.1123365
Dync1h1	0.950063	1.013821	1.024163	1.3517509	1.3560479	1.3808683
Gc	1.1972615	1.1298615	1.1496787	0.5525095	0.6156914	0.578547
Edem3	0.7692747	0.8482032	0.7931847	1.2394068	1.1614356	1.1653335
Akap9	0.6219275	0.7206603	0.669069	1.0197719	1.028614	1.0319149
Fam208a	0.6394592	0.7799046	0.728143	1.0997863	1.100158	1.1047451
Asxl2	0.5388199	0.5623502	0.4977232	0.917442	0.8578443	0.8853014

Baz2b	0.5678646	0.6866492	0.6175077	0.9815542	0.9914108	0.9890849
Atf2	0.8353304	0.8618411	0.8823931	1.301069	1.3082323	1.3018997
Gstp1	2.6551209	2.574793	2.5038534	2.0874864	1.8679992	2.124194
Ankrd12	0.426746	0.4988792	0.4650633	0.8205273	0.8161442	0.7738269
Chd9	0.3359277	0.3946692	0.3658666	0.7440531	0.6317205	0.7204207
Rev3l	0.3866734	0.4390749	0.4483874	0.7654585	0.714885	0.7686491
Fat1	0.9325009	0.9585072	0.968994	1.2755954	1.2878303	1.3277726
Bmpr1a	0.9731478	1.0541933	1.0167791	1.367796	1.3640247	1.3975937
Xrn1	0.5287662	0.5990331	0.5690156	0.9260231	0.8730441	0.922527
Utrn	0.630683	0.7617506	0.7352701	1.0709621	1.0650494	1.1319834
Ralgapa1	0.7401827	0.8413354	0.7599083	1.1672014	1.1367637	1.1361298
Nipbl	0.7617258	0.8251245	0.8018189	1.1578888	1.1230771	1.1503362
Clk4	1.098508	1.1939994	1.1873253	1.5582717	1.5116056	1.5568176
Itgb8	1.11517	1.2214105	1.1253157	1.6180799	1.5127241	1.5628321
Tjp1	0.8438052	0.9454748	0.9006329	1.2938595	1.231469	1.2900995
Efr3a	0.8070884	0.9427523	0.9410562	1.3013336	1.2766984	1.3038433
Ccdc50	0.8728877	1.0136676	0.948151	1.3987874	1.3045046	1.3605843
Phf20l1	0.9032633	1.0126932	1.0060795	1.3493561	1.3290859	1.3941532
Tcp11l1	0.3327582	0.3828897	0.2920692	0.879538	0.7872171	0.8066407
Atm	0.3982752	0.4625915	0.407418	0.7632653	0.6934573	0.7840265
RGD1561161	0.6188974	0.6087749	0.5767555	0.955189	0.9330065	1.0091014
Trpm7	1.0066831	1.0984018	1.0845183	1.4716228	1.3987365	1.4444685
Ccdc141	0.9366322	0.9023332	0.9118411	1.3524634	1.2392068	1.3143208
Usp25	0.6234405	0.6719426	0.6114016	0.9724896	0.9801857	1.0193909
Atxn1	0.2611113	0.3147365	0.2119072	0.738994	0.7589783	0.7557028
Gnl3l	0.9826491	1.0091995	1.0884379	1.4261717	1.424286	1.4766155
Iqgap2	1.1501606	1.2836152	1.2054387	1.6554349	1.5926779	1.5901356
Rc3h1	0.5700606	0.6711929	0.6410454	1.0219467	0.9594412	0.9532033
Ptar1	0.2324949	0.2784616	0.2384242	0.8544082	0.919349	0.8945107
Camsap2	0.5994638	0.6485135	0.6411848	0.9723778	0.9374229	0.9596019
RGD130570[illegible]	0.440905	0.4709855	0.4382627	0.8857421	0.837348	0.8766566
RGD130593[illegible]	0.6556539	0.7794692	0.73811	1.1129601	1.0661712	1.1210198
Cpsf6	0.6237672	0.7293154	0.6524828	1.0138852	0.9890647	1.0283965
Map3k2	0.2208793	0.1230308	0.1176597	0.8042321	0.7643823	0.7544241
Srek1	0.9053465	0.9832215	0.9751435	1.3353356	1.2886235	1.3196151
Lrp2	2.0830474	2.0730397	2.0459155	2.4522949	2.4580414	2.4383214
Dcaf17	0.7561855	0.8311373	0.7740316	1.1821318	1.139672	1.1698163
Erbin	1.204733	1.3504579	1.2575987	1.6838247	1.6144101	1.7054471
Slc30a1	1.1612641	1.1584762	1.1577196	1.4675925	1.5401576	1.5778934
Tbc1d8b	0.5994875	0.6950829	0.6651834	1.0139826	0.9895537	1.0253746
Avl9	0.7895386	0.8235769	0.8243785	1.1606929	1.1166943	1.1525733
Prkaa2	1.2045002	1.2273004	1.1697731	1.6161762	1.5701177	1.5404423
Man1a2	0.8160254	0.8426564	0.8338351	1.191362	1.1747821	1.191154
Kitlg	0.8167305	0.9030336	0.8312134	1.2453206	1.1545116	1.2440552
Usf3	0.5463274	0.5823876	0.5766199	0.915241	0.8453002	0.8692577
N4bp2	0.4066528	0.4905433	0.4551621	0.7465296	0.7625105	0.8176507
Tet2	0.4519276	0.5031393	0.5159263	0.7795903	0.7996094	0.8297178
Soga1	0.2061372	0.2294298	0.2214502	0.6302357	0.657597	0.7272664
Pdzd2	0.6144201	0.6609771	0.6130563	0.931738	0.9358296	0.9770078
Hipk3	0.9928519	1.0791487	1.0463994	1.4099733	1.4058988	1.3704749
Erbb4	0.1995957	0.2366748	0.2809716	0.6363825	0.7775451	0.8278564

Zc3h12c	0.4079382	0.3919761	0.3904442	0.9366454	0.8195299	0.7722337
C1galt1	1.1974993	1.3075512	1.2172914	1.6485019	1.6087689	1.6012946
Zfp266	0.6269973	0.7247924	0.7440877	1.0104394	1.0504984	1.1047615
Rock2	0.8831679	1.0140403	0.9440916	1.3358584	1.2969969	1.308919
Uba6	0.5499864	0.677722	0.6488873	0.9706681	0.9680815	1.0179192
Myef2	0.8247786	0.9044507	0.8660591	1.234076	1.2348382	1.2391718
Fam179b	0.5680851	0.6533422	0.6143519	0.9917549	0.9174664	0.9749865
Rps6ka3	0.8403988	0.875845	0.8564579	1.1769004	1.1635653	1.1860234
Crebrf	0.9612565	1.0250602	0.9976706	1.3714386	1.3422343	1.3132834
Hectd1	1.028915	1.1195374	1.0804833	1.4287802	1.4082328	1.4209734
Amn	2.3811855	2.2179225	2.2736188	1.8726932	1.9016699	1.867327
Magt1	1.0530216	1.1309622	1.1382108	1.4901507	1.5450671	1.5489935
Itsn2	0.9180784	0.923054	0.9567207	1.2687145	1.2454209	1.2902702
Abcb7	0.8714614	0.8726329	0.8603705	1.2576579	1.2566811	1.2584623
Dync2h1	0.3727634	0.4244468	0.4219181	0.6666459	0.6871863	0.7153183
Cacnb4	0.4677961	0.4599584	0.4186153	0.8403648	0.7247552	0.8064384
Abl2	0.3254762	0.3411851	0.3477632	0.7073482	0.6529109	0.6866372
Ric1	0.6600155	0.7172729	0.6705763	1.0261616	1.0191774	0.9881988
Zfp654	0.5784979	0.6677482	0.6355092	1.0139769	0.9657853	0.9750228
Akap11	0.9594817	1.0061872	0.9973242	1.3141828	1.2959264	1.3256732
RGD1309995	1.0176438	1.1220613	1.0589186	1.4943378	1.3688008	1.4738987
Kdm5a	0.7958756	0.8579827	0.8238847	1.148554	1.1239501	1.1592577
Mtpn	1.4072628	1.4966498	1.4924761	1.8639877	1.8133263	1.8202238
Aplp1	1.5399447	1.5140992	1.5593481	1.1818643	1.2237491	1.2191494
Ahctf1	0.7694374	0.8431923	0.8408423	1.1269607	1.1337429	1.1781706
Lin7c	1.0015732	1.0582982	1.1129886	1.5748907	1.652058	1.6285978
Wdfy3	1.0772404	1.076283	1.0829619	1.4237781	1.4058346	1.4020819
Phc3	0.5386194	0.6586615	0.6067721	0.9071759	0.9034586	0.9707653
Rbm25	0.8809676	0.9988407	0.9431323	1.2809524	1.2617074	1.3318047
Polr2e	2.2065426	2.1926638	2.2188982	1.8331565	1.9026825	1.8450364
Gcc2	0.8036069	0.9003549	0.8643822	1.188444	1.1629068	1.2157813
Sema3d	0.1933235	0.2818897	0.2800459	0.6566107	0.7673996	0.7079176
Ubr3	0.916567	0.9677367	0.9112141	1.2676711	1.2459724	1.2564096
Arpc4	1.7506047	1.8034403	1.7983819	1.4364984	1.4720994	1.4854317
Tp53inp1	1.0344297	1.1755882	1.1977998	1.5053915	1.6134814	1.5697238
Wnk1	1.4133604	1.5300528	1.4091136	1.8402894	1.7985801	1.8527671
Bclaf1	1.1216389	1.2239299	1.2154008	1.5253302	1.5267919	1.5517034
Atp7a	0.3720209	0.4381942	0.3498687	0.7328196	0.7186255	0.7660943
Prkdc	0.3612414	0.4051663	0.3926918	0.7053918	0.6170794	0.7073052
Dgkh	0.2344495	0.2558126	0.1888322	0.5500991	0.458472	0.5287036
Ftl1	3.7318381	3.6631122	3.6188945	3.2903446	3.3170528	3.2634649
Islr	1.490935	1.4021342	1.5085486	1.0807758	1.0855986	1.1479576
Ubn2	0.5166624	0.5307714	0.4734355	0.8092095	0.7755669	0.8255216
Tgfbr3	0.7511315	0.8317853	0.7984088	1.1040382	1.1652081	1.1736628
Ccdc88a	0.4448082	0.455357	0.4634654	0.7726634	0.7008966	0.8646343
Arid2	0.5838068	0.6415182	0.5942987	0.9317751	0.9129192	0.9011987
Sp3	0.5390775	0.7232697	0.5878151	1.029054	1.0300961	1.0784293
Btbd7	0.8145347	0.886338	0.8462137	1.1992205	1.1610304	1.1678791
Rapgef6	0.2943367	0.3751286	0.3870921	0.7192755	0.7308063	0.7683442
Ogt	1.2380021	1.383863	1.3433853	1.7086288	1.6701615	1.6934004
RGD1307235	0.7437359	0.8002979	0.7700168	1.0893029	1.0685768	1.1127335

Spag9	1.1441129	1.1846209	1.1683329	1.5138646	1.4738787	1.4923433
Rpl14	2.5803931	2.6385598	2.595557	2.2868094	2.2892987	2.2534157
Kif21a	0.9197338	0.9527547	0.9310982	1.2735908	1.2713338	1.2412687
Tmem72	1.0337187	1.0572393	1.1514413	1.3888491	1.5023312	1.5457787
Plxna2	0.491724	0.5606048	0.515407	0.8035338	0.8073636	0.8279403
Prpf4b	0.9361667	1.0493814	1.0054562	1.3674954	1.3186466	1.3430867
Fbxl17	0.9657043	1.0035709	1.0158324	1.3522919	1.3449527	1.3601425
Ep300	0.7643179	0.8273279	0.8135585	1.096701	1.1185476	1.1254224
Spta1	0.7084157	0.5507671	0.6357662	1.0194374	1.0834026	1.0062453
Nr2c2	0.5857446	0.6899429	0.6527891	1.0716523	1.062432	1.1094373
Luc7l3	1.3040623	1.3873639	1.3345592	1.7075007	1.6594262	1.7022629
Ptch1	0.5690115	0.5996059	0.6344862	0.9188966	1.0517664	0.9851088
Vps13d	0.748699	0.7757561	0.7600489	1.0520362	1.0584332	1.0587623
RGD156514:	0.1993383	0.135198	0.1797525	0.6853516	0.6779555	0.6141986
Trps1	0.6139271	0.7934202	0.6776766	1.0433198	1.0505382	1.0906354
Zscan26	0.7810267	0.9000427	0.8712025	1.179246	1.1659388	1.2067967
Ago3	0.1951005	0.2118772	0.1753913	0.5302609	0.5240762	0.5185204
Lrba	0.9168221	1.0041061	1.0033	1.285668	1.3181798	1.3046801
Dach1	0.6616617	0.7259486	0.6977586	1.0871156	0.9651598	1.0497217
Rb1cc1	0.7432739	0.8164556	0.818329	1.1062632	1.0785348	1.1249952
Kif5b	1.1632986	1.3232434	1.2463409	1.6246815	1.590924	1.6178515
Ipo7	1.0604763	1.2142228	1.1137832	1.512991	1.4970377	1.4804395
Cyp2d1	1.9508665	1.8946337	1.9653414	1.290222	1.4279473	1.603349
Cygb	1.622853	1.6589038	1.6338915	1.3493633	1.2654841	1.3321534
Ptprb	0.735725	0.814141	0.8146312	1.1353408	1.0784618	1.102754
Sdsl	1.2249401	1.2956521	1.1703253	0.7030796	0.742971	0.7470419
Agt	1.6422732	1.5048818	1.6375134	1.1713843	1.2176938	1.2754598
Synj1	0.6505816	0.6795948	0.6925279	1.0188363	0.9601072	1.000041
Prrg4	0.7211982	0.5805418	0.688427	1.3128066	1.1788101	1.1332756
Agps	0.4908816	0.5678891	0.532288	1.014577	0.987931	1.1012445
Il12rb2	0.4469693	0.5506618	0.5116082	0.8380412	0.8949452	0.9172109
Ppp1r9a	0.2914575	0.2950057	0.3154828	0.5914012	0.5998695	0.6185442
Cd99	2.131078	2.0677179	2.1297271	1.7040721	1.8090211	1.7537512
Ahnak	0.9202018	1.081955	1.0015363	1.3368301	1.3313679	1.4096584
Zfp462	0.3215713	0.3250641	0.374764	0.6245694	0.6229803	0.6186821
Emilin1	1.3013042	1.284626	1.2283068	0.9674656	0.93153	0.9903517
Peg3	0.5188891	0.5779562	0.5459877	0.8105804	0.8388904	0.8776
Tmed5	0.7003826	0.9099595	0.8289561	1.3349614	1.2498609	1.2868927
Rsbn1	0.4423481	0.5463236	0.4977645	0.8498135	0.8245762	0.8620345
Zeb2	0.506117	0.629496	0.5539496	0.9383637	0.8439873	0.9611369
Ccnt2	0.6253679	0.7209237	0.7169522	1.0134246	0.9805925	1.0131311
LOC10369O(	0.4412194	0.6555447	0.575673	1.108072	1.0472246	1.1676814
Map1lc3a	2.0205924	1.9326012	1.9465857	1.624171	1.6178894	1.6529387
Eef1g	2.4936138	2.567897	2.5178477	2.1515351	2.2411041	2.1762941
Myo5a	0.7677989	0.7512055	0.7003683	1.1305718	1.0092748	1.1300334
Mfap3l	1.4463231	1.5078115	1.5363879	1.8281908	1.8128875	1.836899
Slk	0.9333246	1.0453309	1.0125697	1.3075282	1.3238718	1.351279
Hcfc1	0.3549701	0.3696403	0.4002858	0.6813153	0.6551909	0.6732065
Rbm25l1	0.6062707	0.6736118	0.6501832	1.047529	0.984528	1.0413926
Akap13	0.7325352	0.7991571	0.7708633	1.077884	1.0584143	1.0638209
Ids	0.7706223	0.9213433	0.8884724	1.255617	1.1921693	1.2260497

Phldb2	0.9393392	1.0346983	1.1070236	1.3713002	1.3861368	1.4202767
Kras	1.0180474	1.1227186	1.0492471	1.4192292	1.3746203	1.396707
Pcf11	0.4744733	0.5469349	0.5654596	0.8335074	0.7966839	0.8320837
Slc5a8	1.1737624	1.305968	1.173313	1.7234079	1.8588375	1.5750649
Fryl	0.4426004	0.5084445	0.4709855	0.7535699	0.7273315	0.7387288
Cdc14b	0.5606176	0.632405	0.6179691	0.9789139	0.9797526	1.0380619
Kansl1l	0.5957324	0.6595138	0.6616605	0.971592	0.9095799	1.0539283
Orc4	0.7507401	0.8160071	0.8442397	1.1499941	1.1125468	1.1727169
Zfyve16	0.5814147	0.6779184	0.637231	0.9410119	0.9307586	0.9636561
Pbrm1	0.7121088	0.8139415	0.7605896	1.0827088	1.0496439	1.0737656
Lgr4	1.2278119	1.2741241	1.3144823	1.5886004	1.6194751	1.5993994
Jmy	0.5681888	0.5592919	0.5556751	0.8445843	0.8116224	0.8227281
Pign	1.2604068	1.1861023	1.2515527	1.5930618	1.5355956	1.5938659
Suco	0.8366844	0.9367866	0.9025068	1.1959147	1.1972962	1.2182285
Stag2	1.0366435	1.1600857	1.0986946	1.4269365	1.3981114	1.4786596
Acsl4	0.8784307	0.9976715	0.9714352	1.348529	1.3573184	1.2439318
Herc1	0.7465743	0.7515651	0.7854769	1.0279943	1.0386086	1.0705949
Herc2	0.626533	0.6713485	0.6461599	0.9711578	0.9090754	0.9271087
Atp13a3	0.4001782	0.2977076	0.316973	1.0675452	1.0828187	1.0217851
Syne2	1.0430085	1.0892291	1.0304338	1.398978	1.3194585	1.4084854
Sox6	0.3159157	0.3809435	0.3719312	0.6334481	0.6241578	0.625418
Col4a5	0.9208105	0.9891285	0.9820188	1.3165897	1.2525967	1.279632
Clic5	1.0782544	1.1383739	1.078204	1.4404153	1.4169652	1.3906186
Dock4	0.2627079	0.3024536	0.2883392	0.6081694	0.6233155	0.7252467
NEWGENE_15	0.5077923	0.5184307	0.4822036	0.8628407	0.8995168	0.8893243
Mysm1	0.5195805	0.6608847	0.5618678	0.9061317	0.8878727	0.9474225
Usp45	0.3112912	0.4136116	0.3043936	0.7733711	0.6855685	0.77892
Uprt	0.2191104	0.3053767	0.2879466	0.783834	0.7760581	0.7571047
Dapk3	1.7484106	1.6509499	1.6606407	1.3475619	1.3640494	1.3690583
Nup153	0.848961	0.9132008	0.869399	1.2063529	1.1720167	1.1602639
Pfkfb2	0.8714768	0.8595502	0.8822728	1.1865411	1.1867698	1.1495456
Ankrd26	0.296395	0.3139138	0.2888496	0.6933068	0.6395187	0.6661066
Zdhhc17	0.6357309	0.7318591	0.7224289	1.0555989	1.0231341	1.052991
Gan	0.2619162	0.2784515	0.2548658	0.6276953	0.6419394	0.5968315
Ppp2r3a	0.855176	0.9318019	0.9533473	1.2120903	1.2049616	1.2502469
Igip	0.5376744	0.5869363	0.5504475	0.8666808	0.8091104	0.825957
Pde3b	0.6960018	0.7158911	0.7511339	1.1219369	1.1263952	0.9951616
Sesn3	1.1794867	1.1819516	1.1728336	1.574562	1.4314146	1.5444106
LOC100360(	1.5890218	1.503562	1.4545518	1.1124638	1.0768757	1.0920151
Tnrc6b	0.592114	0.6658811	0.6392132	0.9237776	0.930544	0.8913496
Pdzd8	0.6316964	0.7051461	0.630267	0.9365047	0.9456638	0.9672299
Bod1l1	0.4842408	0.5172783	0.4940795	0.7635065	0.7702509	0.7624455
Prpf40a	0.961831	1.0745726	1.0283953	1.3465629	1.3093969	1.3706684
Diaph2	0.1675263	0.1765313	0.2066653	0.5225272	0.5385161	0.5669404
Cdk6	0.3103696	0.3927898	0.3169311	0.7067214	0.7485725	0.7068743
ND5	3.2751185	3.245556	3.3124455	3.6798894	3.6054978	3.6072627
Wsb1	1.2961185	1.3425085	1.2981395	1.7701651	1.5818666	1.7014906
Ndufb10	2.3651737	2.3128699	2.2745228	1.9633087	2.0265823	1.9580552
Qser1	0.5515178	0.6440444	0.5938611	0.9587899	0.9038535	0.8728005
Rsf1	0.4931273	0.5553178	0.4665989	0.805181	0.7566347	0.7952858
Sgms2	0.9769694	0.9564403	0.962813	1.3463095	1.2532446	1.3347951

Wwp1	1.4707835	1.5566813	1.4643601	1.8185023	1.792304	1.8485171
Palm	2.0477277	1.9188335	1.990195	1.6449036	1.6693963	1.6500693
Pdpk1	1.0092833	1.0701756	1.0825881	1.3516902	1.3593961	1.3748484
Zfp445	0.7870703	0.8829764	0.8364484	1.1077195	1.1142409	1.1893155
Eif3f	2.0466557	2.0650052	2.0913882	1.7717361	1.7847207	1.7772807
Trip11	0.8226355	0.8608619	0.8131758	1.1351452	1.0990363	1.1345172
Rfx7	0.4148482	0.4950633	0.4269941	0.7764368	0.7191984	0.7516261
Ptbp3	1.0338354	1.1930725	1.1046929	1.4660613	1.4133459	1.4749954
Zfp281	0.3607547	0.4572541	0.4106342	0.8430151	0.8545672	0.8636645
Hmbox1	0.944648	0.9450898	0.9446684	1.2309001	1.2490284	1.2345765
Stam2	0.8384051	0.9409799	0.9433077	1.2882292	1.2870487	1.2960699
Mybl1	0.555739	0.6883709	0.598295	1.0255586	0.8805826	1.0849239
Lcorl	0.2554603	0.3688483	0.3033018	0.6929746	0.6511479	0.678228
Ascc3	0.5690037	0.6654706	0.5886814	0.9322807	0.8660998	0.9261682
Atp2b4	0.744563	0.761446	0.7527566	1.0668664	0.9776569	1.0984152
Reps2	0.7977746	0.8578635	0.7594642	1.2133708	1.0871532	1.1229905
Slc13a1	1.9706266	2.0576758	2.1217096	2.4042573	2.4331094	2.6735977
Mycbp2	0.7656815	0.8362258	0.8296386	1.1102725	1.0849719	1.1044362
Ildr2	0.4592582	0.4251161	0.4414798	0.7875922	0.7501767	0.8876409
Brwd1	0.5678216	0.66006	0.6558198	0.9043327	0.8917507	0.9313519
Itch	1.2008361	1.3000827	1.2650464	1.5947044	1.519271	1.6133339
Piga	0.3040839	0.4455839	0.4013539	0.870655	0.8719403	0.7891504
Arhgef12	1.1721099	1.1899873	1.2129821	1.5149541	1.4858874	1.4834473
Mecp2	0.6040599	0.6988569	0.6627574	0.9170899	0.9290226	0.9510064
Tmem33	1.165016	1.2733552	1.1743033	1.5696947	1.5028449	1.5389648
Rpl18	2.5537552	2.4856388	2.5003619	2.1684841	2.2343248	2.1943101
Nr3c1	0.9619533	1.072523	0.9992298	1.3435331	1.3071795	1.3294205
Mdm4	0.9775823	1.080278	1.0358605	1.3627702	1.3117656	1.3583697
Ythdf3	0.9665369	1.0931694	1.0309967	1.3539404	1.3225692	1.3759325
Ppdpf	2.0748286	2.090676	2.1031203	1.7332463	1.8184901	1.8062471
S100a16	1.7911711	1.8826946	1.7833315	1.4751705	1.5359796	1.519795
Creb3l2	0.7621978	0.8415413	0.7965714	1.0898707	1.1104768	1.181976
Tgfbr1	0.6847673	0.8667245	0.8002527	1.1581111	1.0753434	1.1763874
Crim1	1.0838598	1.2359246	1.1839335	1.4791655	1.5028926	1.5136957
Npat	0.3660086	0.4909026	0.4518977	0.7588113	0.7286222	0.804847
Ggt1	3.001954	3.0305649	3.0100616	2.7075649	2.6985339	2.7607792
LOC102548[illegible]	0.3835737	0.4798716	0.5073638	0.8236962	0.8107486	0.8408564
Cdkl5	0.1890043	0.1512453	0.2291719	0.5891516	0.6264869	0.7481874
Far1	0.8020563	0.917139	0.8462585	1.1978581	1.1691067	1.2612651
Mgat5	0.585774	0.6304259	0.602038	1.045034	0.9587414	1.0017316
Dst	1.1435353	1.1908172	1.1944867	1.4488087	1.5003803	1.489999
Gsk3b	1.1697737	1.231523	1.1961293	1.5121559	1.4736028	1.5183542
Slc9a3r1	2.80926	2.7811	2.7746001	2.4625587	2.5127624	2.4679209
Usp33	1.0051199	1.1215568	1.0610428	1.4049887	1.339672	1.3860781
Chd6	0.5765641	0.6032147	0.6229466	0.8559982	0.8395815	0.8772693
Itgav	0.9813678	1.1079643	1.1131715	1.4177304	1.4252596	1.3702577
Slc10a5	0.4646374	0.5246934	0.5887269	1.0625375	1.0668792	1.1129179
Slf2	0.4279014	0.5369015	0.4830694	0.8182241	0.7873426	0.7959799
Bbx	0.1432193	0.2101111	0.2114855	0.6464695	0.5828656	0.6600356
Cdc42bpa	0.7201403	0.7889602	0.7409776	1.0155916	1.0219788	1.0631207
Ap1g1	1.1385395	1.2266447	1.2102124	1.4777699	1.5107769	1.5134022

Rbm26	0.8230537	0.9263285	0.8859073	1.1704613	1.156888	1.2063011
Acvr1c	0.2443424	0.2475135	0.2510195	0.7564932	0.7721819	0.7530549
Ptov1	1.98475	1.9324248	1.9475839	1.6608607	1.6727109	1.6326187
Fam91a1	1.3778628	1.55644	1.5176417	1.8666004	1.8253217	1.8293058
Rock1	0.8241563	0.9952298	0.9178087	1.2624558	1.2216876	1.257792
Dlg1	0.9759743	1.0165466	0.9738534	1.327251	1.266128	1.2977909
Fndc3a	0.8391464	0.9234895	0.8541277	1.1733918	1.141067	1.1765693
Usp31	0.1091802	0.1758059	0.1480151	0.5364918	0.5178639	0.4902898
Exosc5	1.915013	1.8480267	1.9242789	1.5606665	1.6138864	1.5995989
Col12a1	0.7175834	0.7291738	0.732633	1.0495405	0.9433447	1.1052218
Slc25a36	1.3562931	1.3761151	1.4100761	1.7207988	1.6427813	1.7117435
Prpf39	0.7523945	0.9061888	0.9103117	1.2106595	1.1886691	1.2624768
Zfp148	0.7288105	0.7934879	0.7576864	1.0469497	1.0273264	1.0296165
Slc35a3	1.1352256	1.1388548	1.080937	1.4129446	1.4551046	1.4690814
Otud4	0.8883277	0.9855936	0.9211027	1.2361401	1.2103842	1.2341784
Gnb2	2.1254819	2.077974	2.1012124	1.7882018	1.8251238	1.8225718
Zbtb44	0.5870172	0.5963275	0.5693388	0.8867866	0.8386207	0.8705111
Loxl1	1.4852769	1.4830097	1.4692113	1.2266651	1.0534888	1.1292997
Snx13	0.9947201	1.0866553	1.0572341	1.3828289	1.3469959	1.338257
C5	1.5436806	1.4970876	1.5807541	1.9025706	1.8036653	1.9272458
Slc4a4	1.940176	1.9018281	1.9262571	2.3501762	2.2531106	2.2253412
Mbd3	2.129838	2.029555	2.1015573	1.7246557	1.8085294	1.7401205
Klf12	0.4094036	0.481131	0.5172337	0.7837411	0.7939636	0.7263516
Fam199x	0.0516141	0.0573542	0.1042114	0.6864263	0.6197952	0.6109499
Flnb	0.7793459	0.879678	0.861679	1.1638675	1.1778772	1.1103467
Fam208b	0.5450004	0.6057567	0.5749645	0.8319976	0.8210075	0.8947953
Ankib1	0.8517124	0.934883	0.8936315	1.1901021	1.1756104	1.1769559
Bptf	0.586358	0.6811808	0.6358092	0.8964123	0.9073445	0.9008129
Gxylt1	0.5638521	0.6573607	0.6094102	0.912416	0.8853594	0.9050626
Tbl1xr1	0.9405982	0.9924019	0.8923747	1.3010041	1.2489131	1.2334979
Skil	0.8189006	0.8036493	0.7101693	1.1961568	1.0226327	1.2097368
Asb13	1.2528374	1.2114568	1.2974061	1.5880032	1.5969141	1.5506826
LOC100361(	0.2126201	0.215474	0.1789472	0.6171271	0.5679668	0.6028542
Pcmtd2	0.9129455	1.0082062	1.0087348	1.3022926	1.2736149	1.2656455
Amer1	0.1453462	0.2015856	0.1369989	0.5487294	0.5759166	0.5844108
Zfp26	0.2087566	0.3132666	0.2955581	0.6005643	0.5981388	0.6427533
RGD130907(	0.8528163	0.8953601	0.8711023	1.1698555	1.1320478	1.1861449
Uevld	0.1631044	0.1544964	0.1142226	0.6254218	0.6298531	0.7019671
Ireb2	0.9707581	1.1115137	1.0681934	1.3799138	1.3382113	1.3873071
Mbtd1	0.7495691	0.812941	0.8034445	1.1741565	1.0722338	1.0837812
Rcn3	1.2954044	1.3123433	1.2727478	0.8997454	0.9207769	0.9412979
Strn	0.7054607	0.820854	0.7546484	1.1168496	1.0460086	1.0507853
Cers6	0.4960467	0.4917883	0.4891338	0.9312246	1.0024255	0.9123597
Arhgap29	1.0140756	1.1324537	1.1275344	1.390568	1.3814618	1.4447353
Syne1	0.8292849	0.9031378	0.8521871	1.1896557	1.1013501	1.1781652
Usp32	0.7867121	0.8917195	0.8567382	1.1489146	1.1064146	1.1409354
Spcs3	0.9718977	1.2237596	1.1061244	1.5995281	1.5615938	1.604106
Deptor	1.0296324	1.1609731	1.0831179	1.3718224	1.4005798	1.4208273
Spopl	0.812757	0.8821839	0.8250639	1.1567794	1.1020298	1.179133
Serping1	1.7661532	1.8666576	1.898781	1.5203116	1.4090832	1.5829121
Slc5a12	1.6782366	1.6367244	1.7082368	2.1618126	2.1268313	1.9423556

LOC103691(	0.6446155	0.7184629	0.66303	1.0062398	1.025523	1.0964538
Igsf9b	0.2616246	0.2267806	0.2335297	0.5687797	0.634339	0.6654594
Zfp397	0.3998822	0.5525682	0.4805924	0.8647833	0.8793508	0.9602042
Chd1	0.5334303	0.693636	0.6156233	0.9690733	0.9115448	0.9319098
Nat9	1.4373231	1.4674527	1.3876279	1.153425	1.1425324	1.1051386
Efcab14	1.0354036	1.1227427	1.0932288	1.397312	1.3600308	1.3754463
Rasa2	0.4877713	0.4082187	0.4304255	0.9967148	0.9354235	0.8341808
Cdk12	0.6334694	0.6968054	0.6272565	0.9241791	0.9319978	0.9679513
Rnf152	1.3364553	1.4153357	1.370584	1.7185942	1.6858547	1.6448736
Dopey1	0.5769091	0.6096479	0.603366	0.8825414	0.8507588	0.8592038
Pde4d	0.7729053	0.8075609	0.7638132	1.2334749	1.1359199	1.0337997
Med14	0.5522415	0.6568802	0.6516749	0.9393184	0.9017794	0.9099923
Lysmd3	0.2509302	0.3521698	0.3601095	0.8981479	0.88524	0.9728107
Kat2b	0.7688516	0.8893319	0.884732	1.1467815	1.1410884	1.1884757
Brwd3	0.1296054	0.187395	0.1438222	0.4391169	0.3740518	0.4258427
Chuk	1.2610543	1.3247533	1.3376501	1.639328	1.6255384	1.5876835
Dhx36	0.7469983	0.8735483	0.8532582	1.1608724	1.0857579	1.1731449
Ppp1r12a	0.7827619	0.8884035	0.8721385	1.1627816	1.0923487	1.1758885
Zfp518a	0.2663306	0.4285004	0.3494022	0.6760868	0.6796697	0.7973694
Epn1	2.2316469	2.1672445	2.1947965	1.873958	1.9390762	1.8852404
Hspbp1	1.669006	1.6210823	1.6435842	1.2938923	1.3940813	1.3207654
Trim44	1.0137758	1.1061242	1.0607241	1.4614003	1.4637913	1.437289
Smarcad1	0.5612719	0.6883398	0.628994	0.9577453	0.8863447	0.9860132
Rnpepl1	1.7314772	1.6521838	1.661533	1.3651474	1.4164804	1.3723123
LOC689130	1.9354956	2.0487421	2.0361194	1.6432045	1.7544459	1.6591321
Zfx	0.597322	0.6652878	0.6148698	0.9262946	0.8836893	0.909745
Smc2	0.3538062	0.4539516	0.3587385	0.7782325	0.6885315	0.7725945
NEWGENE_15	0.53027	0.6297222	0.6302026	0.8796157	0.8737184	0.9162958
Btaf1	0.5593242	0.6911697	0.622699	0.9182859	0.8840571	0.9313551
Nrip1	0.7035364	0.7150511	0.6605612	1.0027389	0.9021363	1.0309069
Aldoa	2.9851823	2.9531967	2.9684294	2.6196606	2.7191465	2.6129588
Rbm27	0.4998947	0.6019232	0.5815142	0.9175018	0.8893588	0.8897788
Exoc5	0.9747077	1.0568415	0.9371098	1.3483656	1.299587	1.302661
Rabgap1l	0.6060311	0.7713581	0.6417825	1.0008021	0.97291	0.9893154
Mast4	0.0123734	0.0210824	0.0120904	0.1359497	0.1637417	0.1412835
Shprh	0.4405326	0.4807765	0.4703284	0.7578036	0.720956	0.7493631
Tmtc3	0.3798542	0.4684722	0.420111	0.7924965	0.7110221	0.738973
Cpeb3	0.570244	0.5949618	0.5991975	0.8884871	0.9095345	0.8171796
Arpc1b	1.981023	2.0515166	1.9522942	1.7246226	1.7163819	1.6871057
Gfpt1	1.0089613	1.1410913	1.0688805	1.3428704	1.4073982	1.3924917
Prkaa1	0.6483412	0.7386523	0.7527845	1.0267712	0.9892061	1.0213145
Ppp1r1a	2.2275907	2.126882	2.2073181	1.7517916	1.8459106	1.9431202
Pls1	1.3410748	1.4352451	1.3595077	1.6653562	1.6381573	1.7880045
Zmym2	0.6968837	0.7826274	0.7568607	1.0387195	0.9728013	1.0440718
Cdc27	0.7190341	0.827369	0.829093	1.1054774	1.0727051	1.0881051
Cdh6	0.5831954	0.5157366	0.5610089	0.9809833	0.9930059	0.8551489
Lad1	1.9564137	1.8648172	1.8833063	1.5926203	1.6124392	1.63318
Lrrk2	0.6924286	0.8549762	0.7935842	1.0859446	1.0713298	1.0897852
Masl1	0.0872495	0.1162762	0.053234	0.4786429	0.4565373	0.5003035
Atox1	2.066114	1.9933985	2.0062583	1.6269084	1.7467125	1.7008482
Hcfc1r1	1.8706017	1.8603322	1.877251	1.5434431	1.6035463	1.5069238

LOC103690(	1.3731102	1.160845	1.2622037	1.6553061	1.6150986	1.7133582
Gcat	1.7875874	1.6818642	1.7290357	1.3884548	1.4729902	1.3751559
Paxbp1	0.8190471	0.8504752	0.8333425	1.1653537	1.0875534	1.1284283
Park7	2.5245528	2.4085707	2.4471603	2.1627901	2.1608677	2.1630592
Polr3k	1.5603968	1.6203446	1.6520751	1.2902754	1.3198139	1.3086729
Raph1	0.2818608	0.3570114	0.2972284	0.6116873	0.6228537	0.6142446
Fem1b	0.678974	0.7058217	0.6316494	1.0771315	1.0762243	1.0509053
Cyp2d2	2.3601927	2.5245122	2.4407692	1.9423333	1.8478275	2.1776738
Ndufs7	2.3819513	2.2461363	2.3235957	1.9633111	2.0451007	1.9538255
Dennd5b	0.6090063	0.6056284	0.6167568	0.9603592	0.9377611	0.8931272
Mob3b	0.560542	0.5029936	0.4831356	0.8275677	0.9736018	0.9649558
Foxo1	0.7068491	0.7687668	0.7610088	1.0936895	1.0719414	1.0977482
Rasal2	0.232829	0.2300664	0.1939516	0.5617951	0.5547958	0.5395626
Dab2	1.9458041	1.8932915	1.8838602	2.2429836	2.1671914	2.2455333
Taf1	0.5778043	0.7035997	0.6430063	0.9118886	0.9146736	0.9428293
Sema3c	0.5859174	0.7543003	0.6949015	1.0169754	0.9542735	1.0419963
7-Mar	1.1142871	1.1955424	1.2436615	1.4924726	1.4788115	1.4866915
RGD131159	0.5039759	0.492147	0.5041801	0.773341	0.7583752	0.7283818
Cers4	0.3421164	0.3511099	0.3099068	0.7123355	0.7419061	0.7592883
Smcr8	0.8532722	0.8427782	0.8170922	1.156401	1.1191319	1.1270146
Emc10	1.9363157	1.9187279	1.9333311	1.6084525	1.6937266	1.5957244
Cdc34	1.7302237	1.7019267	1.7182093	1.4175573	1.4614814	1.4026595
Smchd1	0.4439443	0.6152983	0.5183635	0.8268597	0.8203145	0.8605721
Pura	0.4921468	0.5183105	0.5239657	0.7644436	0.7333227	0.7487362
Pclo	0.0721581	0.0736576	0.0885667	0.2613389	0.2363473	0.2946308
Tsc22d2	0.6805862	0.6522204	0.6304333	0.9875182	0.9291089	0.9157663
Ndufb7	2.271344	2.1815364	2.1905655	1.8971739	1.9513349	1.8728288
Greb1	0.4241734	0.4782846	0.4014923	0.7565768	0.7593941	0.6842518
Ttc39b	0.6207457	0.6222421	0.554726	0.9075673	0.9134077	0.9582277
Fat4	0.024853	0.0307516	0.0364698	0.1525177	0.1499385	0.1971088
FAM120C	0.4313005	0.4691277	0.4935522	0.7124645	0.6794809	0.7298895
Lamc3	1.0131855	0.9119649	0.9957898	0.5733152	0.6872962	0.7299308
Trio	0.6680553	0.7322296	0.7134461	0.9810724	1.0124188	0.9237706
Col6a2	1.2123783	1.3405709	1.246941	1.034172	0.9260557	0.9739727
Dzip3	0.1583991	0.2849525	0.2883001	0.608178	0.6390058	0.625214
Foxn2	0.8424074	0.9799128	0.8836524	1.244484	1.2581181	1.2006225
Usp37	0.4336953	0.5508776	0.4664827	0.7952224	0.7312827	0.7880795
Tra2a	0.9520703	1.1551242	1.0434604	1.4265372	1.3586416	1.3870275
Arid5b	0.4680574	0.5274452	0.5649732	0.8706344	0.9326546	0.8270599
Eml5	0.1696183	0.1830215	0.2179048	0.4707824	0.4881495	0.5199119
Aldh6a1	2.2838303	2.2122551	2.3251446	2.6028521	2.5928538	2.5779546
Mtmr9	0.5618804	0.6498408	0.6395563	0.9949924	1.0200129	1.0380933
Pi4k2b	0.9619129	1.0242791	1.0633169	1.3414604	1.3492023	1.3410955
Orc3	0.6328645	0.681136	0.725542	0.9885969	0.9533842	1.0621766
Phlpp2	0.42697	0.4821865	0.4781025	0.8187513	0.8340857	0.7707707
Eps15	0.9960994	1.0668783	0.9775644	1.3156765	1.2597606	1.338731
LOC679818	0.6758968	0.3730496	0.5982421	1.027618	1.0128037	1.12697
Sntb2	0.3884873	0.3595662	0.3710913	0.8639858	0.9158129	0.8123628
Hmg20b	1.6767809	1.5575995	1.6181489	1.2847353	1.3574426	1.3091734
Atxn7	0.6285407	0.7233583	0.6470036	0.9407774	0.9105822	0.9577301
Mpdz	0.5466408	0.6560626	0.5334111	0.8410918	0.8909492	0.8726529

Bri3	2.1349386	2.0280642	2.0801182	1.7682146	1.8221071	1.7628453
Vps50	0.690977	0.7295537	0.7436705	1.0342149	1.032995	1.0471285
Vps13b	0.4475614	0.5966559	0.535503	0.8426657	0.801066	0.7665805
Pgls	1.4156721	1.2744789	1.3346659	0.962259	1.0749807	0.9782577
Ago2	0.3475129	0.4283769	0.3655882	0.8474262	0.8276078	0.7669025
Setbp1	0.2328787	0.3240309	0.2854693	0.5383204	0.4982431	0.5694472
Tet3	0.3559086	0.3388113	0.357184	0.5655419	0.5686167	0.5838183
Ppargc1a	0.977306	1.0624126	1.1182902	1.3733859	1.3867313	1.3219499
Fmn1	0.0636337	0.0628045	0.0612745	0.2514341	0.297583	0.2883474
Npr3	0.8185545	0.8945477	0.7988444	1.1658511	1.0739096	1.1351119
Wdr19	0.648337	0.660644	0.6544091	0.9180871	0.9118421	1.0064885
Dis3	0.6019151	0.7098862	0.6613806	0.9626159	0.9671631	1.0036678
Elovl7	1.0907853	1.2396729	1.2294949	1.5383555	1.5826678	1.5631085
Amdhd2	1.6282383	1.5073866	1.6107347	1.3153171	1.2626304	1.2876847
Zfp771	1.6995209	1.5441364	1.6513942	1.307082	1.3608292	1.2707488
Ninj1	2.2776768	2.0769466	2.1601111	1.8525441	1.8564954	1.8150184
Fbxo30	0.643186	0.6709403	0.6636807	0.9709866	0.8992943	0.9973203
Ccdc124	1.7015888	1.6441939	1.6298306	1.3302858	1.4163695	1.3677224
Casp8ap2	0.2901508	0.3588709	0.3105947	0.5968176	0.6018488	0.6364063
LOC103690(	0.0077895	0.0065452	0.0113662	0.0728633	0.2345049	0.227771
Xpnpep3	0.6067409	0.702241	0.65093	0.9845082	0.954868	0.9775236
RGD1309621	0.6113804	0.7248067	0.7582935	1.033425	0.9515969	0.9879158
Neurl3	1.3610186	1.4389258	1.3497299	1.0223691	1.1289088	1.0508898
Bag3	1.5998251	1.4653234	1.4696014	1.2258182	1.2028872	1.234098
Trim33	0.5675811	0.6549541	0.5935875	0.8964522	0.8071529	0.8887079
Rfx3	0.2466088	0.3123974	0.2926911	0.5828516	0.5464474	0.5646629
Rnf13	0.8208007	0.8527535	0.9192827	1.1822764	1.1901128	1.2492863
Mospd2	0.8541848	0.9609844	0.8793286	1.2074434	1.1432982	1.2389732
Mcoln3	0.9750248	1.1508851	1.092264	1.4949031	1.7451037	1.3862111
Ypel2	0.7016839	0.7518307	0.5644701	1.1775058	1.0172227	0.9923837
Lrrc40	0.7270338	0.8144516	0.7596302	1.0423153	1.0298246	1.104286
Slc39a9	1.0881575	1.1296491	1.1654321	1.424173	1.429718	1.4177804
Ate1	0.4162216	0.4093744	0.4420314	0.745261	0.7650379	0.787047
Nbea	0.4272869	0.5167899	0.4832888	0.6980392	0.6825655	0.7884889
Fbxo31	1.5396575	1.5196715	1.5459299	1.230206	1.3222986	1.2060579
Cnot6l	0.6169741	0.7957396	0.7240851	0.9732827	1.0356926	1.0264646
Parp14	0.5555908	0.6352015	0.565931	0.9023173	0.782185	0.8958261
Dmtf1	0.8163519	0.7964745	0.7830678	1.083669	1.0465998	1.0997493
Shank2	0.2578693	0.2497232	0.2860608	0.458703	0.5728878	0.5368091
Spata9	0.5075478	0.5768767	0.5806049	0.9903239	0.9872882	1.0201505
Babam1	1.8487776	1.6863749	1.7848414	1.3847854	1.5230638	1.4165993
Cltb	1.8064769	1.7621639	1.7807132	1.4416841	1.5614605	1.4584251
LOC1009124	0.2155634	0.2521178	0.2660723	0.5417113	0.6201467	0.6249451
Usp53	0.5452106	0.5810587	0.6267598	0.8148517	0.8709328	0.8746504
Zswim6	0.4047414	0.4191404	0.4501861	0.7265816	0.7467817	0.7775673
Pik3cb	0.7274573	0.8136802	0.81357	1.0739939	1.071104	1.0310595
Nipa1	0.6185187	0.6973593	0.6585255	1.0418982	0.9792198	1.0319203
Cep85l	0.0807939	0.1199974	0.0775061	0.355018	0.3776531	0.433028
Ppip5k2	0.4431756	0.5029243	0.398319	0.8895651	0.9253746	0.9006854
Lsm4	1.6054279	1.5390516	1.5349583	1.1836306	1.2828688	1.1647588
Abi2	0.7307124	0.8214596	0.8015456	1.1050856	1.0313379	1.0739996

Arl13b	0.3610254	0.4043212	0.4424846	0.6748145	0.7325531	0.8208142
Mcrip1	1.5767076	1.4802153	1.5782392	1.2256346	1.2723123	1.2568735
Hhip	0.2742789	0.2467706	0.1976072	0.76275	0.792596	0.554764
Shoc2	0.8530442	0.9488234	0.913518	1.1984676	1.2207198	1.2219379
LOC1001348	2.0868667	2.219465	2.3179687	1.924911	1.7799276	1.9117334
Lrrcc1	0.792749	0.8664722	0.9278995	1.2055308	1.1181537	1.1647417
Hba2	2.3831667	2.5067178	2.566934	2.2088227	1.8559254	1.9834015
Arid4b	0.0460947	0.0581089	0.0404671	0.3944566	0.2048952	0.2931254
Pdxdc1	0.8082103	0.8412907	0.7530165	1.0283924	1.1021403	1.1319258
Hook3	0.5433039	0.5815749	0.581867	0.9836359	0.8503731	0.9751251
Ptbp2	0.5835124	0.6350646	0.6195308	0.9100462	0.9216543	0.9591018
Sf3a2	1.3215921	1.2865558	1.3690324	1.0420055	1.039995	1.0446147
Crybg3	0.2071592	0.3314463	0.2866936	0.5770905	0.5714448	0.5119872
Ints6	0.7071286	0.8166396	0.6910824	1.0643205	0.9926769	1.024917
Socs4	0.3397112	0.4157512	0.3446803	0.7897763	0.804284	0.825334
Cldn20	0.0112177	0	0.005515	0.1621919	0.2172878	0.1946953
Rab5al1	0.8851952	0.9124241	0.8723083	1.1795169	1.1730501	1.2317635
LOC1025495	0.5747192	0.571595	0.5767879	0.9433778	0.9458923	0.9737403
Esco1	0.7543636	0.8316416	0.7650889	1.1055334	1.0181252	1.0605709
LOC1036909	0.0430577	0.1110949	0.080488	0.5568969	0.5240257	0.5552251
Smc4	0.6743868	0.8411152	0.7410435	1.0459334	1.0460644	1.0670753
Fam76b	0.6049992	0.6789663	0.7247121	1.0274827	0.9904697	1.0273604
Ints2	0.3121089	0.3863057	0.3566769	0.6842349	0.6302972	0.6502521
Fth1	3.7767729	3.8954411	3.8842852	3.5119447	3.6309624	3.5249582
Tmem207	0.7621456	0.9900712	0.8907455	1.1746458	1.4513811	1.389535
Cldn8	1.2710253	1.433047	1.4218635	1.62773	1.7327067	1.7070778
Oxr1	0.982675	1.2014709	1.1668799	1.416518	1.4588341	1.4673923
Ttbk2	0.2387385	0.3148881	0.2731505	0.5600457	0.5312023	0.5370798
Qprt	1.9571271	1.830173	1.988313	1.6160252	1.6634034	1.5944014
Rasa1	0.739006	0.8652959	0.7902038	1.1047659	1.0727361	1.1100428
Sacs	0.0751557	0.0866533	0.0775729	0.2607102	0.2574985	0.2268008
Creb1	0.480417	0.620045	0.5334491	0.8554174	0.8103917	0.8501265
Morc3	0.6329486	0.8178431	0.7562005	1.0082514	1.0407992	1.0966192
Kcnj10	0.6760619	0.5989524	0.5807623	0.9649363	0.971455	1.0577643
Cd28	0.5192655	0.5805927	0.4720525	1.0207201	0.7943457	0.9778537
Zfp317	0.5707642	0.6199914	0.6117456	0.8566271	0.8876418	0.9589514
Ap4e1	0.335912	0.4305329	0.4038766	0.7427288	0.7352583	0.787741
Pard3b	0.4913197	0.4403907	0.4708474	0.8391526	0.803654	0.7979878
Rab11fip2	0.4283348	0.4664951	0.4664611	0.8822294	0.9023452	0.9298876
Ythdc2	0.3041871	0.4060584	0.417163	0.6656876	0.6508432	0.6796662
Senp7	0.4440958	0.5171224	0.4866041	0.7699062	0.7643172	0.804563
Ocel1	1.4692846	1.301413	1.3449252	1.0350737	1.0664119	1.0245576
Timm13	2.0510983	1.9087642	1.9970066	1.6832431	1.7259648	1.581568
Adm2	1.8089792	1.6204687	1.7109512	1.3195543	1.2807283	1.4765454
Atr	0.3263241	0.365841	0.3701203	0.5961319	0.5783581	0.6349881
Slc22a22	1.355016	1.4373021	1.4552078	1.741723	1.6140923	1.8330144
Lhpp	1.9112031	1.8553297	1.8734545	1.5270485	1.6716442	1.5765484
Mpv17l2	1.7317472	1.6557237	1.6403727	1.3729381	1.4125284	1.4007972
Acp5	1.9917381	1.8106169	1.8853204	1.5237675	1.6166889	1.6313274
Rrm2b	0.699567	0.819096	0.7683412	1.1816263	1.1579001	1.2392608
Ikzf2	0.0725359	0.1645731	0.1266111	0.3977551	0.3732129	0.4493828

Ikbkg	0.5332807	0.5722489	0.5688564	0.8191693	0.7750019	0.8561213
Gtf2a1	0.6044234	0.7059825	0.7105513	1.0033908	0.9801282	0.9829832
Hspa4l	0.493801	0.5597305	0.5709676	0.8688617	0.7160424	0.8701702
Kdm5d	0.5745107	0.7019594	0.6693415	0.9119166	0.8831512	0.9479941
Mxra7	1.2977459	1.2717513	1.3732087	1.0079884	1.0195813	1.0714036
Cav2	0.8945906	1.1253536	1.0180259	1.3549702	1.3178862	1.3515874
Arid4a	0.6070913	0.7290598	0.6180196	0.9352283	0.9424216	0.9194531
Carf	0.3575603	0.4385929	0.3923343	0.7301307	0.7476225	0.6664522
Fgd6	0.1148659	0.1424483	0.1125187	0.3777672	0.3562012	0.4130425
Thsd7a	0.1843602	0.2803866	0.2015999	0.5087102	0.5330517	0.4791174
Scrn2	1.7702485	1.7573322	1.6618244	1.2941851	1.4644543	1.5008664
Tma7	1.3034122	0.1129168	1.1844575	0.0521434	0.0132859	0.0716254
Pcdh17	0.4644378	0.5955254	0.6413813	1.0219785	0.7783064	0.9321916
Ccdc15	0.0164477	0.0206013	0.0372454	0.2111552	0.1893874	0.191115
Ccdc85b	1.453696	1.3417727	1.4146577	1.0299924	1.0385947	1.0612075
Bola1	1.6231039	1.5312212	1.608313	1.1777395	1.308895	1.2406569
LOC679894	0.1857221	0.2357213	0.2410876	0.4677793	0.3810303	0.4690759
Sectm1b	0.9370753	1.0185166	1.0449394	0.5500413	0.635318	0.4925288
Robo2	0.3536165	0.3832264	0.3646241	0.6271958	0.6184976	0.5537045
Trim23	0.7115573	0.8087729	0.7871744	1.0371762	1.0268174	1.0721057
Smc5	0.4731476	0.57909	0.5083506	0.7994804	0.7613902	0.8245171
Gng7	1.4741627	1.1434078	1.4828242	1.007821	0.7680578	0.987145
F5	0.5986672	0.673152	0.8013741	0.9371692	1.0400106	1.0361456
Mier3	0.4217973	0.5991273	0.5113464	0.8362001	0.7784388	0.8589422
Yod1	0.3011971	0.2678813	0.3837773	0.8365602	0.9416025	0.8403375
Atad2b	0.101065	0.1237758	0.1772135	0.4735763	0.4860536	0.5565424
Srgap3	0.3661327	0.4481364	0.3776396	0.6478835	0.7864216	0.7976595
Gchfr	2.107364	2.1235908	2.0652728	1.7491222	1.9043322	1.7312575
Bach1	0.4375964	0.5291866	0.493226	0.8553268	0.822258	0.8235568
Apoh	0.8461115	1.2523637	1.0653095	0.1792173	0.5221777	0.2082867
Psd3	0.2237478	0.2515315	0.2015954	0.4758015	0.407182	0.4597312
Tgfb1	1.3412467	1.3899395	1.2205771	1.0190466	0.9811243	1.0108771
Pgap1	0.2850406	0.2846241	0.2786781	0.654118	0.5951793	0.6572531
Zbtb6	0.5351057	0.6544828	0.5908466	0.8828337	0.8870353	0.9375916
LOC680520	0.5456545	0.4855986	0.4971856	0.804892	0.8176375	0.7533227
Dpy19l4	0.4215493	0.5339826	0.4938702	0.806705	0.7835813	0.8162462
Cidec	1.6916392	1.8279931	1.78159	1.3250739	1.5643472	1.3980095
Wrn	0.4665915	0.5021562	0.445914	0.7357726	0.6515734	0.7372036
Ccdc39	0.5085047	0.4884336	0.5212102	0.7831618	0.7717958	0.8892171
Greb1l	0.146951	0.1358644	0.1459318	0.404718	0.3718742	0.3843262
Rps28	2.0758321	2.0683123	2.009401	1.7629625	1.8136033	1.6915705
Bag4	0.5967613	0.6897676	0.7392411	0.9890965	1.1214058	1.0302789
Sp4	0.1956425	0.2083476	0.2372697	0.5043558	0.5330615	0.6394259
LOC100910:	1.6550229	1.6846398	1.5398363	1.9033357	1.9703549	1.9465292
Zfp236	0.386635	0.5038348	0.4906993	0.6890599	0.7201432	0.69835
Zmat1	0.5171656	0.5701963	0.5814084	0.8034377	0.7806652	0.8448851
Hspa13	0.6060524	0.7680639	0.7305597	1.1070117	1.0607846	1.1364877
Alms1	0.3410998	0.4117146	0.407803	0.5848667	0.6037588	0.6122709
Ecm1	1.3612362	1.2763153	1.2448446	1.0150692	0.8303906	1.0482185
Manea	0.5455079	0.7211071	0.6478482	0.9952751	0.8988793	1.0041295
Pias1	0.7569901	0.9044565	0.8900206	1.1868725	1.1453359	1.1632912

Kifc1	0.8832447	0.8295616	0.880274	0.3521292	0.5056332	0.235022
LOC103690[illegible]	0.4021902	0.5006142	0.4615926	0.6909168	0.6592987	0.7411807
Trappc6a	2.051005	1.8813993	1.9750443	1.6104084	1.7454504	1.566723
Scand1	2.2620232	2.0581829	2.1611685	1.8015005	1.9222663	1.782112
Scn1b	1.8407031	1.5033633	1.7148284	1.1975712	1.3179119	1.395785
Nhsl2	0.128975	0.1901996	0.1715943	0.3879166	0.3959768	0.3329818
Dip2b	0.4673603	0.5341189	0.4745914	0.7206513	0.7443988	0.7516022
Gprin3	0.1675865	0.2935495	0.289495	0.4820198	0.6538737	0.5908322
Tmem238	1.3473121	1.2177347	1.2994646	0.9955823	1.038505	1.0005506
Plce1	0.3539979	0.3933981	0.3930576	0.6266032	0.567391	0.5904834
Hspb1	1.4821452	1.6103627	1.4580989	1.2360012	1.1385282	1.2316281
RGD156024[illegible]	0.6030767	0.7353616	0.6101824	0.957321	0.8877719	0.9225065
Tmem160	1.542288	1.4417934	1.4552273	1.1211476	1.1771606	1.1485398
Gnaq	0.7547682	0.9294818	0.8011618	1.2183059	1.2678704	1.2559266
Abca5	0.1950713	0.2376883	0.2163348	0.4824363	0.463176	0.4811167
Folh1	1.1145245	0.9706316	1.0367628	1.3991394	1.275517	1.3385241
Smoc2	0.9553242	0.9733021	0.8701525	0.5810449	0.5563592	0.692857
Klhl3	0.6486468	0.5764827	0.5974054	0.9728644	1.016323	1.0037693
Tagln	2.0534138	2.1776002	1.8996244	1.7515393	1.6865201	1.7882328
Guca2a	1.3273816	1.2540195	1.2742337	0.8080952	0.8276915	0.8194494
Zbtb16	0.9306426	1.0887257	0.9542395	0.7532447	0.780766	0.6689065
Cd74	2.8839183	2.8741526	2.5882405	2.4389188	2.3986104	2.5091131
Nup58	0.7045734	0.7590291	0.766137	1.0741378	1.0905807	1.0127366
Rbm41	0.0873266	0.1571653	0.1752986	0.4820014	0.4722035	0.5324148
Ckb	2.4727083	2.2880159	2.4080234	1.9792037	2.1713025	2.0746542
G2e3	0.1722117	0.2077438	0.1880633	0.4139186	0.4373765	0.4739026
Bmpr1b	0.5438494	0.574467	0.5984223	0.8349893	0.8671103	0.8871141
Slc25a29	1.2439693	1.0694411	1.2472342	0.8722705	0.8537154	0.9104008
Hba-a1	1.3042231	1.4023396	1.1776048	0.7539339	0.7627098	0.7565366
Mcur1	0.9702836	1.0110649	1.0170307	1.3016223	1.3767954	1.3549746
Pggt1b	0.613701	0.732988	0.7222913	0.9813546	0.9537071	1.0504843
Plagl1	0.188128	0.229619	0.3109122	0.5011674	0.5733073	0.6547579
Jrkl	0.3820579	0.517325	0.4565356	0.8047675	0.7882593	0.83388
Pomc	1.007742	0.9264026	1.0033834	0.4947095	0.5354779	0.5204051
Arap2	0.3084926	0.3895257	0.3210577	0.6536601	0.5492917	0.6107062
Zim1	0.0673978	0.0651041	0.1049338	0.297042	0.3408458	0.3271607
Tlk1	0.2393644	0.2468524	0.292831	0.6217431	0.5369406	0.5634977
Mapk1ip1l	0.6298972	0.6684452	0.566722	0.9005734	0.9224185	0.9813781
Phactr2	0.494124	0.5916134	0.5603598	0.8422814	0.7608131	0.8396776
Apoe	2.9650354	2.8136643	2.8523304	2.6746837	2.4885892	2.4888977
Glis3	0.7473858	0.805655	0.8072111	1.1336444	1.1880067	1.1471653
LOC102555[illegible]	1.9042716	2.2409949	2.1709479	1.7518055	1.8198991	1.8009695
Cep290	0.1633928	0.2942809	0.2471666	0.52102	0.4598199	0.5485997
LOC501038	0.8858668	0.9873209	0.9588621	1.2378525	1.2739441	1.2832379
Nup155	0.5249714	0.6137204	0.5473938	0.8035951	0.8340189	0.86637
Dennd4a	0.3088811	0.3373972	0.2956632	0.588517	0.5169494	0.5528117
P2rx4	1.4257306	1.56898	1.587454	1.3351657	1.0695112	1.1311095
Trpv3	0.1802057	0.1573378	0.1674018	0.4491189	0.4089408	0.4084406
Hprt1	0.2040437	0.150571	0.1057401	1.1504722	1.146575	0.2068519
Tmod2	0.3877271	0.3359872	0.1729321	0.7570113	0.6747039	0.7392923
Cebpb	1.7817716	1.6341675	1.5983441	1.3909854	1.4244954	1.2559002

Kdm6a	0.056787	0.1020874	0.1027659	0.3265323	0.3378923	0.3927859
Gna13	0.7159993	0.8371646	0.8013791	1.2117651	1.1507395	1.1661199
LOC1025483	0.5812974	0.6193284	0.6190145	0.8630328	0.9082338	0.9065477
Abcc8	0.5552115	0.578695	0.5061276	0.1171623	0.2769158	0.2205222
LOC1083529	0.0566062	0.1369817	0.0771017	0.4201865	0.3829632	0.3584365
Sds	1.1401946	1.3493439	1.2567555	0.7910864	0.9702077	0.8446025
Zbtb34	0.1176021	0.1352347	0.1177285	0.4308124	0.4330949	0.3986839
Rnpc3	0.4720844	0.5356335	0.4509437	0.8477635	0.8015114	0.7943544
Lnpep	0.0639658	0.0609738	0.0669607	0.3564347	0.2637371	0.1849029
Asl	2.2720389	2.0611364	2.1364478	1.9168639	1.8726215	1.8476547
Hecw2	0.229633	0.2358841	0.2218846	0.4528467	0.4228684	0.473277
Gpm6a	1.0741585	1.1502597	0.9560501	1.4552527	1.2318471	1.4912967
Hnrnph3	0.3749793	0.4053026	0.3963403	0.656098	0.7597703	0.8068256
Arhgef38	0.1776438	0.1334704	0.1636828	0.5329264	0.4864097	0.4808815
Nhs	0.4334972	0.5571015	0.5406465	0.8652428	0.8344059	0.6641252
LOC1003635	0.3245385	1.8845136	0.0938473	0.0330726	0	0.0189027
LOC1003652	0.5042267	0.3990237	0.3926761	0.7359945	0.7559041	0.8245341
Gabpb2	0.3141193	0.3468	0.3196571	0.6326063	0.5978393	0.645274
Atp10b	0.0939703	0.1419535	0.1044922	0.3113694	0.3227695	0.405048
Arsk	0.6648142	0.7108008	0.7465648	1.0121722	1.0883493	1.0819953
Pde7a	0.3932007	0.5125817	0.4796698	0.7128361	0.7368976	0.7066605
Flrt2	0.3955859	0.5163107	0.4806338	0.8285559	0.6762105	0.741141
Tmcc3	0.4769933	0.6094722	0.5485582	0.8690711	0.789695	0.7656194
Cldn3	1.2773686	1.134368	1.3110942	0.7205305	0.9453942	0.9250525
Znrf3	0.1818817	0.1964605	0.2874967	0.5523958	0.6218249	0.6104292
Exph5	0.1359712	0.18941	0.1981295	0.3608	0.4296991	0.3958035
Rad54l2	0.3526605	0.3790582	0.377491	0.598368	0.5968589	0.6474828
Orai1	1.4673609	1.3297002	1.4521863	1.0495683	1.1428198	1.1697715
LOC1025506	0.1110158	0.1890533	0.1251529	0.4048225	0.4663987	0.4719128
Ntmt1	1.371999	1.2478562	1.3002523	1.0242287	1.0345065	1.0048685
Alcam	0.7251053	0.9503064	0.8310996	1.1477937	1.09512	1.2272903
Zfp709	0.4778663	0.5011094	0.4790249	0.7374497	0.7177599	0.7874036
Frem2	0.1310457	0.2244167	0.1797882	0.3659306	0.4244317	0.4317484
RGD1565117	0.0124128	0.0206326	0.0239283	0.1885039	0.184517	0.2071262
Hba1	2.8192894	2.8470955	2.9583212	2.681304	2.3348253	2.4618343
Tsen34	0.9580519	0.2358221	1.1956888	0.2019766	0.2228845	0.183681
Nfia	0.7142144	0.7806985	0.7454168	1.0480972	1.0641252	1.0507954
Snrnp48	0.5881232	0.6561044	0.6743426	0.9121052	0.974229	1.0001592
Calcrl	0.5411716	0.6901047	0.6357998	0.8693578	0.8696543	0.9654286
Borcs6	1.2143553	1.1569968	1.1899671	0.8826868	0.9660785	0.8941877
Zfp62	0.4669395	0.5577561	0.5362849	0.7623498	0.7386396	0.8258116
Thy1	1.1352067	1.0341694	1.0037311	0.7557417	0.6966645	0.6944227
Ubxn2b	0.5093405	0.592508	0.5962443	0.8470448	0.8041481	0.8687243
Zbtb1	0.4921486	0.5327802	0.5162858	0.7894334	0.7559155	0.7225896
Csmd1	0.1906466	0.2395841	0.1555406	0.4741384	0.3677859	0.3737759
Pgr	0.0210632	0.048882	0.0402404	0.2662853	0.2161102	0.1801624
Rsbn1l	0.5731039	0.5945466	0.612539	0.865887	0.8549501	0.8723809
Gda	0.6043882	0.9027445	0.6767915	1.1985024	1.1064089	1.1759956
Stx19	0	0	0.0228837	0.1752066	0.1453869	0.2548517
Sass6	0.0341599	0.0236418	0.0280064	0.1831057	0.1655378	0.1362596
Slc7a2	0.0279428	0.0215484	0.0516973	0.1615294	0.1706785	0.2125487

LOC691170	0.4870545	0.545219	0.5418545	0.7487632	0.7439944	0.8194908
Inafm1	1.3632773	1.2218634	1.2776918	0.9010866	1.063116	0.9745406
Tnfsf15	0.2044599	0.2316411	0.1434953	0.7214792	0.6961476	0.5636513
Sult2b1	1.2964151	1.1624102	1.2181902	0.7480505	0.9362184	0.929312
Klhl42	0.4478066	0.5506111	0.5055468	0.7988971	0.8067447	0.8061258
Tspan1	1.2429076	1.2499636	1.0810391	0.9251689	0.9385603	0.8769843
LOC100909{	0.1700301	0.2451996	0.2207553	0.4637417	0.4754867	0.4773606
Fxr1	0.965996	1.0664304	0.9212398	1.2520893	1.307764	1.3392943
Irs1	0.2226404	0.2857498	0.2903747	0.495046	0.5530184	0.5484831
Abcg3l3	0.7024007	0.7475639	0.6346347	1.0142471	0.9277608	0.9543741
Thap2	0.4942125	0.6146281	0.5920755	0.8496084	0.8496935	0.7700972
Katnbl1	0.2113931	0.2661394	0.2565857	0.6035458	0.534125	0.5968538
Prex2	0.2057251	0.2297831	0.1600873	0.4556442	0.381631	0.4366423
Arl5b	0.5119187	0.5692233	0.4968465	0.8219442	0.7658676	0.7524165
Lilrb3	0.4795733	0.6040039	0.2903808	0.9467265	0.8600114	0.8280431
F2	1.0610505	1.4091634	1.4261868	0.7127384	1.0387224	0.8555957
Cyb561d2	1.3199197	1.2239492	1.2856786	1.0013684	1.0062632	0.9698263
RGD131042{	0.4546643	0.580856	0.5418232	0.834763	0.8679526	0.9218031
LOC102551(	0.0432057	0.1306761	0.1642905	0.4787146	0.5437597	0.5891775
Traf6	0.411865	0.4709732	0.520254	0.8020939	0.8008371	0.7860958
LOC103690(	0.3786467	0.213496	0.1762999	0.7544422	0.6984536	0.8076698
Lrrc31	0.2714457	0.3644662	0.3393829	0.5858347	0.5774075	0.6666955
Magi2	0.1500502	0.1753996	0.1700492	0.4083136	0.4341605	0.4033863
Zfp597	0.2056239	0.187205	0.2632775	0.5878034	0.5628363	0.6396762
LOC100911(	0.1856061	0.1415925	0.1657683	0.7343154	1.0241051	0.2025127
Abcb11	0.5695515	0.8279383	0.784058	0.9013883	1.1065553	1.2477027
Plcb4	0.3371802	0.4235749	0.3855759	0.6074281	0.6092962	0.6213611
Cyp2d5	1.9835738	1.9555855	2.0663516	1.0610927	1.3185377	1.7456527
RGD155990{	1.7339412	1.56817	1.6294472	1.398935	1.3439035	1.3587369
Fancm	0.3122378	0.337904	0.349717	0.560131	0.5451629	0.5867056
Jund	2.2481836	1.9595004	2.0149768	1.792757	1.7697973	1.795325
Aqp4	0.8717127	1.222404	1.0134684	1.3270474	1.4421661	1.4062242
Nrgn	0.9147284	0.9611863	0.9590985	0.5100803	0.5296828	0.6571772
Prkg1	0.0544306	0.065141	0.0405312	0.2558053	0.263277	0.3032537
Slc4a7	0.3503396	0.5293236	0.5681275	0.7893573	0.8556604	0.7042392
Zfp329	0.2788655	0.3425432	0.3426046	0.601634	0.5730134	0.5720943
Nnat	0.4305616	0.3907008	0.335956	0.8011864	0.8564638	0.8429917
LOC102553{	0.0211322	0.0315983	0.0158815	0.1030681	0.1219092	0.1469423
Stc1	0.5325799	0.9127012	0.8313627	1.213602	1.604821	1.0530218
LOC304239	0.005328	0.0059563	0.0077845	0.0924798	0.0564066	0.0864111
LOC103689{	0.387206	0.6232872	0.43944	1.0065787	0.9826565	0.7252713
LOC1036901	0.2499218	0.4856904	0.3405503	0.9153512	0.8357985	0.8536044
Ston2	0.0984968	0.1026476	0.075719	0.2338402	0.3438121	0.4310158
Slc10a2	0.3411328	0.5248932	0.4163612	0.7105259	0.7546792	0.717466
Srebf1	1.5014044	1.2417763	1.414882	1.1436128	1.0746364	1.1605463
Rex2	0.4856346	0.6022261	0.5142231	0.7786607	0.7464629	0.9028223
Phlda3	1.0759584	1.1224795	1.0925007	0.7888071	0.7924732	0.7897849
Zfp800	0.3591253	0.5009623	0.4657436	0.7003702	0.7030978	0.7617164
Zbtb10	0.2738194	0.2831742	0.2465081	0.5387336	0.5812397	0.5301107
Cep162	0.3132594	0.3917914	0.4303367	0.6158859	0.6238326	0.6816408
Pde1c	0.5143135	0.527525	0.5107971	0.7395712	0.7524552	0.8437437

Phykpl	0.8749608	0.8913607	0.8787414	0.2699734	0.2209941	0.5958892
Mpp7	0.1926489	0.2897518	0.1712044	0.5107868	0.5350985	0.5819093
Col10a1	0.0028726	0.0032125	0.0014054	0.0333205	0.0274499	0.0568583
Spint3	0.0185038	0	0	0.1518044	0.1258834	0.0999443
Mir186	0.3033568	0.4307411	0.4734291	1.102409	1.0749443	1.2602347
RGD1561034	0.3308132	0.2918504	0.2717071	0.6627555	0.7288136	0.7015462
Cpz	0.8704075	0.8339697	0.8366793	0.5232524	0.5385388	0.5569043
Syne3	0.3810723	0.4641528	0.5251896	0.7062091	0.7358564	0.7170543
LOC499229	0.0857955	0.1590566	0.0887927	0.4149628	0.2360976	0.355466
Samd5	0.1803872	0.2082003	0.3004678	0.5451119	0.6124719	0.5635009
Trpv5	0.3207335	0.3569068	0.3276724	0.6053954	0.5787623	0.6016013
Tet1	0.0107866	0.0135327	0.0092359	0.0833322	0.0607398	0.0768851
LOC100911[illegible]	0.3560135	0.3749399	0.3873899	0.6842187	0.5990859	0.6102846
Palm2	0.0693555	0.0625714	0.0741042	0.2649536	0.2499874	0.3419942
Fam228b	0.1083782	0.1627179	0.2139982	0.4580296	0.5055337	0.4918897
Cecr2	0.2870684	0.3511078	0.3374483	0.5524668	0.5155341	0.5379066
Wee2	0.0057465	0.0127539	0.0014103	0.0820896	0.0570212	0.0638462
Lrrc75b	0.8614231	0.8111109	0.8285796	1.1537398	1.1494726	1.290731
LOC102551[illegible]	0.416493	0.528462	0.4495714	0.7513113	0.6935694	0.7663347
Zfp385a	1.0146454	0.8866042	1.0155521	0.6863261	0.6991262	0.7208407
Kcnj2	0.4973012	0.4741491	0.5137545	0.8118247	0.7107647	0.7167246
Klhl28	0.2622185	0.3216675	0.2671439	0.5505351	0.5100737	0.5398818
Bloc1s3	1.2451143	1.0670319	1.2107925	0.8138667	0.9379331	0.9090373
Adra1a	0.6170109	0.5145201	0.5302509	0.899666	0.8416502	0.7920919
Snta1	1.8707606	1.5441392	1.6813625	1.4161315	1.428735	1.3954355
Faap20	1.2166931	1.114845	1.0896849	0.7995907	0.8725924	0.8894598
Klk1c2	0.0214601	0	0.0106137	0.010447	0.5368914	0.8327475
LOC100360[illegible]	0.2706268	0.4193437	0.3105969	0.574734	0.5755234	0.5956651
Seli	0.4938849	0.573186	0.5491718	0.8286221	0.8487393	0.833376
Herc6	0.2824467	0.4176987	0.3621628	0.7084344	0.5733533	0.7044004
Dnajb14	0.6276405	0.6783644	0.6349606	0.9752122	0.9829224	0.9595341
RGD1561931	0.1341653	0.1199271	0.1391814	0.2523784	0.2796166	0.3393401
LOC102555[illegible]	0.0557915	0.0464953	0.0704763	0.1993648	0.252947	0.2938041
Fam126a	0.2445917	0.3189027	0.2599593	0.6430968	0.5822247	0.6605157
Stk32a	0.427907	0.4018524	0.375439	0.7632168	0.7383403	0.8530665
Kpna5	0.4247712	0.5409703	0.4619458	0.7680471	0.7232091	0.7868613
Usp2	1.3846003	1.5751783	1.6514682	1.22343	1.3628452	1.1907461
Zfp524	1.2904561	1.2785116	1.2938144	0.9626844	1.0658722	0.9508311
Man2b2	1.5780151	1.6377666	1.3687206	1.2015434	1.3463521	1.1674766
Sycp3	0.353902	0.3698466	0.3627469	0.7719684	0.8245319	0.7078423
Tff3	2.7582472	2.3000529	2.2799027	1.8505033	1.702561	2.1827244
LOC100909[illegible]	1.0424938	1.2847804	1.1447567	1.7202624	1.3607473	1.4494351
Cdhr2	1.5672419	1.5128831	1.4858208	1.0779992	1.3700862	1.0234196
RGD131026[illegible]	1.0624473	1.0245555	1.0412192	0.7642306	0.8082224	0.746191
Bmt2	0.5935705	0.6137476	0.4740828	0.9830756	0.9279239	0.9401821
RGD156623[illegible]	1.5249669	1.4067428	1.4002043	1.1531561	1.2079193	1.1109447
Ralgps1	0.3304111	0.398914	0.4240408	0.6149092	0.7350154	0.7484683
LOC100911[illegible]	0.0970529	0.0569322	0.0731509	0.1040787	0.4782245	0.6810402
Sult4a1	1.0690048	1.2075714	1.1701309	0.4239594	0.8145724	0.8231099
Itga4	0.0598693	0.1480488	0.0691603	0.3402829	0.2639675	0.311517
Zscan30	0.4898166	0.6324863	0.570209	0.8069465	0.7973278	0.8532198

Ptgds	1.9348163	1.0385996	1.6721515	1.0570377	0.9714685	1.1152226
Cfap97	0.5363252	0.6034439	0.5117274	0.8339507	0.8513221	0.8510838
Agbl3	0.3065609	0.3504982	0.345843	0.5540427	0.5114283	0.5776707
LOC102550	0.0017122	0	0.0033388	0.0378796	0.0384101	0.0252151
LOC102553	0.1890893	0.2321627	0.196504	0.4971994	0.4035509	0.5147999
Fam149a	1.3840474	1.4378134	1.1130093	1.6573955	1.5644855	1.7212485
Nkap	0.729801	0.8923572	0.760438	1.1356283	1.139132	1.1398109
Tpm2	1.3911638	1.5547831	1.2988882	1.1645553	1.0384294	1.2172489
Mfap3	0.5721748	0.5646931	0.5253904	0.8277304	0.8774896	0.9082003
Micu3	0.4171006	0.4575594	0.466691	0.7393332	0.6781112	0.6847041
Akap17b	0.3245526	0.3811925	0.3407875	0.5952507	0.5243991	0.6560615
Elmod2	0.2669871	0.2199405	0.2223436	0.5058703	0.514905	0.52142
Card6	0.4134923	0.4886195	0.4405665	0.7175663	0.6139506	0.7051951
Tab3	0.304971	0.3550778	0.3512526	0.5420152	0.5157866	0.5424445
Zfp758	0.3631001	0.4950233	0.4359727	0.686366	0.6611203	0.7420695
Sorcs1	0.3965811	0.4256926	0.4237707	0.6303206	0.5862354	0.7304603
Slc26a2	0.1690284	0.1724763	0.1232214	0.3701859	0.3254427	0.4613374
Duox2	0.0332581	0.6383494	0.0284093	0.0142111	0.0138481	0.0032366
LOC100910	0.1166723	0.1810377	0.140229	0.394128	0.4773508	0.2588977
Tmem170b	0.2516819	0.1658493	0.313914	0.717493	0.6493065	0.6278763
Pik3ap1	0.84557	1.0642022	0.9929016	1.2152946	1.125978	1.4331326
Fam129a	0.387596	0.5401524	0.3655096	0.8202277	0.6411075	0.7797562
Lcn2	0.7013903	1.7076829	1.2083123	0.7583375	0.5895942	0.6060447
Slc4a8	0.125624	0.0856689	0.0915526	0.2535932	0.2920839	0.3986653
Dbp	2.0543654	1.8884634	2.1515917	1.4392897	1.6886867	1.8589878
Ammecr1	0.0691417	0.103905	0.0651434	0.2607488	0.2769158	0.3058287
Melk	0.7610136	0.6445201	0.7948687	0.4544365	0.460971	0.4037685
Ss18l1	0.8152522	0.8410979	0.9008235	1.1442586	1.1711622	1.1398017
B3galt2	0.2513784	0.1964428	0.1661663	0.5969776	0.3826169	0.4623887
Ubxn7	0.1093247	0.1703654	0.1017729	0.3744142	0.3986181	0.3693529
LOC102548	0.0075876	0.0063753	0.0236438	0.0849375	0.0931709	0.0910909
Nrtn	1.2608535	1.0735657	1.2064917	0.7081513	0.9184975	0.8264421
Wasf3	0.4952536	0.52989	0.4822493	0.7734773	0.7460566	0.7611502
Zfp507	0.4357757	0.4672663	0.475595	0.6715684	0.7249196	0.7591846
Pdk4	0.6891938	0.9426295	0.7259018	1.1498377	1.3962491	1.0324011
Gcnt6	0.5698198	0.5627284	0.6230843	0.8380748	0.7849633	0.9179191
Naaladl2	0.1046434	0.1400219	0.109094	0.3168916	0.2065328	0.3223427
Capg	1.3389908	1.7509266	1.4669822	1.138382	1.2050504	1.3102235
Alkbh2	1.2226342	1.1539535	1.1873188	0.8943788	0.9041869	0.8886803
Csgalnact	0.472864	0.4778405	0.4485133	0.7478938	0.7210023	0.7268704
Mrgbp	0.1227326	0.4673606	0.442136	0.0432223	0.047146	0.0969226
Mbd5	0.3475826	0.3640652	0.3741351	0.5733554	0.5579676	0.575041
Cacng5	1.7509079	1.1611982	1.450717	0.8874925	0.7789223	1.2263922
Pou2f1	0.326652	0.3300062	0.328278	0.5286218	0.5306266	0.648246
LOC100909	0.0128661	0.0103117	0.028219	0.1359265	0.0647877	0.1297613
Tgfb1i1	0.881174	0.8861351	0.921971	0.5877821	0.6561338	0.5848572
Ctdspl2	0.3267183	0.4116974	0.5307927	0.8128231	0.8298161	0.7716112
Glcci1	0.5868805	0.6366273	0.5852528	0.9110386	0.8512121	0.876977
Kbtbd3	0.3713637	0.4784621	0.4918454	0.7102209	0.6590305	0.7088756
Adgrg6	0.4279294	0.5919853	0.4299856	0.7611145	0.712184	0.7456406
Setd7	0.2228481	0.2838159	0.3322073	0.6719314	0.5641504	0.6052675

Mbtps2	0.5254717	0.6441906	0.600791	0.8855366	0.8238685	0.8361748
Hydin	0.0656985	0.0688798	0.0728061	0.1742344	0.145058	0.2184705
Reep6	1.3789557	1.4901709	1.4382902	1.0646555	1.3095831	1.0296125
Myo1b	0.1906796	0.3097729	0.2079514	0.5700434	0.4759613	0.6008048
Nsun3	0.1118209	0.0956566	0.1766669	0.4303567	0.4088949	0.4689624
LOC681177	0.1284078	0.2557117	0.2328522	0.5872743	0.5458445	0.4578204
LOC108349	0.0144074	0.0160868	0	0.1084687	0.0839274	0.1232409
Rsl1	0.2176634	0.2872077	0.3322945	0.5934848	0.528736	0.6538806
Zfhx3	0.1396264	0.1890949	0.1422518	0.2956437	0.2444203	0.3178204
LOC100910	0.0208439	0.0101187	0.04791	0.0968441	0.1585493	0.3423216
Grm3	0	0	0	0.0194486	0.008082	0.0093164
Opcml	0.112491	0.0812786	0.0947777	0.254256	0.2726699	0.2693494
Sptlc3	0.0066028	0	0	0.1266864	0.0122839	0.1427437
Map9	0.3298753	0.4549592	0.3835197	0.6635455	0.5901438	0.659126
Six4	0.1727013	0.204784	0.1374999	0.3140855	0.3902805	0.4404436
Sgip1	0.193922	0.194322	0.150595	0.4256706	0.3048293	0.3588194
LOC103690	0	0.0012022	0	0.0395241	0.0282797	0.0034756
LOC102555	0.3355678	0.4588226	0.3977742	0.7087962	0.6093677	0.7118425
Abra	0	0	0	0.0775619	0.0395052	0.1116492
LOC499407	0.0525597	0.0676657	0.0650462	0.1994846	0.2000543	0.190963
RT1-Bb	1.6627275	1.6104529	1.2694683	1.1996565	1.224122	1.2812331
LOC1009101	0.105236	0.1568357	0.0688886	0.3109999	0.3141702	0.3324599
Duoxa2	0.0375316	0.8985722	0.0539444	0.0093201	0.0179754	0
Hoxc6	0.2008621	0.2824357	0.2033377	0.3661913	0.4281031	0.3956186
LOC100360	1.418749	1.439159	1.4678645	1.0038503	1.2177904	1.0243147
Map3k1	0.2246326	0.2709285	0.247612	0.4192725	0.5629122	0.4638961
Eya4	0.0833581	0.12173	0.0507716	0.2665879	0.290257	0.3372704
Zfp770	0.3982865	0.5025445	0.4376216	0.6418288	0.6877828	0.705942
Acta2	1.5824143	1.6943032	1.4282507	1.3217246	1.1340768	1.4108032
Ren	1.1874032	1.028299	1.4020207	0.9026826	0.9386218	0.8165643
Spred2	0.8482779	0.9272193	0.8317147	1.1489992	1.212376	1.1456376
Phf6	0.0791683	0.0649359	0.0741731	0.1801489	0.2898623	0.2452652
LOC102550	0	0.0196665	0.0069703	0.0873933	0.0908329	0.0877059
LOC102556	0.1643091	0.1802559	0.1137624	0.4258888	0.4095499	0.452592
LOC686967	0.214438	0.2117128	0.3293187	0.6347541	0.5832703	0.4642443
LOC102551	0.1540359	0.2351907	0.1963961	0.337772	0.4170189	0.5004036
LOC1083481	1.7205961	0.6399807	0.5094744	0.3355604	0.4938347	0.3956664
RT1-Db1	1.7642673	1.7005021	1.3265208	1.2427472	1.2491549	1.3964334
Tbc1d24	0.291394	0.3755238	0.3638421	0.5450248	0.652442	0.6354188
Odf2l	0.3138321	0.3922165	0.32753	0.5606396	0.5763787	0.5902324
Mcc	0.1398341	0.142873	0.0710691	0.3382823	0.3921951	0.3486447
Apob	0.125987	0.1232493	0.0738969	0.4317191	0.1981664	0.2233632
Dnajc6	0.3705451	0.3372912	0.3697177	0.5930127	0.6028005	0.5421715
Inpp4b	0.2185249	0.2620507	0.2335587	0.4537092	0.3953366	0.4987328
Mrap	1.312759	1.1655028	1.1151244	0.7833647	0.8458255	0.9422386
Ccdc92	0.8305243	0.8400592	0.809601	0.4721821	0.5627698	0.5865305
LOC108348	0.1171373	0.1742753	0.1295146	0.3380115	0.3525787	0.4056927
RGD156398	0.3991723	0.4918583	0.4592029	0.74238	0.7915257	0.8134202
Slit1	0.1792951	0.1587796	0.2650176	0.0272056	0.0720702	0.0571086
Wisp1	0.2771267	0.3359714	0.2072744	0.6173635	0.5423801	0.6455972
Xkr4	0.0681527	0.0275724	0.0425818	0.207877	0.1563645	0.2263299

LOC1009125	0.380914	0.2569826	0.2905488	1.1252386	0.2471858	0.9536455
Zfp169	0.2028881	0.1800323	0.1913464	0.4720989	0.3748174	0.4904844
Nudt22	1.3385727	1.2095991	1.2579213	0.9544371	1.052293	0.9782283
Gucy1a2	0.0406598	0.0763299	0.0673576	0.2903266	0.241255	0.1822943
Tmem253	0.179805	0.2414935	0.1921915	0.6054316	0.5537616	0.4545229
Gk5	0.0309023	0.0453251	0.0515636	0.1698277	0.1523092	0.1813099
Cenpf	0.0037342	0.0429112	0.0196949	0.1352107	0.1328319	0.1135035
Dxo	1.2324712	1.0964831	1.1408065	0.8562873	0.935368	0.8763202
Akr1c1	1.7586276	1.7976808	1.5835343	2.0422661	2.2025132	1.8393836
Hgf	0.3039756	0.400893	0.3406025	0.6464056	0.5417919	0.6749485
Ppp1r15a	1.397686	1.121705	1.1055647	0.9208942	0.9604145	0.9649182
LOC1025546	0	0	0	0	0.1219113	0.0800429
Zfp551	0.1013412	0.0879189	0.1061879	0.2433553	0.2787893	0.3771255
Mdfi	0.7611923	0.7719766	0.6637477	0.3888213	0.4515399	0.3966427
LOC1036905	0.0225455	0.0085449	0.0074701	0.0960639	0.0790241	0.1226632
Polr2l	0.4894206	0.361085	1.3356773	0.2767575	0.1223989	0.0512579
Muc6	0.0097826	0.0059957	0.0086979	0.0404241	0.0470447	0.0407979
LOC1003610	0.4092178	1.96395	0.1920558	0.2492903	0.1728052	0.3679929
Klf2	1.5846982	1.3078264	1.3638272	1.1791034	1.1695831	1.0747021
LOC1009113	0.018041	0.0135259	0	0.0950438	0.2665274	0.0731265
LOC1083504	0.0251697	0.0057597	0.0340657	0.1247235	0.1570868	0.1579277
Nfxl1	0.4683713	0.5490006	0.4868519	0.7368489	0.7261956	0.7517984
Slc26a7	0.3555964	0.423168	0.5389204	0.6270105	0.75199	0.7168538
Rnf125	0.5910657	0.6212084	0.6238157	1.0204403	0.9090199	0.9147543
Epm2a	0.1517587	0.0354612	0.2681752	0.4890399	0.6345908	0.6425395
Pak2	0.1241127	0.0941123	0.323477	0.4050101	0.5559447	0.8808731
Map1b	0.2509061	0.292454	0.2692019	0.5195595	0.3750804	0.4224194
Nt5dc3	0.4524727	0.3786926	0.4911888	0.6220782	0.7110686	0.8092948
Plag1	0.0543376	0.1039649	0.0657642	0.1710489	0.2250811	0.2820831
LOC1083481	0.0475887	0.0478978	0.0588703	0.1853975	0.1305265	0.1397819
Igf1	0.8158319	0.7119472	0.6187205	0.9369731	0.8717866	1.1519998
Mkl2	0.0786354	0.1460379	0.1795137	0.3837442	0.3647111	0.395269
Igfbp4	1.6864677	1.6001178	1.7255025	1.5657455	1.2444349	1.2756241
LOC1003635	0.1731237	0.3092471	0.3315697	0.6203277	0.572818	0.646127
Zscan4f	0.0054275	0.0407933	0.0503184	0.1886469	0.1462379	0.2654702
Adra1b	0.9287769	0.8444861	1.0257144	0.6440708	0.6942165	0.7357406
Med12l	0.0275216	0.0818408	0.0781084	0.2802701	0.2354692	0.1914296
Dsg2	0.3470942	0.3088933	0.3008816	0.509249	0.4888459	0.621576
Usp26	0.1311034	0.1129044	0.1352821	0.3063418	0.2806701	0.353253
Rpl7a	1.2559205	1.8409138	1.2873555	1.0892108	1.1363149	1.0565351
Kdm4c	0.4100212	0.5388972	0.419952	0.6883408	0.6460238	0.7923045
Rel	0.1748892	0.2085396	0.146061	0.4437313	0.3735322	0.4529483
Ghrl	0.0361992	0.0945103	0.0520634	0.4898115	0.3697592	0.2434445
Tp53i13	1.1106249	1.0465949	1.0381834	0.7322166	0.8463548	0.7909095
Ptchd1	0	0	0.0068359	0.0571001	0.064337	0.0294732
Slc40a1	0.5203102	0.539769	0.4887224	0.7686731	0.7560988	0.7707605
Zfp398	0.1910268	0.2686484	0.2410594	0.4416587	0.5056849	0.4712133
Dnal1	0.3771339	0.4024462	0.3243631	0.7543918	0.7148798	0.6838984
Lepr	0.2730461	0.3422073	0.3374102	0.6267084	0.4498378	0.662504
Nhp2	0.295634	0.189084	0.2187902	1.1728158	0.1090307	1.1288075
LOC1025546	0.3188618	0.3282709	0.3325477	0.5594349	0.4951217	0.6073577

Ctsz	1.1871189	1.4485859	1.1278458	0.9886139	0.9118439	1.045566
Zbtb37	0.194563	0.1956377	0.1831172	0.4273477	0.4187741	0.488131
Zfp655	0.2918188	0.250162	0.3273621	0.5211305	0.5323452	0.5211397
Myo3b	0.3309289	0.3527154	0.3541862	0.5450666	0.5629897	0.6352613
Gipc3	0.6502947	0.6740614	0.7949515	0.3361886	0.5154279	0.3592049
Ptp4a3	0.9000527	0.9665332	0.8646283	0.6422165	0.6719638	0.6408105
Tmed6	1.5597982	1.3067232	1.5245884	1.1179109	1.2311932	1.2503468
LOC103690(	0.00575	0	0.0083992	0.2012168	0.0945918	0.0092846
Slf1	0.4372013	0.4987651	0.4795083	0.6875049	0.679489	0.7497594
RT1-M6-2	0.3444719	0.1680164	0.4094729	0.5905423	0.6600435	0.9004923
LOC102548(	0.0018235	0.0030557	0.0017813	0.0201642	0.0118147	0.0126522
Fsd1l	0.1412976	0.2416772	0.2090563	0.3578346	0.4066955	0.4044539
Ston1	0.4466197	0.5103735	0.4281224	0.7249472	0.7211707	0.6788023
Adam11	0.1389737	0.0873883	0.1556197	0.3928047	0.235416	0.5019611
Grik2	0.193513	0.2323743	0.4446623	0.6229349	0.7246877	0.5622891
Ptgfr	0.2719177	0.3487517	0.4532818	0.5619526	0.7261459	0.6593897
LOC688981	0	0	0	0.0403898	0.0135301	0.0487222
Anln	0.0324553	0.056093	0.0170862	0.156663	0.1413366	0.1444762
RGD156045!	0.0127267	0.0279762	0.0154898	0.1461932	0.0670959	0.1210451
Gja4	0.9288912	0.986089	0.8463983	0.6749726	0.6218492	0.6313987
Scgn	0.0533674	0.0125231	0.0422508	0.1695624	0.2074018	0.3617689
Pnpla5	0.0291259	0.0869705	0.0164975	0	0	0
Ppp1r14a	1.1162409	1.0861437	1.1594109	0.7628807	0.8132734	0.7517185
Trpc1	0.2749845	0.3359729	0.3425845	0.5529996	0.5462893	0.5848274
LOC690044	0.0472666	2.3913491	0	0.0867346	0.1035115	0
Klhl15	0.0680653	0.1441705	0.0711516	0.2787762	0.2868113	0.3021794
Myh1	0.0123358	0.0205057	0.0075726	0	0	0
Pex11g	1.0609594	1.0294689	1.012925	0.7092864	0.805328	0.7548776
RT1-A1	1.2633371	1.0837082	1.4605333	0.9789761	1.0456517	1.0000212
LOC102553(	0.1083024	0.1099319	0.100332	0.3367659	0.2860494	0.2850336
Wdr44	0.2016446	0.3541762	0.2496372	0.4886905	0.5027154	0.5797314
Wnk3	0.0037009	0.0163215	0.0116417	0.0669182	0.0546975	0.0429948
Cd163l1	0.0660575	0.3991622	0.1534006	0.031732	0.0527745	0.0151558
Zyg11a	0.0276156	0.0207611	0.0114524	0.0995773	0.0882776	0.1065097
Rasef	0.0820031	0.1283	0.1718553	0.3387114	0.2837964	0.5242646
LOC102551{	0.044655	0.0337325	0.0705195	0.1719613	0.2286297	0.3083448
Adam4l1	0.0260165	0.0746207	0.0877765	0.3465128	0.2413495	0.1803463
LOC108351!	0.0809208	0.0730978	0.0414057	0.2815414	0.1700972	0.2400354
Nek1	0.1930441	0.294261	0.2652401	0.5067666	0.4434243	0.4611641
Baat	1.0178921	1.2913116	1.2754734	1.375881	1.4084904	1.7026609
Dnhd1	0.1539337	0.1777535	0.1878744	0.3082683	0.2819858	0.3252514
LOC102554:	0.5025206	0.6407817	0.5730291	0.8156693	0.8167293	0.8963329
Mcpt2	0	0.0274934	0	0.3601308	0	0.6656417
Trpc5	0.0442029	0.0708458	0.0503245	0.2004699	0.1285852	0.2317022
Ier5l	1.2557959	1.0286416	1.0361024	0.6341382	0.7392672	0.9483518
Sytl5	0.0633074	0.0570767	0.0502378	0.1967967	0.1370443	0.2035565
Fam131c	1.1615058	1.0476917	1.2392968	0.6709214	0.9731358	0.8219459
C1qtnf5	1.0314754	0.9056582	0.8945981	0.7193568	0.5755016	0.6493659
Ttll10	0.546242	0.6229485	0.7412505	0.8683748	0.975045	0.8824289
Adora2a	0.6320877	0.6420185	0.6398798	0.4115439	0.353433	0.453729
Zfp780b	0.2534098	0.3758203	0.2995925	0.4965251	0.5189168	0.5597621

Ptp4a1	0.4088324	0.4058504	0.3986504	0.6156722	0.668374	0.6879705
Mdk	1.4669642	1.4102949	1.2564399	0.9457032	1.0703094	1.2266746
Glycam1	0.1603266	0.5060077	0.5878747	0.0580763	0.1298234	0.0795799
Mphosph9	0.3376749	0.3418673	0.3730905	0.6113059	0.5823415	0.534301
Xylt1	0.2152841	0.2100877	0.1913551	0.4880735	0.3414233	0.3715204
Rhobtb3	0.153802	0.2123342	0.1704507	0.3905306	0.3550504	0.3762103
Ccdc150	0.0281709	0.0413784	0.0203634	0.1240781	0.1044572	0.1201526
Zbtb26	0.3164428	0.4588259	0.3697353	0.6097074	0.6360884	0.6223859
Ppargc1b	0.482021	0.4355277	0.3991616	0.6465714	0.6913215	0.7261027
Rab27b	0.0728428	0.0958169	0.0487985	0.2384041	0.1567281	0.3916287
Fndc10	1.2991114	1.5066645	1.6133554	0.9505094	1.3545198	0.9692422
Cd46	0.1059827	0.1327456	0.0582079	0.3126935	0.3085991	0.2863607
Mvd	1.0365852	1.0014395	0.8954279	0.7133162	0.7671433	0.6539715
Bicd1	0.0437102	0.0378731	0.0787843	0.2059615	0.1579164	0.1740646
Mmp7	0	1.1885006	0.0089107	0	0.0169252	0
Bend4	0.1338611	0.1548802	0.1270189	0.336917	0.2812645	0.330835
Cep135	0.1646586	0.2137693	0.2292154	0.3688697	0.364735	0.4933501
Etnk2	0.915668	0.9867287	1.0377962	0.4669491	0.7294567	0.6116335
Ror1	0.0933809	0.1328427	0.0850391	0.2534109	0.2690755	0.3188321
Tceanc	0.1716715	0.1202596	0.1430139	0.3698262	0.4285019	0.3834479
Grem2	0.4569719	0.8439415	0.6530995	1.0899531	1.0684511	0.8213456
Limd2	1.1348141	1.2110039	1.013855	0.9071543	0.7939232	0.8537868
Cyp2c11	0.9244205	0.4808596	0.6475996	1.3706505	0.5188865	1.8147571
Pde10a	0.1183249	0.0687826	0.0699678	0.2780722	0.2304975	0.2671104
Qrich2	0.2827955	0.2203402	0.2684053	0.1068248	0.1225284	0.1350923
LOC102554(	0.0226773	0.0445175	0.0321333	0.1424456	0.1041562	0.110488
Emp3	1.0897833	1.1790107	1.0351888	0.7392983	0.7728204	0.8436443
Rbfox2	0.3598611	0.3769237	0.3997547	0.6132968	0.6376157	0.6692409
Cox4i2	0.619929	0.6403246	0.7133792	0.3440128	0.342666	0.2989595
Cyp4f4	0.0508119	0.0779894	0.0377967	0.2613203	0.2016054	0.1737427
Trim69	0.0342702	0.0501765	0.0370734	0.1605457	0.1192756	0.2124803
Mgmt	1.4236462	1.3050993	1.2388109	1.0127829	1.0481972	1.1122486
LOC1083481	0.2156188	0.2752418	0.2454883	0.4430684	0.4517961	0.5302723
Cep126	0.2850821	0.3225742	0.3608629	0.5301716	0.5013373	0.555398
Phka2	0.1988488	0.154815	0.2144885	0.3621987	0.3891544	0.4086121
LOC1025528	0.258154	0.3421668	0.2748723	0.5122986	0.6031213	0.5357905
Tmc4b	0.3695871	0.0782919	0.3324165	0.046558	0.0786032	0.0421
Rassf9	0.6271963	0.678761	0.5539481	0.895304	0.8648969	0.9307919
Zfp483	0.0196502	0	0.0223241	0.0924739	0.110227	0.0911284
Cep295	0.2148695	0.2468361	0.2511509	0.4055041	0.386521	0.4305212
RGD1566325	0.2011472	0.3208779	0.2563746	0.4218006	0.4744756	0.4284573
Pgm2l1	0.1577378	0.2358792	0.1795463	0.4129247	0.3316695	0.3797937
RGD1562521	0	0.0083589	0	0.0447509	0.0436459	0.0389696
Adamts5	0.4071068	0.4655531	0.4399114	0.6521587	0.6786356	0.7111815
Olfml2b	0.8697604	0.6952592	0.6279151	0.4440831	0.3540973	0.5654344
Dhdh	0.352388	0.3826663	0.4484949	0.6768929	0.7154728	0.6725767
LOC1036899	0	0.0203803	0.0178471	0.2614231	0.017122	0.3572791
Pde6c	0.0208842	0.0232985	0.0051934	0.092291	0.0649974	0.1247635
Ncapg	0.0411256	0.0892274	0.0381884	0.2464549	0.1801107	0.1619762
RGD1562161	0.1023933	0.1503051	0.1318538	0.2810648	0.2974696	0.3792843
LOC1025554	0.6949836	0.3745298	0.5550343	0.9452453	1.014146	0.7526239

Cd36	0.6491945	0.9170808	0.7264782	1.0380872	0.9135265	1.1898728
Fam50a	1.0672523	0.1972433	0.2614446	0.1835044	0.1174074	0.1607433
Tbc1d32	0.2778115	0.3514643	0.2747844	0.5237722	0.4651444	0.5122116
Sytl2	0.2888476	0.1219871	0.1071518	0.4601521	0.4448297	0.3836721
LOC1009101	0.2840796	0.0663445	0.0584681	0.4878129	0.4109141	0.5582787
Nlrp1a	0.2281754	0.2951849	0.2209578	0.4584292	0.3780169	0.4545255
Filip1	0.1823341	0.2712349	0.2007072	0.4100549	0.3685829	0.4467678
LOC103690(	0.4412194	0.0903243	0.0311534	0.8266289	0.7833015	0.7524648
Afap1	0.2325231	0.2473501	0.2482121	0.5237424	0.4025023	0.5127355
Tbkbp1	0.57279	0.6238273	0.5922739	0.3947807	0.4103051	0.353616
Lancl3	0.0218578	0	0	0.0845817	0.0825825	0.1254019
Kcnq3	0.0032433	0.0036268	0	0.0153719	0.0308818	0.0272757
Wfdc10	0.6734541	0.5622095	0.9445504	0.1180829	0.362543	0.3433198
Cabp1	1.0655915	0.8410919	0.8722029	0.5084759	0.5571807	0.7185018
Sdf2l1	1.1589195	1.1682671	0.9665817	0.8179473	0.8082836	0.8398756
Zfp64	0.7947122	0.8189361	0.8669374	0.5035653	0.6380841	0.6177193
Nedd1	0.3391276	0.4545043	0.4810849	0.6362354	0.6751297	0.694316
LOC691254	0.0241517	0.0269322	0.056772	0.1326826	0.1493786	0.1804422
LOC689268	0	0	0	0.5343938	0	0
Skint10	0.2047827	0.184564	0.1893324	0.4984487	0.3892562	0.4748752
Ccnd2	0.4921285	0.5029102	0.3902577	0.8281484	0.6665799	0.8540793
Ten1	1.1017409	1.162112	1.0810835	0.7671719	0.8557848	0.8738809
LOC1025537	0.2720167	0.2900214	0.2559987	0.4877599	0.4545985	0.5048849
Hmcn1	0.0537463	0.1175016	0.0730476	0.1595079	0.1875429	0.2066496
Rgn	1.872186	1.6678073	1.6464338	2.1378079	1.7504628	2.2663203
Sycp2	0.0094167	0.0288403	0.0270369	0.0905325	0.0956181	0.089003
Gpr4	0.7172831	0.735917	0.6256057	0.4954591	0.4310106	0.3585134
Negr1	0.0302418	0.028259	0.0295638	0.1292446	0.1152312	0.1704633
Rbl1	0.1781783	0.2338504	0.196817	0.4256573	0.3810139	0.4755802
LOC102550:	0.4950949	0.4762246	0.5820869	0.7597628	0.8028468	0.8253724
Slc8a2	0.0448106	0.0435131	0.0210015	0.1430361	0.1607063	0.1285983
Mir196c	0	0.115241	0.1848829	0.6916816	0.7464916	0.5248984
Fscn1	0.7356977	0.7691229	0.6355464	0.4737077	0.4762039	0.4735601
Fign	0.10909	0.0720893	0.0666021	0.2075383	0.2000618	0.2754706
Unc5c	0.0062931	0.0162396	0.0112061	0.0332258	0.059963	0.0596574
Prox1	0.2428479	0.2417112	0.3515999	0.3700727	0.5044599	0.6120803
Setdb2	0.4256354	0.5154733	0.4773778	0.6931267	0.6702902	0.7514175
Mest	0.6275431	0.5204162	0.4188071	0.9089852	1.0604718	0.6847094
Diras1	0.1054866	0.0901455	0.1033091	0.0187203	0.0270872	0.0053444
Pafah1b3	1.2460606	1.2396864	1.2006727	0.8590502	1.0518099	0.9315721
Hdx	0	0.0162414	0.0142143	0.0934601	0.0701889	0.0502717
Mmrn1	0.2258192	0.3120991	0.2511962	0.4503301	0.4114724	0.5538797
Dzank1	0.2452781	0.219676	0.2592169	0.4161223	0.491393	0.5483998
Mmp9	0.3368779	0.3919936	0.1366519	0.0802465	0.0314012	0.126574
Lpar4	0	0.002524	0	0.0253466	0.0206857	0.0144317
LOC679739	0.1265293	0.5390134	0.2807032	1.6832722	0.1803046	0.820913
Garem1	0.4817858	0.5376443	0.5331175	0.7449677	0.7918142	0.7807283
LOC102550(	0.0065501	0.0145213	0.0063993	0.0963319	0.0631526	0.0840518
Zfp949	0.3019921	0.417609	0.4023339	0.5961528	0.5952928	0.6632978
Asb7	0.2892227	0.3533056	0.3245413	0.525266	0.5467434	0.5144001
LOC103691(	0.0241793	0.0269628	0.0349848	0.0907439	0.1161958	0.0863722

Pcdh9	0.051389	0.0311647	0.0153967	0.0722952	0.3091573	0.1015956
Goltla	1.2003628	1.1094636	0.8819804	0.7190279	0.7808249	0.6546667
Rem1	0.9778551	0.8434783	0.8484437	0.5574656	0.5565291	0.728043
Ccbe1	0.2744884	0.3616192	0.3160125	0.5597881	0.5744493	0.5541444
Paqr4	0.6403071	0.6853956	0.5615124	0.3733017	0.390453	0.3283528
LOC1025507	0.3254905	0.2958506	0.3847799	0.5927827	0.5704541	0.7488122
Map3k6	0.4271216	0.4957989	0.3993494	0.2705952	0.2622559	0.2178759
Map2	0.1707833	0.2832781	0.2405106	0.427944	0.3708785	0.3961362
Wdr62	0.0924742	0.1109261	0.0728969	0.0258848	0.02872	0.0229468
Cadm1	0.3917	0.4733266	0.4351248	0.6351995	0.6105926	0.7173726
Izumo1r	0.0496304	0.1618056	0.0167905	0	0	0
Csnk1g1	0.1755309	0.229006	0.2967778	0.4455888	0.4835567	0.5055707
Fam206a	0.3331807	0.4334921	0.4675656	0.5954251	0.648922	0.6492937
Cldn1	0.6419421	0.9346693	0.84997	1.1892094	1.2039524	0.8851158
Ufsp1	1.091897	0.9818109	1.0862817	0.7815002	0.8272405	0.787739
Caps2	0.0353623	0.0840519	0.0535399	0.1729764	0.186435	0.2071729
RGD1561166	0.5404385	1.9608022	0.1922821	0.4696569	0.3028658	0.5131972
Plk3	0.9003427	0.6581578	0.7102584	0.5559385	0.4571626	0.4736675
Samd12	0.0440123	0.0127654	0.0630601	0.1942061	0.2365419	0.2055374
Pag1	0.1744301	0.2366807	0.2128925	0.5155254	0.511908	0.412349
LOC500375	0.5113406	1.8429549	0.2180695	0.3358616	0.3060075	0.5265968
LOC1009125	0.1515268	0.1562184	0.1576592	0.8906405	0.0827018	0.5271763
LOC500175	0.8941342	2.1316014	0.3166569	0.8368207	0.3955093	0.5109876
Twf2	0.8495111	0.9262302	0.8572742	0.6388831	0.5337751	0.6799824
Zfp366	0.1774156	0.1459991	0.1491602	0.3832729	0.2959541	0.3627216
Cox11	0.4541581	0.5033532	0.4425885	0.6774261	0.7378484	0.7654882
Cdon	0.2770244	0.2336684	0.2554902	0.43438	0.4310149	0.4153148
Rps6ka5	0.288087	0.3002624	0.3241613	0.4440046	0.4791407	0.5484036
RGD1560289	0.1605967	0.1867558	0.1979383	0.3248463	0.3136311	0.3111386
Mef2b	0.3529494	0.3807297	0.4393086	0.0489145	0.195267	0.1467954
Strip2	0.2908323	0.2507431	0.2074278	0.4747911	0.4052804	0.473389
Giot1	0.2313191	0.3016401	0.2688936	0.4528647	0.4736144	0.5308777
Mis18bp1	0.0390708	0.0773049	0.0733161	0.2106873	0.1409523	0.2191337
Mrpl53	0.3818037	0.3466407	0.4911679	0.3591811	0.365323	1.7917661
Klf7	0.2086097	0.2561285	0.211868	0.4704281	0.4105212	0.4440105
Kbtbd8	0.252409	0.2444724	0.3022382	0.5142556	0.4267992	0.4836974
Pkhd1l1	0.0100641	0.0175404	0.0070465	0.0381252	0.0302459	0.0872962
LOC1036899	0.0055982	0.0124273	0.0108703	0.422626	0.0129929	0.0090405
H1fx	0.8657957	0.6690707	0.6636667	0.2889995	0.4751984	0.5866313
Npm3	1.0669943	1.0569247	1.156139	0.8215215	0.8548298	0.8146973
Olr338	0	0	0	0.0544137	0.0851808	0.0414157
LOC1083481	0.0034429	0.0127024	0.0111114	0.0361138	0.065935	0.0346931
Cebpd	1.4158202	1.2576024	1.2210499	1.0561644	1.1194397	0.7936103
LOC1025521	0.0899814	0.0600503	0.0880924	0.2449672	0.2079076	0.2170848
Kcns1	0.012118	0.0755219	0.0059607	0	0	0
Ido1	0	0.5976353	0	0	0	0
Tmprss9	0.5284696	0.2589042	0.4793888	0.1796573	0.2621499	0.1178821
Map3k7	0.2026787	0.2548796	0.2938127	0.1280769	0.107784	0.1071447
Nrxn1	0.0793264	0.0992976	0.1279363	0.3518263	0.1658955	0.2762333
Spin4	0.1582552	0.154557	0.1793356	0.2834617	0.3697301	0.3898571
Tex12	0.0431679	0.0533353	0.0302182	0.133689	0.1213416	0.1697955

Sac3d1	0.9699864	0.8889447	1.0040213	0.7348179	0.695309	0.6996865
Peli3	0.7410886	0.7132307	0.7206401	0.5445776	0.5245787	0.4158798
Ky	0.0135344	0.0076226	0.0385255	0.1799796	0.0500944	0.0917476
Rtkn2	0.0022493	0.009976	0.0108767	0.0432923	0.0364356	0.061279
LOC1025481	0.2834591	0.180415	0.2067799	0.5735761	0.3875204	0.418676
Rab8b	0.4323523	0.7148171	0.6033115	0.9692077	0.9799195	0.9362849
Aspm	0.0697639	0.0986209	0.0633798	0.1823755	0.1687477	0.1965372
RGD156088	0.0938307	0.1460931	0.1341057	0.3023259	0.2804159	0.259896
Gpsm3	0.7800101	0.9474127	0.776579	0.5545711	0.5564738	0.5633313
Zfp113	0.3156272	0.4053057	0.4172206	0.6029442	0.6143218	0.6183746
Kif20b	0.1487226	0.1135183	0.1445995	0.2852403	0.3042313	0.2883084
Musk	0.0700622	0.0489133	0.0630087	0.2310655	0.1736986	0.1645682
Igha	2.2346618	2.8043282	1.5682637	1.828811	1.8259933	1.8960899
LOC499643	0.0921412	0.0425074	0.0424211	0.2385681	0.1877909	0.2219489
LOC102553	0.0179239	0.005088	0	0.049803	0.0561737	0.1087823
RGD1307947	0.3730013	0.4105144	0.3883965	0.6101394	0.6468223	0.6313302
Gria2	0	0	0	0.0053471	0.0065017	0.0148664
Srrm5	0	0	0	0.0204162	0.0247337	0.055112
RGD156237	1.0000132	0.8113133	1.142354	0.6578201	0.5870968	0.7882736
Ankrd61	0.0934062	0.1032723	0.1401574	0.2839019	0.3298925	0.3125304
LOC1025567	0	0.00299	0.0077889	0.0346714	0.0267799	0.0212146
Epha4	0.3630323	0.2498609	0.3459139	0.497325	0.5680963	0.5307701
Pi15	0.0136716	0	0	0.0791184	0.0605934	0.0562455
Arr3	0.0355981	0.082871	0.041443	0.2278222	0.1360598	0.2881965
Efcab1	0.0153258	0.0295143	0.0365151	0.0627236	0.1625743	0.1603123
LOC100910	1.1001479	0.2185508	0.3684943	0.2081785	0.238882	0.2908926
Pak3	0.0062799	0.0023525	0.0101778	0.0760384	0.0249356	0.0200418
Nckap5	0.0610601	0.0431842	0.0557321	0.1366241	0.1237904	0.1962024
RGD156073	0.2265129	0.2203775	0.2078536	0.4989676	0.3682557	0.4647871
LOC100911	0.2056124	0.070268	0.0451392	0.009263	0.0265313	0.0104018
Gcnt7	0	0.0181213	0.0091358	0.069149	0.0461356	0.0662021
LOC100911	0	0.009105	0.0039984	0.0590463	0.0076334	0.1705646
Fam83b	0.0787178	0.0897237	0.0701584	0.1538714	0.2648901	0.2374247
LOC100910	0.234265	0.2117857	0.2426178	0.2916652	1.1108314	0.350647
Rnf24	0.391405	0.4079704	0.4926336	0.8383403	0.8410253	0.781328
Rps4y2	1.2049394	0.9841525	1.17871	0.7852853	0.9132377	0.8457486
Ift27	1.0699801	1.0270857	1.0186522	0.7516173	0.8208548	0.7809092
Chst12	0.5929848	0.7825986	0.5914417	0.3965672	0.3730256	0.4503343
Tpbgl	0.275382	0.294925	0.3550935	0.1318092	0.1213516	0.1565963
Klk1c6	0.0219086	0.0475738	0.0317359	0.1050172	0.0856501	0.5025765
LOC502908	0.0046449	0.0204099	0.0134733	0.1068972	0.0171469	0.2327323
Brd2	0.8147275	0.3568162	0.2956569	0.2637246	0.232376	0.2349254
Nek10	0.0107106	0.0070195	0.0087383	0.0358324	0.0230675	0.056691
Ccnt1	0.0114456	0.0252039	0.0300874	0.083425	0.1160446	0.0825231
Creb5	0.0311037	0.0358001	0.0521327	0.1607429	0.0788074	0.1209045
Ggt7	0.2731106	0.3178593	0.2475317	0.0992492	0.120428	0.1414276
Cybb	0.613636	0.9692585	0.7411974	1.1696752	0.9602191	1.1340198
Nol8	0.0672296	0.0684749	0.0919113	0.1738556	0.14933	0.3181659
Zfp458	0.092091	0.1948625	0.1820682	0.3228112	0.3931089	0.361434
LOC287274	0.1646385	0.4683453	1.0936608	0.3038063	0.1620159	0.1544445
Cask	0.3080809	0.444319	0.312034	0.5970716	0.596968	0.5525176

Hils1	0	0	0	0.0634314	0.0422289	0.0814553
RGD15648б1	2.3686025	3.267904	1.6250618	2.1333403	2.0421262	2.0451719
Fzd3	0.3422157	0.3855263	0.3943361	0.6200785	0.5399289	0.6368494
LOC1009106	0.3287807	0.026344	0.0174309	0.0057954	0.0112194	0.0065111
Atad5	0.1149076	0.1663312	0.1516284	0.2928927	0.2656082	0.275863
LOC1025474	0.2020387	0.209596	0.1851215	0.3453679	0.3694841	0.3472692
Ckap2	0.0181288	0.075821	0.0413654	0.1615882	0.1994049	0.1449599
RGD1560207	0.0049965	0.027238	0.0024475	0.0772435	0.0734208	0.0607309
Gpr153	0.2915752	0.2675411	0.2651156	0.1373957	0.1382054	0.1281438
Jade3	0.2485883	0.3574699	0.301172	0.5185709	0.4857073	0.6093479
F10	0.0966129	0.1880759	0.1351486	0.0439293	0.0325224	0.0252419
LOC1036901	0.0487784	0.040093	0.5429603	0.0089301	0.0338034	0.0479758
Caln1	0.6449741	0.4538459	0.6109364	0.7824143	0.9162905	0.8302185
Napb	0.4003127	0.4075325	0.3974309	0.6381589	0.656524	0.6147786
Zfp280c	0.2909778	0.4974897	0.4026746	0.6411015	0.6254203	0.6886871
LOC1036940	0.110971	0.1624611	0.2148059	0.4165064	0.3202268	0.4105084
Ier2	1.795103	1.3997425	1.2245489	1.0635173	1.0969549	1.3581223
LOC1036948	0.0322952	0.0301823	0.0416067	0.0260591	0.0278625	0.6622786
LOC1083513	0	0	0	0.0636544	0.062115	0.0599093
Mapk15	0.0945928	0.1257301	0.1169448	0.0198575	0.0317927	0.0434458
Nt5c	0.8897673	0.9246454	0.9190412	0.6172808	0.5860611	0.659288
LOC1036927	1.6987671	2.7761458	1.0005847	1.692273	1.2471498	1.0163513
Mmp23	0.8660761	0.7936431	0.7429811	0.5654601	0.5801027	0.5793918
Cenpe	0.0254799	0.0527145	0.0515753	0.1407701	0.1142408	0.1119026
Atad2	0.1359568	0.3154395	0.241764	0.4378269	0.4069676	0.4652338
Fkbp10	0.5790818	0.7095347	0.6572452	0.4217349	0.417325	0.4746
LOC1036948	0.608768	0.7499562	0.6989989	0.4593479	0.2783392	0.2735423
LOC1009124	0.1439464	0.1436756	0.070545	0.0462256	0.0127676	0.0147057
Rps6ka6	0.2093458	0.3031243	0.2428028	0.4260362	0.4234805	0.5119745
Sh2b2	0.3918476	0.2599689	0.3129558	0.1496428	0.1378394	0.1627798
Zfp300	0.0533233	0.0613656	0.059654	0.1508718	0.1266935	0.2107345
Ddx60	0.0363602	0.040482	0.0815966	0.188202	0.1039611	0.1826366
Ogfod1	0.4559635	0.4203884	0.4857918	0.7026653	0.7075102	0.7302702
LOC1009093	0.7052958	0.0845596	0.0882141	0.0736083	0.0583976	0.0589483
Hectd2	0.1197578	0.1636037	0.1386022	0.2896008	0.2674944	0.329116
LOC1003631	0.0143254	0.0463203	0	0.1106343	0.1676141	0.1782493
Zfp575	0.1866856	0.1418362	0.1498884	0.0455671	0.044443	0.0345987
Rab30	0.1635586	0.1980364	0.1427031	0.2967769	0.3058719	0.3051784
Mir336	0	0	0	0.4710561	0.2137452	0.3470118
LOC1009122	0.2634054	0.1876838	0.2131568	0.4599096	0.3957586	0.4271919
Cep97	0.2858013	0.3140251	0.3155557	0.5415558	0.4828079	0.5545177
LOC1025549	0.2854827	1.2606881	0	0.1255224	0.0855115	0
Rasd1	0.9862211	0.6346674	0.401946	0.4243486	0.4080452	0.4371398
Pnliprp1	0.0239677	0.0757029	0.1062299	0	0.0113857	0.0066081
Stox2	0.1718648	0.1940931	0.1980927	0.3172837	0.3232847	0.3482778
Ppp1r1b	1.6546507	1.2502563	1.3969714	0.9760188	1.2663941	1.2687971
Slc25a40	0.7081744	0.6547707	0.6517108	0.9519076	0.897452	0.9672762
LOC1009107	0.187178	0.0494318	0.2734986	0.4724793	0.4106054	0.4864947
Gpr17	0.0517771	0.0438493	0.0196898	0.1153743	0.1321547	0.1674528
LOC1009100	0.1907022	0.1822935	0.1597366	0.3393402	0.3094961	0.3431783
Nxph4	1.0088546	0.9028899	0.8877871	0.6308839	0.651658	0.7617057

LOC108348(	0.4015331	0.2512757	0.492896	0.1871225	0.1382149	0.1220289
Top2a	0.0811837	0.2032762	0.0886356	0.3294127	0.2927642	0.2837025
LOC108352E	0	0	0	0.0320844	0.003589	0.043509
Fam212a	0.4628466	0.4922094	0.5183285	0.2172496	0.2744668	0.2435323
Cd109	0.018333	0.0123909	0.0143918	0.0556752	0.0574004	0.0603709
LOC102551(	0.453151	0.463437	0.5297904	0.6203626	0.6887698	0.8319013
LOC500028	0.4765465	0.3678431	0.4459934	0.700978	0.823939	0.7476718
LOC103693(	0.3873088	0.4080822	0.3143092	0.6272315	0.6599614	0.6362023
Lnp	0.2143134	0.3851594	0.3823307	0.5334704	0.5332499	0.6190417
Ttpa	0.1829944	0.2003727	0.2084023	0.364448	0.4153639	0.3956498
Rbms3	0.3485004	0.4129573	0.2996957	0.5425049	0.5114085	0.6482264
LOC108348l	0.0218578	0.0147925	0.0255125	0.0732012	0.078514	0.0774676
Slc22a7	1.2638937	1.1031221	1.0369042	0.4833001	0.9974918	0.8109157
LOC102547:	0.5412464	0.5057219	0.4764457	0.7861105	0.7341732	0.7834434
Fam111a	1.1588571	0.1392286	0.0522916	0.177156	0.1501869	0.1582624
Mki67	0.0864944	0.1960203	0.1110081	0.2999078	0.243171	0.2644358
Adamtsl3	0.1956852	0.2334354	0.2337618	0.3342007	0.3787659	0.4082199
Trim66	0.1720729	0.3027983	0.3488585	0.39279	0.5205145	0.4656151
Vwa5a	0.1515268	0.1846185	0.14081	0.191192	0.7448926	0.2230857
Gas2l3	0.0066144	0.0092219	0.0359087	0.0894331	0.0528196	0.0950556
LOC690471	0.6884674	1.2479062	0.2862441	0.196974	0.3259238	0.4381141
LOC691135	0.1833455	0.2187432	0.2122834	0.3695243	0.3811526	0.440914
Intu	0.1463192	0.2014764	0.1992655	0.3851955	0.3385009	0.4245796
Fut9	0	0.0552359	0	0.1913276	0.0954997	0.1922649
Mamdc2	0.1573678	0.1111537	0.2223305	0.3705327	0.3404611	0.3610319
LOC102553(	0.1975569	1.3205124	0.080842	0.2052138	0.0207879	0.1788025
Syne4	0	0.0641422	0.0630842	0.1545347	0.3918663	0.1531663
LOC1025481	0	0.0081555	0.0311986	0.092081	0.0927141	0.0696646
Mir146a	0	0	0	0.3221054	0.2687495	0.3950637
LOC685406	0	0	0.0268374	0.2114712	0.1157824	0.1742798
Ocm2	0.0704802	0.283275	0	0	0	0
Cntnap3b	0.0224107	0.0051157	0.0121836	0.0693286	0.0361395	0.0624127
LOC100911:	1.4149536	0.0256869	0.2917259	0.1519328	0.3053342	0.0918342
Slpi	0.041328	0.3257006	0.1222384	0.0269336	0.013326	0
Cxcl14	0.4194824	0.5187432	0.4304752	0.2429756	0.1431172	0.2790223
Eda2r	0.0182057	0	0	0.0473644	0.0554895	0.0634579
Csrnp3	0.2666228	0.2399434	0.2457825	0.4398285	0.506873	0.4406594
Fam60a	0.2279227	0.1730737	0.3375837	0.4918541	0.5339383	0.4430962
Inpp5b	0.4183458	0.2469682	0.2531792	0.6212667	0.4837241	0.5321155
Alkbh6	0.3583934	0.1969688	0.1349455	0.0547976	0.0857613	0.0316538
Fbxo32	0.5686096	0.5010911	0.5020346	0.7737639	0.8444906	0.816527
Tnks2	0.056895	0.2184777	0.1276941	0.1204312	0.3128226	0.6881493
Apba1	0.055309	0.1108178	0.0921928	0.2328852	0.2267595	0.1953653
Pdgfd	0.3216387	0.4713338	0.3787016	0.6465744	0.6315803	0.7329926
Camk2n2	1.1750899	0.850972	1.0706447	0.727088	0.524464	0.7213588
LOC687780	0.880333	1.0198462	0.9041894	0.5533274	0.6903949	0.5802332
B3galt4	0.6600373	0.7065355	0.7402532	0.4243667	0.477291	0.4957208
Lacc1	0.1534382	0.1881603	0.1624535	0.3418215	0.2569958	0.3126261
Mcpt1	0	0	0	0.1513432	0	0.0957725
LOC100912(	0.0711906	0.0625781	0.0624539	0.6799719	0.0669897	0.1278419
Mfap2	0.5173004	0.5190608	0.4788323	0.2270868	0.1960855	0.3295642

Fosb	0.5768051	0.1136503	0.096255	0.0880989	0.0631345	0.0928705
LOC1083481	0.5297014	0.6953167	0.7135229	0.4192643	0.4467896	0.4360498
LOC1003621	0.079101	0.0539519	0.0831711	0.2166077	0.2029061	0.1325664
Chml	0.1070397	0.1631608	0.1525634	0.3285177	0.3064258	0.3365916
LOC1083531	0	0	0	0.0302585	0.0703008	0.1478856
LOC1083482	0.0089556	0.0197881	0	0.0719758	0.1114736	0.0635744
LOC1003632	0.5968808	2.0779102	0.0693151	0.1952493	0.7298172	0.0987517
LOC1083481	0.2080687	0.1589719	0.1349748	0.827256	0.270265	0.2568819
Prrg1	0.1875978	0.2107384	0.2358436	0.4137495	0.353929	0.4110686
LOC502834	0.4526432	1.4403341	0.1476375	0.3634529	0.2494908	0.3062171
Clgn	0.0228273	0.0143281	0.0125364	0.0540678	0.0978326	0.057891
Pfn2	0.1556265	0.1847876	0.1775809	0.5524822	0.369531	0.2476282
RGD1561812	0.0389156	0.0328829	0.0288466	0.111555	0.1479732	0.0708949
Hmmr	0.1339324	0.1821597	0.1222151	0.3418463	0.3497091	0.2889871
P2ry4	0.0334293	0.0891468	0.073622	0.1647967	0.1713081	0.1758763
Galnt5	0.0106182	0.002996	0	0.0275243	0.0172654	0.0960269
Ciart	0.5104169	0.4604845	0.7398942	0.1315578	0.3204209	0.3603958
Lilra5	0.3314397	0.4790036	0.3645818	0.6620462	0.6416146	0.6407811
LOC1009118	0	0	0	0.0332545	0.016514	0.1032707
Zfp763	0.1964162	0.2784225	0.2680157	0.4128648	0.4058943	0.5156129
Znf761	0.2024657	0.2394559	0.2368339	0.348012	0.3891377	0.4721697
Bmp3	0.3538675	0.5524933	0.4220449	0.683373	0.8195916	0.6048614
LOC1009119	0.1474592	0.1108473	0.1079173	0.6596485	0.5218463	0.0450294
Dnajb7	0.034479	0.0309843	0.020538	0.0872127	0.1468429	0.1549384
LOC1083531	0	0	0.0090774	0.0304984	0.0458524	0.0388138
Flrt3	0.2039489	0.257218	0.2737425	0.3504347	0.4480785	0.5749007
Zfp36	1.9783431	1.6297181	1.3737871	1.4180959	1.353394	1.5022899
LOC1009117	0.6522767	0.4027667	0.2553833	1.6960457	0.5909146	0.2359625
LOC1025521	0.475554	1.213988	0.0992075	0.2077251	0.3272679	0.1085562
Noxo1	0.0209247	0.2521014	0.0204507	0	0.0099219	0.0114336
LOC1025563	0.7222582	0.6475409	0.6139056	0.404838	0.4936875	0.4414614
RGD1559600	0.4037461	0.5548713	0.4475876	0.7401848	0.743449	0.6869288
Asb15	0.2096757	0.1992901	0.3312186	0.4559197	0.4779437	0.5046501
LOC1009123	0.0040621	0	0.0019887	0.0210583	0.0150224	0.0215144
Micb	0.139772	0.1537351	0.0834728	0.3065023	0.3494215	0.3045229
Tspan4	0.8760957	0.7757297	0.3203629	0.3970745	0.4040899	0.2576679
Zfp82	0.0022606	0.0149532	0.0109309	0.0551684	0.0519429	0.035275
Emid1	0.9770406	0.8156373	0.8162758	0.6746443	0.5356209	0.6951562
LOC1009094	0	0	0	0.036318	0.0239265	0.0275056
Ppm1e	0.0487661	0.0261413	0.0164872	0.120526	0.1250931	0.0933404
LOC1083481	0.0218578	0.0902529	0.0611739	0.6977565	0.0825825	0.060237
Cpa3	0	0	0	0.0590223	0	0.095353
Myct1	0.3392647	0.4435116	0.3254701	0.6149015	0.6146154	0.643734
Ceacam20	0.533161	1.26265	1.068084	0.4722429	0.7681341	0.6739963
Pla2g2a	0.2146179	1.0090114	0.2365914	0.1516122	0.1698985	0.2556052
LOC1025521	0.0795408	0.1632602	0.1082096	0.2213813	0.3026355	0.284772
Bche	0	0.0219254	0.0284972	0.0711352	0.0773763	0.1303995
Zc3h12b	0.01154	0.0097033	0.0056745	0.0427888	0.0442096	0.042131
Uts2r	0.0534712	0.0432441	0.0989783	0.0153536	0	0
LOC1036900	0.9945597	0.1320566	1.0904766	0.0583099	0.1130825	0.5188321
Mif	0.861886	0.9250416	0.8718905	2.0350604	0.7274261	0.7718294

LOC102550[illegible]	0.5705721	0.1236777	0.3606906	0.0714393	0.1866699	0.1415549
LOC108348[illegible]	0.0932753	0.205623	0.0694284	0.3562064	0.2058854	0.4263256
Nefh	0.2085386	0.1515301	0.1816188	0.0736506	0.0757688	0.0819988
LOC103690[illegible]	0.4830877	0.6755294	0.5685321	0.8863935	0.7729724	0.8777703
Nav3	0.0442847	0.0772768	0.0343024	0.1448968	0.1015791	0.1665087
Hlx	0.7561483	0.5331031	0.5207865	0.3510026	0.4012841	0.3859578
Mcpt8l2	0	0	0	0.0874336	0	0.1516605
Parpbp	0.0048589	0.0187227	0.0094423	0.0488173	0.0696013	0.0379069
Tmem236	0.3753183	0.176357	0.276305	0.5745712	0.4518951	0.6594646
Car3	0.3702309	0.8945125	0.1773382	1.1592683	0.6447545	1.3801805
Hspb2	1.0418232	0.9667555	0.9580559	0.6552066	0.7370447	0.7646621
Kif18a	0.0542739	0.0741397	0.0844138	0.2113892	0.1477006	0.1853616
Serpine1	1.0022874	0.751524	0.6205525	0.5698822	0.4751258	0.6609796
Upk3b	0.0236473	0.0592134	0.0450582	0.2256647	0.0699107	0.2616533
LOC103694[illegible]	0.949346	1.2087317	1.2014502	0.7136073	0.8144688	0.9589598
Pcdhb22	0.0540295	0.0815025	0.0492806	0.1518072	0.1800945	0.1377729
Mblac2	0.4277263	0.4112992	0.4858111	0.6345342	0.6936032	0.713975
Capns1	1.8235348	1.7648626	1.8156637	1.5801291	0.7885447	1.5649977
Dusp4	0.2149029	0.1675157	0.176178	0.3685412	0.4194779	0.4185125
Meis3	0.6377286	0.6332554	0.6063893	0.4026869	0.4218913	0.4303718
Adamts7	0.3567078	0.4043459	0.3449097	0.2551358	0.1805165	0.2302308
LOC108350[illegible]	0.0022455	0.0050083	0.0087086	0.0432235	0.0225902	0.0259754
Knl1	0.0022737	0.0199376	0.0077245	0.0496439	0.0338638	0.0444607
Slco1b2	0.0118219	0	0.0058141	0.0489732	0.0218819	0.0546978
Cyp21a1	0.5771931	0.440897	0.4866475	0.268388	0.2773674	0.3346446
LOC102546[illegible]	2.0295846	2.9736243	1.1036842	1.8888383	1.7835074	1.6655777
RGD156396[illegible]	0.3926597	0.1442905	0.2898615	0.1112344	0.0767684	0.131778
RGD156323[illegible]	0.8170838	1.4931414	0.2933323	0.4231105	0.5295873	0.5744319
Cracr2b	0.8942743	1.023496	0.3793746	0.306467	0.5487211	0.5487795
Mcpt1l1	0	0	0	0.1014527	0	0.0809306
Rell2	0.4475567	0.3878218	0.383527	0.2420579	0.2098381	0.1762717
Dnah10	0.0143719	0.0119178	0.0288109	0.0036489	0.0053211	0.0054592
Rabep2	1.115671	1.3243239	1.3802396	1.0881769	0.6651274	1.157781
LOC102557[illegible]	0.0547945	0.0064849	0.053599	0.2709323	0.166097	0.0804429
Dear	0	0	0	0.0348213	0.002708	0.0123658
RGD156309[illegible]	0.2611423	0.4854367	0.3113716	0.6308166	0.4435386	0.630798
Lhcgr	0.0131159	0.0558392	0.0221859	0.1291473	0.170518	0.0603709
LOC102554[illegible]	0.1509119	0.252909	0.1086019	0.5558456	0.4075961	0.3788077
Clec1a	0.2545589	0.4457182	0.3944351	0.6390883	0.5438123	0.5646638
Galnt6	0.3603453	0.4596087	0.2633124	0.1849282	0.1514225	0.233552
Olr1584	0	0.0034362	0.0205894	0.0476774	0.0477992	0.0517829
Fam156b	0.0063962	0.0256585	0.0495175	0.1263073	0.0855204	0.0936155
Cym	0.0692535	0.0323619	0.0980569	0.0142	0	0.0080405
LOC690255	0.5100315	1.2583236	0.1258219	0.0865271	0.3278551	0.3125123
Jph2	0.3347832	0.3393893	0.2293911	0.1577669	0.1413224	0.1616391
Gvinp1	0.0114298	0	0.0025067	0.0446322	0.0165493	0.033451
LOC102551[illegible]	0.1566238	0.1468183	0.145137	0.2429342	0.2594909	0.3880017
Galnt13	0.222914	0.2007587	0.2351129	0.3685072	0.3856322	0.4643388
Akt3	0.3545659	0.4283412	0.3284499	0.6284346	0.6078322	0.6096052
Hdac9	0.1340547	0.2753703	0.2092978	0.3687127	0.3777136	0.387266
LOC100361[illegible]	0.5034753	1.5295594	0.075529	0.2186034	0.4573243	0.3441153

Tmem86a	1.5086575	0.9572052	1.0706577	0.8041542	0.8716654	1.0856909
Fcgbp	0.1906063	0.2198783	0.1748073	0.0789179	0.1066606	0.1131988
Lrfn3	0.5678765	0.3607009	0.5789357	0.0748604	0.2486566	0.3541019
Htr2a	0.0347329	0.0132792	0	0.084015	0.1469928	0.0606947
LOC1025537	0.1087968	0.7466462	0.0540077	0.096075	0.0893681	0.0810381
Prdm10	0.1351914	0.1953586	0.1663299	0.3088422	0.3165243	0.2414181
Slc25a36l1	0.0425958	0.0584675	0.0366604	0.10463	0.1709806	0.168319
RGD1561247	0.0927725	1.2232112	0.0477857	0.1463207	0.0671598	0.1803613
LOC1083531	0.1231356	0.0731189	0.137852	0.2679781	0.3004971	0.2616804
Ccnb3	0.027566	0.0782309	0.0465927	0.0873907	0.1935881	0.165282
LOC1025553	0.0222181	0.3006578	0.0423967	0	0	0
Mkx	0	0	0	0.0037129	0.007204	0.0164554
Rufy2	0.2972698	0.1709683	0.1926642	0.5061699	0.4312882	0.3857464
LOC1003653	0.2131524	0.30052	0.3012438	0.4467223	0.4641451	0.4968163
Soat2	0.0639141	0.027967	0.1107186	0.020207	0.0079852	0
LOC1009115	0.0170521	0.2219396	0.0728165	0.0217373	0.0211867	0.0282989
LOC1083524	0	0	0	0.0052641	0.0012878	0.0175075
LOC1009108	0.2048956	0.0824726	0.1051342	0.3004449	0.2760675	0.3172136
RGD1359290	1.9179917	1.964209	2.2240006	1.7074031	1.7708752	0.7741512
Kcnma1	0.0849584	0.0698822	0.0887356	0.1760987	0.2169967	0.1547794
Cep295nl	0.0052936	0.0117562	0.0051715	0.0391401	0.0171125	0.0690466
Slc2a12	0.3837316	0.6013155	0.4485973	0.6788932	0.8955979	0.541017
Senp1	0.1553924	0.1928479	0.1443716	0.323005	0.26693	0.2935239
LOC500174	0.2590299	0.9224691	0.1275754	0.1931361	0	0.0751516
Stk26	0.1833088	0.2636012	0.200632	0.3792783	0.4168021	0.3849803
Calcr	0.2024649	0.1871087	0.2537921	0.3307857	0.3714745	0.4781257
Armcx6	0.3295648	0.2034753	0.3514605	0.1579794	0.1470773	0.1541342
Lrriq1	0.0080551	0.0195631	0.0047391	0.0359817	0.0392774	0.0544409
Ankar	0.0122774	0.0091895	0.0013494	0.0525502	0.0288193	0.0275242
Gnrh1	0.2965103	0.3722527	0.2707902	0.7425336	0.6219517	0.4866576
LOC1036920	0.6188574	0.5051354	0.471712	0.8011201	0.8822784	0.6813329
LOC691828	0.5599234	1.2674033	0.0351231	0.3175693	0.2472526	0.1929171
LOC1083503	0.0818712	0.0906417	0.0980569	0.1898867	0	1.0427548
Fbxo48	0.0527961	0.0606865	0.0896057	0.1476753	0.1648666	0.17516
Hcrtr1	0.1290655	0.2029294	0.1843293	0.0839487	0.0667615	0.0397755
Abcg3l4	0.2194448	0.258387	0.2124409	0.4500042	0.365041	0.472736
Cdcp2	0.0057117	0.024995	0.097661	0	0.0053503	0.0061704
Cmc1	1.1021718	0.9709727	1.3205348	0.7888726	0.9060217	0.9139324
Dnph1	1.02077	0.9381459	1.1547731	0.7268271	0.7475802	0.8111402
Zmat4	0.1894506	0.2120834	0.1844044	0.2988798	0.2601333	0.5671998
LOC1083495	0.024276	0.0630309	0.0302761	0.0936984	0.1215516	0.1160292
Sgcd	0.0454441	0.0793754	0.0847558	0.1922052	0.1695861	0.2190016
Ptgis	0.4212772	0.4450889	0.4302943	0.2489959	0.1937827	0.2685182
Gucy2e	0.0777898	0.0837156	0.0847776	0.1659922	0.2017232	0.1976804
Vsig4	0.0259449	0.1554695	0.0314757	0.006378	0.0123397	0
Gimap1	0.5093287	0.5448242	0.4585304	0.3459409	0.2360554	0.3326637
LOC691851	0.1140865	0.2905427	0.0207383	0	0.0198983	0
Dfnb59	0.6852214	0.5630386	0.606076	0.3372224	0.3700495	0.4916428
Trmt13	0.2149945	0.2547303	0.2671774	0.4418471	0.4236123	0.4769419
Cfap47	0	0.0472012	0.0212401	0.0811762	0.1045758	0.1453478
LOC1025564	0	0.006642	0	0.0224155	0.0164883	0.0281661

Nr4a1	1.483045	0.780346	0.5525936	0.7670757	0.6439607	0.6904895
LOC1003631	0	0.0063868	0	0.0370711	0.0361479	0.0241796
Il1a	0	0.2202036	0	0	0	0
Cyp2d3	0.1293854	0.2309237	0.1709747	0.0717494	0.0746249	0.0417856
Cxcr3	0.4900674	0.4879698	0.4247765	0.3310391	0.2039932	0.2247832
Zfp367	0.2482185	0.290696	0.252746	0.4442228	0.4063043	0.4545677
Mzb1	0.2936658	1.1863748	0.1643165	0.295917	0.2798596	0.2430193
RGD1565119	0.009554	0.015915	0.0361923	0	0	0
Zgrf1	0.0515966	0.0573349	0.0946515	0.1614929	0.1511335	0.1489307
RGD1565622	0.1070496	0.1377473	0.1137656	0.227654	0.2506564	0.2370668
Pcdhb11	0.0302869	0.0421352	0.0394506	0.0864903	0.1009539	0.0925812
LOC1036945	0.1811335	0.2250421	0.2335837	0.3579109	0.3376941	0.3970809
Ccr5	0.3161387	0.5571811	0.2867299	0.7069855	0.6041908	0.6933056
Ccl5	0.8962219	1.1212216	0.6680099	0.6625545	0.5702809	0.5411848
Ccl19	1.1261299	1.5251178	1.028503	0.958701	0.9934544	0.9899527
Slc9a7	0.1193723	0.1068869	0.0756066	0.1886126	0.2491263	0.2380241
LOC1009098	0.2280807	0.1348716	0.0881047	0.0410845	0.0685519	0.049275
Fzd10	0.042216	0.022551	0.0490876	0.0084413	0.0055001	0.0094802
Alox15	0.2611217	0.5346484	0.3790184	0.2127752	0.2310953	0.1645203
Tlr5	0.0487238	0.0713754	0.049207	0.1278777	0.1324202	0.1414461
Trappc3l	0.0836546	0.0668298	0.1037171	0.1963853	0.2954807	0.2216379
Pappa2	0.0150233	0.0200513	0.0279488	0.0490432	0.0686957	0.0576787
Cyp46a1	0.1856203	0.0761897	0.3877098	0.0829803	0.0612942	0.0849885
RGD1561327	0.0132354	0.0147805	0.0097357	0.0545593	0.0390838	0.0702599
Kcnb2	0.0034718	0.0153249	0.0084299	0.0322779	0.0284235	0.05645
P2rx1	0.5033196	0.530972	0.4004237	0.2718506	0.2075051	0.3576606
Mfsd14a	0.0300811	0.0679576	0.0449222	0.0898373	0.2548806	0.1086468
Zfp945	0.1261786	0.1885284	0.1827133	0.335995	0.2924701	0.3026558
Trank1	0.0184887	0.0250875	0.0200307	0.0557734	0.0500571	0.0641986
Mmp20	0	0	0	0.0293922	0.0049089	0.0056618
RGD1563433	0.7809069	0.7750506	0.3276801	0.2730615	0.3411502	0.338655
Comp	0.1887471	0.1449868	0.1067093	0.0379703	0.0590435	0.0674838
LOC691422	0.1652619	0.1967435	0.1954572	0.2692196	0.3287676	0.3785447
LOC1083480	0.0397598	0.071425	0.0550992	0.1146374	0.1411384	0.161083
RGD1560556	0.0173154	0.0570222	0.0196788	0.1483863	0.0331606	0.149326
LOC1036923	0.2463137	0.331198	0.2788184	0.4977189	0.4956106	0.5086511
Ly75	0.1145216	0.1899202	0.0550289	0.3139705	0.1912471	0.3051751
Tlr8	0.1103312	0.3354313	0.1544013	0.4572625	0.3740373	0.4328134
Nrn1l	0.7790298	0.5202853	0.9430896	0.4850484	0.405255	0.4996109
Trpm3	0.0492068	0.0932158	0.0662132	0.1419189	0.1261309	0.2086651
Slc22a13	0.6237076	1.0141761	0.3450837	0.5214144	0.3672466	0.188513
Slitrk2	0	0	0	0.002531	0.0073549	0.0056713
Cdh22	0.2051066	0.0538948	0.1228267	0.0442351	0.0258936	0.0548994
Oas1f	0.0405329	0.1670297	0.3148701	0.0748431	0.0221563	0.0065111
Unc13d	0.3450663	0.514076	0.3445999	0.2592385	0.2035025	0.2697654
Klc3	0.6282769	0.5079093	0.6344154	0.3841385	0.4166074	0.3292201
Podnl1	0.1195422	0.1168568	0.0932912	0.0407042	0.0082345	0.045512
Fzd9	0.5597117	0.5400637	0.7074585	0.3205307	0.495132	0.3296799
Cst6	0.159094	0.3901862	0.2269559	0.0792317	0.0947092	0.1076515
Enam	0	0	0	0.0084616	0.0041416	0.0141777
Nobox	0.0330998	0.0333184	0.0197038	0.0811837	0.0765662	0.11381

Pcdhb4	0.0252602	0.0509315	0.0246907	0.1042821	0.0675271	0.156741
Igkv28	0.9009518	1.7251314	0.2209819	0.5092555	0.6778032	0.7424478
LOC1083501	0.1949974	0.1689466	0.1454755	0.3235858	0.3278998	0.4288799
Abcd2	0.0233162	0.0187313	0.0131685	0.0808077	0.0294192	0.0761388
Tekt2	0.066774	0.0683499	0.0446307	0	0	0.0252575
Havcr1	0.2020364	0.4637281	0.6411397	1.1085156	0.8289792	0.3929831
LOC100362(	0.9011306	0.9709996	0.7983923	0.6217844	0.6254173	0.6061787
Ttk	0.0249391	0.0425196	0.0347864	0.0821846	0.1018885	0.1061361
LOC100909!	0.2272666	0.2014896	0.1282798	0.3771994	0.329987	0.4625807
Lemd2	0.1693248	0.2208242	0.1446313	0.1249421	0.9824683	0.1382458
Ect2	0.0960169	0.1182372	0.1571844	0.2568682	0.2744778	0.2238665
Spaca5	0	0.0365989	0.0762492	0.1270965	0.2207101	0.2140821
Zfp136	0.1928252	0.2014715	0.2613804	0.3741701	0.2651785	0.5082887
Krt15	0.1631572	1.6326385	0	0.2339731	0.0104685	0.294831
LOC1083517	0.765882	0.7962591	0.8884342	0.54426	0.5852356	0.5375045
Idi2	0.0556355	0.031994	0.0280633	0.2832255	0.0989697	0.275626
LOC100912(	0.2217709	0.9618462	0.4134876	0.2884849	0.2907563	0.260144
Zfp683	0.0455172	0.023228	0.0397873	0.010132	0	0
Adgrf1	0.232157	0.3396329	0.2435201	0.4702763	0.4645218	0.5083114
Tmem241	0.2668672	0.3939189	0.3060991	0.5285332	0.5034895	0.5649943
Kcnj11	0.2401664	0.1776271	0.0952096	0.0664777	0.0623568	0.079719
Tbxa2r	0.5836523	0.5615927	0.5168392	0.3645135	0.3954289	0.3021809
Fam110d	0.7136421	0.7844711	0.7250015	0.3826391	0.56508	0.554764
Rundc3a	0.53927	0.4825972	0.4059398	0.2682881	0.3130133	0.2819944
Cyp3a18	0	0.0105098	0.0356497	0.0393004	0.1265435	0.1004837
Zfp428	0.6196194	0.5095077	0.5694655	0.3317197	0.4077181	0.3573048
Foxd3	0	0	0	0.0126021	0.0122797	0.0106516
LOC691387	0.0030597	0.0011435	0.0019949	0.0078004	0.012283	0.0130765
LOC100912:	0.3072149	0.2702409	0.3198477	0.3877597	0.4780826	0.9078577
LOC290209	0.0054732	0	0.0261002	0.0546524	0.0946223	0.0343269
Fam115c	0.3374266	0.2880321	0.2779165	0.4519413	0.422721	0.5818067
LOC691153	0.1280422	0.2433442	0.2041487	0.0911319	0.0441559	0.0794556
Trhde	0.0630321	0.0896759	0.0815676	0.1698511	0.1382309	0.180999
LOC100359(	0.261915	0.2250725	0.2675629	0.6247555	0.4822695	0.2856658
LOC679087	0.0735933	0.093777	0.0418346	0.1480219	0.1536306	0.161548
Lgr6	0.1932383	0.3833884	0.3978146	0.1390724	0.2161785	0.1983078
RGD1566337	0	0	0	0.0574866	0.0400081	0
Edaradd	0.1173722	0.1820499	0.1739288	0.2943425	0.2743247	0.3903889
LOC691485	0.0133054	0.0292254	0.0287242	0.071675	0.0421641	0.1575038
LOC500194	0.7933429	1.7146812	0.1478652	0.5075285	0.7115747	0.5846232
Trpc7	0	0	0.0025906	0.007604	0.0146924	0.0196736
LOC499565	0	0	0	0.0647158	0.1006683	0.0721331
Plin1	0.0263433	0.1486341	0	0.3184908	0.077433	0.4954322
Tmem52	0.7118027	0.5941062	0.5533968	0.3460385	0.4162886	0.4097405
LOC100912!	0.0555699	0.0617196	0.0644589	0.5866438	0.0619696	0.0920422
Pdilt	0.0800119	0.0969586	0.100373	0.0953922	0.2618466	0.366137
Itgbl1	0.1797407	0.1849878	0.139946	0.3397271	0.2616665	0.397625
Ubap1l	0.6145327	0.5309094	0.6358692	0.4586324	0.3854779	0.3292621
Gpr21	0.0040231	0	0	0.0336314	0.0362859	0.0043469
Lrfn1	0.2146179	0.2325287	0.1842011	0.1003491	0.1231976	0.0789213
Mas1	0.0211924	0.0236413	0.0318063	0.0991206	0.0802302	0.0515854

LOC1003597	0.0056215	0.0062842	0.0189255	0.0439324	0.0331387	0.0545283
Elovl6	0.0728965	0.0701307	0.0421863	0.130773	0.2093285	0.1416622
Jun	1.9844632	1.367643	1.3561644	1.3397005	1.278294	1.462257
Cnr1	0.1353592	0.083154	0.1639835	0.1705756	0.2673272	0.357443
LOC1025552	0.1064935	1.006039	0	0	0.1180121	0
Ccdc18	0.0333809	0.0744084	0.0656452	0.1300409	0.1527889	0.1582504
LOC1003646	0.0076367	0.0169046	0.0147961	0.0555394	0.0966153	0.0546978
Fam49a	0.1852059	0.2082999	0.2231688	0.3708026	0.3722664	0.370675
Mir568	1.3694031	1.641453	1.4766288	1.8970802	1.7149039	1.835881
Serpine3	0.0147432	0.0191447	0.008462	0.0666858	0.0538545	0.0223766
LOC1025544	0	0.0155937	0	0.0806927	0.0618248	0.0150752
Eppin	0.0250827	0.027967	0.0124315	0.1461508	0.1066499	0.0765654
RGD1565987	0.010356	0.0944632	0.0296748	0	0.009704	0
Spns3	0.7005092	0.4314447	0.3230976	0.276105	0.2414984	0.3563022
LOC1009124	0.0979059	0.1170775	0.1091057	0.2203354	0.2254937	0.2327248
Asphd2	0.0068521	0.0262341	0.0066944	0	0	0
Btc	0.6719956	0.8356282	0.7443808	1.0487661	1.1179955	0.9091261
Mcmdc2	0.0937409	0.1233528	0.1205391	0.1993648	0.1863167	0.2738905
Oprd1	0.116182	0.0357435	0.103682	0.0308872	0	0.0141645
Mpc1l	0.0647057	0.1354929	0.07832	0.2400207	0.1876457	0.1965699
Mc1r	0.065111	0.0376158	0.0706221	0.1251006	0.1295089	0.16365
Eid3	0	0.0078904	0.0398303	0.0329285	0.1111126	0.1749289
Aqp8	0.198282	0.3398071	0.3593489	0.1418482	0.1434197	0.1405749
Syt1	0.0271036	0.0509354	0.0536642	0.1529203	0.095751	0.1023536
Kcna2	0.0240358	0.0405787	0.0055354	0.1135264	0.0787399	0.0411391
Pik3c2b	0.1421936	0.2113441	0.1219976	0.3125904	0.2286662	0.3258845
LOC1025471	0.1254923	0.1300006	0.0594515	0.2862018	0.2085994	0.2520524
Bhlha15	0.1491955	0.1672933	0.1010596	0.0682856	0.0595239	0.057751
Adcy10	0.0288706	0.0763653	0.0560054	0.1163579	0.1867886	0.0978147
Kcnv2	0.0703161	0.0825362	0.1004684	0.1701422	0.2154595	0.1660539
Gdf15	1.225254	0.6823217	0.6806853	0.6379057	0.6291415	0.5374086
Spem1	0	0.0569002	0.0731105	0	0	0
LOC1036948	0.1226578	0.0995826	0.0711975	0.0527615	0.0684679	0.7690958
RGD1565779	0.2363357	0.2877041	0.6373333	0.2405613	0.1953284	0.1105843
Cd84	0.1279743	0.2904411	0.1544481	0.4301587	0.3001159	0.4628633
Hmga2	0	0.0129217	0	0.0628445	0.0516855	0.047942
Vom2r44	0.0975263	0.0463655	0.042906	0.2384116	0.1422861	0.116749
Trim43a	0.0304304	0	0.0239594	0.1021355	0.060514	0.0972741
Ccdc155	0.0198222	0.0148689	0.0318235	0	0	0.0072465
Pgbd1	0	0	0.0223357	0.041457	0.0418571	0.0762328
St18	0.0028579	0.0031959	0.0137829	0.0318761	0.0273125	0.0241117
Fam188b2	0	0	0	0.0237069	0	0.0392665
LOC502812	0.3896279	0.6785356	0	0.0672804	0.0855115	0.0749671
RGD1564381	0	0.5134185	0.0452057	0	0.022254	0
Zfp70911	0	0.0152249	0.0053782	0.0333062	0.0276328	0.0427384
Ccl11	0	0.1049782	0.0361568	0	0	0
Serpina1	0.0123479	0.2119626	0.1740586	0.2322196	0.8016275	0.1572812
LOC1003628	0.0071663	0.0062423	0.0077725	0.0269313	0.0262531	0.0145076
Gp5	0	0	0	0.0103124	0.0067243	0.0228564
Akain1	0.3116497	0.2305833	0.2164599	0.4333754	0.3809054	0.5105715
Slc24a2	0.0021396	0	0	0.0081701	0.006973	0.0057577

Ism1	0.4628042	0.3595286	0.3223181	0.6132657	0.5704541	0.7439957
Rnase12	0.0480291	0.0933662	0.054331	0.0080612	0.0078539	0.0179228
Cenpm	0.1753275	0.2480778	0.1664725	0.080004	0.071556	0.0816234
Nfatc2ip	0.3856168	0.3622894	0.4242458	0.6421785	0.6375517	0.5501863
Srm	0.8663187	0.805615	0.8156064	0.5472179	0.7230058	0.4147246
Col24a1	0.0528939	0.0192329	0.0377982	0.120259	0.0655652	0.121803
Ncf2	0.1719866	0.2612021	0.1256464	0.0825623	0.0777678	0.0854049
LOC362382	0.1519551	0.8683715	0	0.0213284	0.0777746	0.1082255
LOC1025513	0.0045464	0.0050831	0	0.0129839	0.0249455	0.0511994
Kcnip3	0.5036874	0.4173016	0.6111407	0.3273529	0.2951211	0.3592194
LOC1036905	0.1551343	0.2017123	0.0884264	0.3436817	0.3496514	0.3722657
Mum1l1	0.0535254	0.0554762	0.0576086	0.1189957	0.0861135	0.2020542
Atp1a3	0.0768961	0.1563684	0.0921985	0.0386603	0.0354366	0.0534074
LOC1025514	0.0112444	0.0125604	0.019935	0.0367278	0.0341729	0.0820333
LOC1083480	0.0115797	0.0304187	0.0068241	0.0819966	0.2033359	0.0025296
Zcchc16	0.1397141	0.1536724	0.099703	0.2616271	0.2109	0.288908
Tnfsf14	0.0773304	0.2256628	0.0756844	0.0388883	0	0.0330206
LOC688442	0.0183153	0.0204395	0.0753056	0.0665591	0	0.6679042
LOC680933	0.295242	0.2881232	0.3566562	0.5640414	0.5576298	0.4609746
LOC1083481	0.5558881	0.0912973	0.1642357	0.0855667	0.1226555	0.0951241
LOC1025540	0	0	0	0.0809148	0.0376953	0
Macc1	0.0286644	0.0786232	0.0644401	0.1441143	0.0921502	0.2249164
Mir30c2	0.099923	0	0	0.3965672	0.1715691	0.5101454
Dcp2	0	0.0773569	0.0221726	0.0951169	0.1505196	0.19855
Bcl2	0.412482	0.4364961	0.3874501	0.6202948	0.655203	0.6341896
Cidea	0.8363269	0.6105809	0.9205733	0.4654404	0.5659799	0.6122581
Ccdc36	0	0	0	0	0.022695	0.0260954
Nfe2l3	0.0116926	0	0.022557	0.0634808	0.0181117	0.1316036
Snx20	0.4163158	0.5752958	0.3988607	0.2692253	0.290368	0.2934996
RT1-DMa	0.2352861	0.7234733	0.189289	0.1289157	0.2471796	0.0611214
Mybpc2	0.4497704	0.5349281	0.6267771	0.5524375	0.5428582	1.1738092
Cadm4	0.9112628	0.7158706	0.8210827	0.4828517	0.6836447	0.5628507
Mlana	0.1544894	0.0874402	0.0670421	0.2047492	0.4367027	0.2246011
LOC1003654	0.0843799	0.1071757	0	0	0	0
Hist1h2aa	0	0	0	0.0224919	0.0627037	0.1697161
Tbc1d12	0.0961336	0.1561493	0.1428881	0.2471878	0.3264081	0.2114042
Zfp53	0.2212713	0.2949218	0.2569922	0.4047462	0.3866282	0.5077268
Mastl	0.0223828	0.0542802	0.0674073	0.1219258	0.1252452	0.1303824
Fgf10	0.264437	0.3118565	0.3250561	0.5803106	0.5570531	0.4814266
Ccne2	0.2502388	0.3360312	0.2386633	0.4842115	0.5220004	0.3593175
Btbd8	0.1100961	0.1550071	0.1405787	0.2340874	0.2255576	0.2829684
LOC1009126	0.165292	0.1931709	0.147215	0.0446567	0.0873186	0.0753371
Batf2	0.121212	0.4010287	0.0505898	0.041185	0.052767	0.0876757
Ak9	0.2915367	0.399081	0.291308	0.1522708	0.2620308	0.1633355
Sbpl	0.2422091	0.5490962	0.241681	0.0878868	0.1747029	0.2055542
Vom2r45	0	0	0	0.0163843	0.0107095	0
LOC691893	0.2110569	0.9847659	0.0871679	0.173816	0.1854443	0
Hrct1	0.6836473	0.8927701	0.8486238	0.5369847	0.6329975	0.5137948
Grin2b	0.0108965	0	0.0677267	0.0021162	0.0041132	0
Plpp4	0	0.0182963	0.0160174	0.0157673	0.1038374	0.1246944
Wdr77	0.6818924	0.5274892	0.7062504	0.3200861	0.3714043	0.566058

LOC108348(	0.0747082	0.0395927	0.0638391	0.014019	0.0236316	0.015732
LOC1025481	0.2072245	0.3646527	0.2830563	0.5212767	0.4411735	0.4544235
Prp2l1	0.0305665	0.0581122	0.0477764	0.1107213	0.0937905	0.0813063
LOC367858	0.2913042	0.3709217	0.2389234	0.1641598	0.1476199	0.0868435
Gpr25	0.0055717	0.0123691	0.0108193	0	0	0
Trim29	0.3023298	0.4667679	0.316248	0.108419	0.200804	0.2672351
Fam169a	0.1506833	0.2567552	0.3181425	0.3723957	0.4834109	0.3608048
Primpol	0.1048656	0.209946	0.1346123	0.2778924	0.2429203	0.3324909
Ogn	0.876475	1.0882607	0.9708697	0.8556835	0.783531	0.3300523
Nat3	0	0	0.0042481	0.0083219	0.0121058	0.0207532
Spocd1	0.0139033	0.0155249	0.0202226	0	0	0.0038011
S100a9	0.1485135	0.8629933	0.3170785	0.0917212	0.1157443	0.3133721
Zfp354c	0.202476	0.2250308	0.1737371	0.3308016	0.4282166	0.3586949
Kirrel3	0.048027	0.0815462	0.0977734	0.0051084	0.021959	0.0413067
LOC103690(	0.0231446	0.0381654	0.0544871	0	0	0
RGD1564074	0.2114601	0.2568644	0.2075599	0.4745396	0.3264711	0.3575699
Fam92b	0.0559515	0.028116	0.0591809	0.0147224	0.0072325	0.019213
LOC1036935	0.0591261	0.0478761	0.0655106	0.1337017	0.1609774	0.2123393
LOC1083505	0	0	0	0.0206486	0.0330415	0.0155699
Was	0.6295384	0.8135047	0.605081	0.5077641	0.3973451	0.5026106
LOC103690(	0.02155	0.1055368	0.0378657	0.0139328	0.0169058	0
Apitd1	0.798523	0.777547	0.8565986	0.5524454	0.6361631	0.5404457
Akr1c2	0.040413	0.0589827	0.0519288	0.05115	0.6789042	0
Mcf2	0.010479	0.0047206	0.012258	0.035236	0.0232038	0.0435945
Rorc	0	0.0392938	0.0128653	0.079081	0.1099856	0.0478375
Polq	0.0157478	0.0076217	0	0.0130165	0.0418881	0.0675187
Eva1c	0.3617552	0.3423249	0.390876	0.2215391	0.2021812	0.2334477
Oacyl	0.30719	0.1943352	0.2379301	0.0820039	0.1058652	0.1789351
NEWGENE_15	0.0776494	0.0078551	0.0102594	0.2278982	0.0130698	0.2786033
LOC1009117	0.481749	0.0614884	0.1046882	0.1102161	0.0597135	0.0739456
